# Condensin and topoisomerases cooperate to relieve topological stress at stalled replication forks

**DOI:** 10.1038/s41467-026-72936-1

**Published:** 2026-05-08

**Authors:** Mégane Da Mota, Axel Delamarre, Antoine Barthe, Jessica Jackson, Nail Bouzalmad, Alba Torán-Vilarrubias, Yea-Lih Lin, Cyril Ribeyre, Alessandro Vindigni, Philippe Pasero, Armelle Lengronne

**Affiliations:** 1 Institut de Génétique Humaine, Univ Montpellier, CNRS, Montpellier, France; 2 Division of Oncology, Department of Medicine, Washington University School of Medicine, St. Louis, MO USA; 3 Institut de Génétique Moléculaire de Montpellier, Univ Montpellier, CNRS, Montpellier, France; 4 Present Address: Memorial Sloan Kettering Cancer Center, Molecular Biology Program, New York, NY USA; 5 Present Address: Laboratoire d’Eco-Anthropologie, CNRS, Muséum National d’Histoire Naturelle, Université Paris Cité, Paris, France

**Keywords:** Genomic instability, Genome, Stalled forks

## Abstract

Resolving complex topological structures at replication forks is essential for faithful DNA replication, yet the underlying mechanisms remain poorly understood. Evidence from diverse eukaryotes suggests that condensin – best known for driving chromosome condensation in mitosis – may also operate during S phase to alleviate torsional stress in cooperation with topoisomerases. Here, we show in budding yeast and human cells that condensin binds stressed replication forks, where it cooperates with topoisomerases I and II to promote nascent DNA resection and restart replication. Our data indicate that condensin acts together with topoisomerase I at reversed forks to convert positive supercoils into topological DNA structures that are relaxed by topoisomerase II, enabling fork restart. These findings reveal an evolutionarily conserved role for condensin in resolving topological constraints at arrested forks, reminiscent of its function in chromosome segregation, and suggest that this activity helps prevent the formation of toxic chromosome structures during fork arrest and reversal.

## Abstract (web summary)

DNA replication forks often stall and reverse, creating topological stress that must be resolved to resume DNA synthesis. Here, the authors show that condensin, best known for its mitotic role, cooperates with topoisomerases to remodel and restart reversed forks, thereby maintaining genome stability.

## Introduction

Stalled replication forks are fragile structures that are highly susceptible to nucleolytic degradation and breakage, leading to genome instability. Cells have developed multiple mechanisms to stabilize and restart stalled forks, including replication fork reversal^1–3^. During fork reversal, the three-way junction of the replication fork is remodeled into a four-way structure by reannealing the parental strands and pairing the nascent DNA strands with each other to form a regressed arm. This reaction is catalyzed by the RAD51 recombinase in concert with DNA translocases such as SMARCAL1^4^. The resulting four-way junction is a transient intermediate that temporarily slows fork progression but promotes replication restart once the stress is resolved^3^.

Fork reversal is influenced by local topological context. In principle, positive supercoiling ahead of the fork promotes reversal^5^, while positive supercoiling along sister chromatids during reversed arm extension restrains it^6^. However, this aspect remains largely unexplored in vivo, despite recent evidence that DNA topoisomerase II (TOP2) can relieve this constraint^7^. Beyond its role in DNA replication, TOP2 is essential in mitosis, where it decatenates newly replicated sister chromatids to ensure proper chromosome segregation^8^. Condensin, a structural maintenance of chromosomes (SMC) complex that drives chromosome condensation in mitosis, enhances the decatenation activity of TOP2^9^. This regulatory interaction between condensin and TOP2 may likewise be important during interphase.

Two different condensin complexes promote chromosome condensation and segregation in vertebrates^9^. Condensin II localizes to the nucleus during interphase, associates with chromatin and acts from S phase to prophase to resolve sister chromatid intertwines^10^. By contrast, condensin I binds chromosomes only after nuclear envelope breakdown in prometaphase^9^. In the budding yeast *S. cerevisiae*, a single condensin complex mediates chromosome segregation, and its persistent chromatin association suggests additional functions outside mitosis^11–16^.

Across organisms, loss of condensin function sensitizes cells to DNA damage and replication stress. In *Arabidopsis thaliana*, condensin II mutants display hypersensitivity to DNA-damaging agents that induce double-strand breaks and replication blocks^17^. Similarly, depletion of condensin II combined with aphidicolin treatment causes severe chromosomal disintegration, underscoring its critical role in preserving chromosomal architecture under replication stress^18^. In mouse embryonic stem cells, loss of the condensin subunit Smc2 leads to increased γ-H2AX foci, indicating elevated DNA damage during S and G_2_ phases^19^. Comparable defects occur in condensin II missense mutants^20^. In the fission yeast *S. pombe*, condensin prevents transcription-induced DNA damage in G_2_-arrested cells^21^, and mutations impairing condensin function render cells hypersensitive to hydroxyurea, which slows DNA replication, and to the TOP1 inhibitor camptothecin, further emphasizing its role in mitigating replication stress^22^. Altogether, these findings suggest that condensin helps relax topological constraints during S phase, particularly when replication is perturbed. However, the molecular mechanisms underlying this function remain poorly understood.

Here, we examined how condensin cooperates with topoisomerases to relieve torsional stress in S phase, as it does in mitosis. We show that condensin is recruited to stressed replication forks in budding yeast where it cooperates with Top1 and Top2 to promote fork restart. In human cells, condensin II specifically facilitates fork processing and restart under replication stress conditions. Condensin II cooperates with TOP1 and TOP2A to drive fork slowing and controlled resection, consistent with a model in which condensin II and TOP1 act at stalled forks to convert positive supercoils into topological DNA structures that are subsequently relaxed by TOP2A. This coordinated action prevents the accumulation of DNA knots and catenated sister chromatids that could otherwise compromise genome stability.

## Results

### Condensin is recruited to stressed replication forks in an MRX-dependent manner

**Fig. 1:**
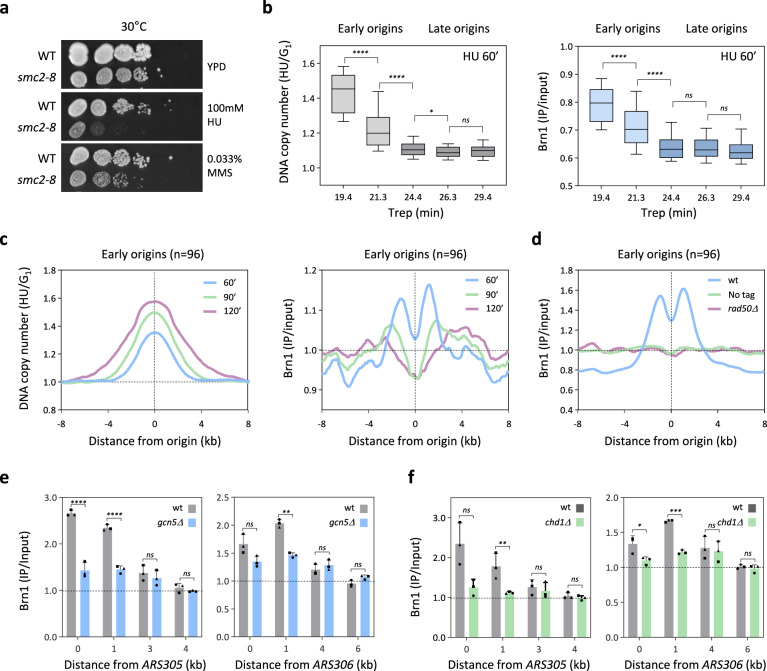
Condensin is recruited to stressed replication forks in budding yeast. **a** Serial dilutions of wild-type (WT) and thermosensitive *smc2-8* cells were spotted on YPD plates containing 100 mM HU or 0.033% MMS and were grown for 4 days at the semi-permissive temperature of 30 °C. **b** Cells expressing a tagged condensin subunit (*BRN1-PK*_*9*_) were arrested in G_1_ with a-factor, then released for 60 min into medium containing 200 mM HU. Brn1 was immunoprecipitated and changes in DNA content (left) and Brn1 occupancy (right) at all annotated *S. cerevisiae* replication origins were quantified by ChIP-seq as described^27,71^. DNA copy number and Brn1 enrichment were calculated in 2 kb windows and are shown for groups of early and late origins ordered by replication time (Trep^24^). Mean Trep is indicated for each bin. Box and whiskers show the 25th–75th (box) and 10th–90th (whiskers) percentiles. ****p < 0.0001; *p < 0.05; ns not significant (Mann–Whitney rank-sum test, two-sided). **c** Cells were released synchronously into S phase in the presence of 200 mM HU, and samples were collected after 60, 90 and 120 min. DNA content (left) and Brn1 enrichment (right) were determined by ChIP-seq in 16 kb windows centered on 96 early origins. **d** Brn1 enrichment at 96 early origins was quantified by ChIP-seq in wild type (untagged), *BRN1-PK*_*9*_ and *rad50Δ BRN1-PK*_*9*_ cells 60 min after release from G_1_ into 200 mM HU. **e**, **f** Brn1 enrichment at increasing distances from the early origins *ARS305* and *ARS306* was quantified by ChIP-qPCR in wild type (gray), *gnc5Δ* (blue) and *chd1Δ* (green) cells 60 min after release from G_1_ into S phase in the presence of 200 mM HU. Brn1 enrichment was normalized to unreplicated loci as described previously^26^. Data represent mean +/− SD of three independent experiments. Two-way ANOVA revealed a significant interaction. Post-hoc comparisons between WT and mutant at each position were adjusted for multiple testing (one family). *p < 0.05; **p < 0.01; ***p < 0.001; ns not significant. Source data are provided as a Source data file.

To assess whether condensin is required for growth under replication stress conditions, we compared the growth of *S. cerevisiae* cells carrying the wild-type allele of the condensin core subunit *SMC2* with that of cells carrying the thermosensitive (ts) *smc2-8* allele in the presence of methyl methanesulfonate (MMS) or hydroxyurea (HU). MMS impedes replication fork progression by alkylating DNA, whereas HU slows DNA synthesis by depleting dNTP pools. At 30 °C, a semi-permissive temperature for *smc2-8*, both strains displayed comparable growth in the absence of drugs. In contrast, the growth of *smc2-8* cells was markedly impaired, upon chronic exposure to either 100 mM HU or to 0.033% MMS (Fig. 1a). A similar MMS sensitivity was observed upon partial depletion of another condensin core subunit, Smc4, using an auxin-inducible degron (AID) and a low dose of auxin (IAA; Supplementary Fig. 1a).

Condensin binds multiple genomic loci throughout the cell cycle, including replication origins^12^. To further characterize its association with replication sites, we analyzed the genome-wide distribution of the condensin subunit Brn1 by ChIP-seq in cells synchronized in S phase. Cells were released from G_1_ into S phase for 60 min in the presence of 200 mM HU, which stalls replication forks 2-3 kb downstream of early origins^23^. This analysis showed that condensin is enriched at replication sites in HU-treated cells, but is absent from these sites in G_1_, indicating S-phase specific binding (Supplementary Fig. 1b).

To assess whether this association depends on origin activation, we monitored origin firing in HU-arrested cells (*t* = 60 min). Origin firing induces a local increase in DNA copy number relative to G_1_ levels, expressed as the ratio of genomic DNA reads (HU/G_1_) in 2-kb intervals centered on all annotated yeast origins (*n* = 385). These values were plotted against replication time (Trep^24^) for bins of 77 origins (Fig. 1b). Under these experimental conditions, early origins fired (HU/G_1_ ratio >1), whereas late origins, normally activated 24 min after G_1_ release, remained inactive due to replication checkpoint control, as previously reported^23,25^. Brn1 enrichment calculated over the same intervals revealed significant enrichment at early, but not late origins (Fig. 1b).

To determine whether condensin associates with replication sites during unperturbed DNA synthesis, we monitored Brn1 binding by qPCR in synchronized cells released from G_1_ into S phase. Under these conditions, Brn1 levels remained close to background (Supplementary Fig. 1c, d) and showed no correlation with replication fork progression, as inferred from DNA copy number changes (Supplementary Fig. 1e). These data indicate that during a normal S phase, condensin either does not engage replication zones or does so in a weak or transient manner that escapes detection. Collectively, our findings suggest that condensin accumulates at replication sites only after origin firing under HU-induced replication stress, rather than being stably bound to forks during unperturbed replication.

To examine whether condensin binding at replication sites changes over time under replication stress, we analyzed Brn1 distribution by ChIP-seq in cells synchronously released from G_1_ into S phase for 60, 90 and 120 min in the presence of 200 mM HU. Under these conditions, replication forks progress at ~0.1 kb/min^23^. To determine whether condensin travels with replication forks in these cells, we compared temporal changes in DNA copy number and Brn1 enrichment across 16-kb intervals centered on 96 isolated early origins (Fig. 1c). Condensin redistributed from early origins over time in parallel with fork progression, as indicated by the progressive flattening of Brn1 peaks at 90 and 120 min (Fig. 1c). This peak broadening reflects fork progression under HU rather than reduced condensin binding. Together, these data indicate that condensin is recruited to early replication origins during HU-induced replication stress and travels with replication forks.

The Mre11–Rad50–Xrs2 (MRX) complex cooperates with the histone acetyltransferase Gcn5 and the nucleosome remodeler Chd1 to promote cohesin loading by remodeling nascent chromatin^26^. To test whether condensin enrichment at stressed forks similarly depends on MRX, we synchronized wild-type and *rad50Δ* cells in S phase with HU and measured Brn1 enrichment at early origins by ChIP-seq (Fig. 1d and Supplementary Fig. 1b). In *rad50Δ* cells, Brn1 levels at early origins were reduced to background levels comparable to untagged controls. This reduction was confirmed by ChIP-qPCR at the early origins *ARS305* and *ARS306* (Supplementary Fig. 1f) as described previously^26^. We next assessed whether chromatin modifiers contribute to condensin recruitment. ChIP-qPCR analysis of Brn1 at *ARS305* and *ARS306* in *gcn5Δ* and *chd1Δ* mutants revealed a complete loss of condensin enrichment compared with wild-type cells, in which Brn1 localized just behind HU-stalled forks, ~2–3 kb downstream of active origins^23^ (Fig. 1e, f, Supplementary Fig. 1g, h). Together, these data indicate that condensin is recruited to replication stress sites through a mechanism requiring the MRX complex and chromatin remodeling, reminiscent of the pathway that mediates cohesin loading under similar conditions^26^.

### Condensin promotes fork restart after replication stress in budding yeast

In fission yeast, the condensin mutant *cnd2-1* is hypersensitive to genotoxic agents, likely because it fails to activate the DNA replication checkpoint^27^. To assess whether this checkpoint functions normally in the absence of condensin in budding yeast, we monitored the repression of late origins as a sensitive readout replication checkpoint activation^25^ in pMET3-*YCG1*-AID cells. In this strain, expression of the condensin subunit Ycg1 is repressed by methionine and degraded upon auxin addition^28^. Wild-type and pMET3-*YCG1*-AID cells were first grown in methionine-free medium to maintain *YCG1* expression, arrested in G_1_ phase with α-factor, and then transferred to rich medium containing methionine and auxin to repress *YCG1* transcription and induce Ycg1 degradation (Supplementary Fig. 2a). After release from G_1_ arrest, cells were allowed to progress into S phase for 60 min in the presence of 200 mM HU, and changes in DNA copy number at early and late origins were quantified by qPCR^25^. Late origins were repressed in condensin-depleted cells as efficiently as in wild-type cells (Supplementary Fig. 2b), indicating that condensin is dispensable for checkpoint activation at stressed forks.

**Fig. 2:**
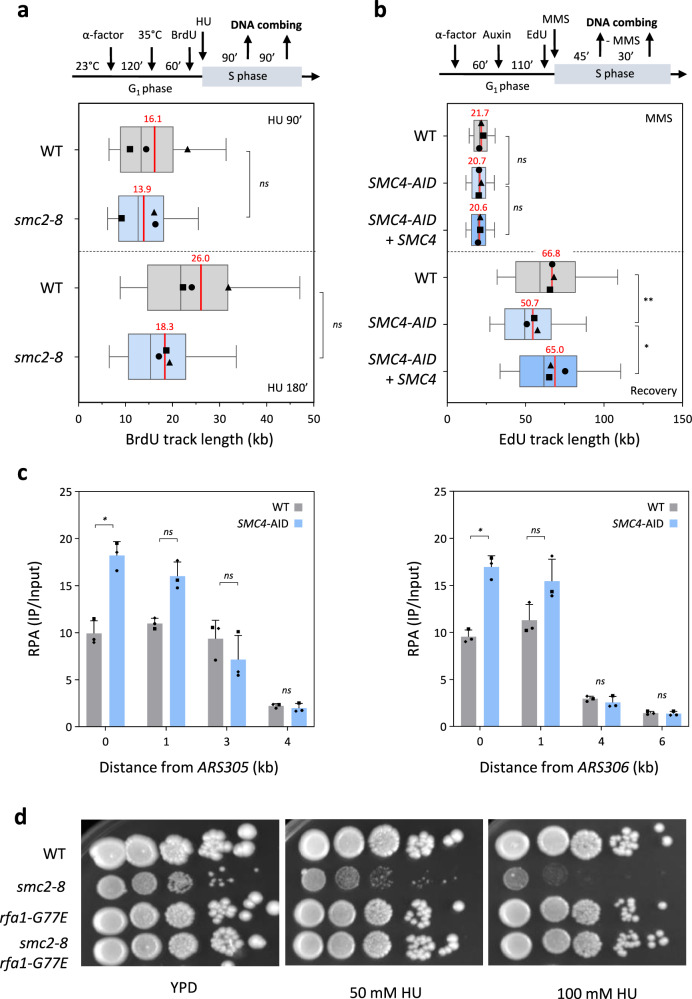
Condensin promotes fork restart after replication stress in budding yeast. **a** Wild type and *smc2-8* cells were synchronized in G_1_ with α-factor, shifted to 35 °C for 60 min and released into S phase in medium containing 200 mM HU. Newly replicated DNA was labeled with BrdU for 90 and 180 min and fork progression was analyzed by DNA combing. BrdU track length distributions from three independent experiments are shown, with box and whiskers plots indicating 25th–75th and 10th–90th percentiles, respectively. Mean length is indicated in red. ns: non-significant (unpaired t-test, two-sided). **b** Wild type, *SMC4*-*PK*_*3*_*-AID* and *SMC4*-*PK*_*3*_*-AID* + *SMC4*-*HA*_*3*_ cells were synchronized in G_1_ with α-factor and released into S phase in the presence of 0.05% MMS. Auxin was added for 60 min before cells were released for 45 min into S phase in the presence of MMS and EdU, after which MMS was removed to allow recovery for 30 min. EdU tracks lengths were measured after 45 min in MMS and after recovery. Distributions for three independent experiments are shown as box and whiskers plots (25th–75th and 10th–90th percentiles). Mean length is indicated in red. **p < 0.01; *p < 0.05; ns non-significant (unpaired *t*-test, two-sided). **c** WT and *SMC4*-*AID* cells were arrested in G_1_ with α-factor and auxin was added 60 min before release. Then, cells were released into S phase in the presence of 200 mM HU for 60 min. RPA enrichment at indicated distances from *ARS305* and *AR306* was measured by ChIP-qPCR and normalized to unreplicated regions. Mean +/- SD for three independent experiments are shown. Two-way ANOVA revealed a significant difference between WT and *SMC4*-*AID* cells. Post-hoc comparisons between WT and mutants at each position were adjusted for multiple testing (one family). *p < 0.05; **p < 0.01; ***p < 0.001; ns not significant. **d** Wild-type (WT), *rfa1-G77E*, *smc2-8*, and *rfa1-G77E smc2-8* cells were spotted on YPD plates containing 50 or 100 mM HU and were incubated for 4 days at the semi-permissive temperature of 30 °C. Source data are provided as a Source data file.

We next examined whether condensin promotes fork progression during replication stress. Fork dynamics was analyzed by DNA combing^29^ in HU-treated wild-type cells and *smc2-8* mutants. Cells were synchronized in G_1_ and released into S phase for 90 or 180 min at 35 °C in the presence of bromodeoxyuridine (BrdU) and 200 mM HU. BrdU tracks length increased by 8.7 kb between 90 and 180 min in control cells, but only by 4.4 kb in *smc2-8* mutants (Fig. 2a, Supplementary Fig. 2c and Supplementary Table 1). To rule out possible defects in S-phase entry, qPCR analysis of DNA copy number around early and late replication origins confirmed normal early origin firing and proper late origin repression in *smc2‑8* and Ycg1‑depleted cells (Supplementary Fig. 2b, d). Ycg1-depleted cells also exhibited significantly shorter BrdU tracks under HU (Supplementary Fig. 2e), even though condensin is dispensable for normal fork progression in the absence of drugs (Supplementary Fig. 2f). Together, these data indicate that condensin promotes fork progression under replication stress.

Because forks exposed to HU repeatedly arrest and restart as dNTP pools fluctuate, the reduced fork speed measured in *smc2-8* mutants may reflect a defect in fork restart. To test this, we evaluated fork recovery after DNA alkylation-induced fork stalling. Wild-type and *SMC4*-AID cells were arrested in G_1_ phase with α-factor and auxin was added one hour later to deplete Smc4. Following release from G_1_, cells were incubated for 45 min in the presence of 0.05% MMS and the thymidine analogue 5-ethynyl-2′-deoxyuridine (EdU). MMS exposure reduced DNA synthesis similarly in wild-type and Smc4-depleted cells (Fig. 2b, Supplementary Fig. 2g and Supplementary Table 1). Upon MMS removal, however, the mean length of EdU tracks was significantly shorter in Smc4-depleted cells than in wild type or Smc4-complemented cells (Fig. 2b, Supplementary Fig. 2g and Supplementary Table 1), indicating that condensin is required for efficient fork restart.

The DNA replication checkpoint is activated by RPA-coated single-stranded DNA (ssDNA) at stressed forks^30^. To determine the impact of condensin loss on ssDNA accumulation, we quantified RPA-bound ssDNA by ChIP-qPCR. WT and SMC4-AID cells were arrested in G_1_ with α-factor, auxin was added 60 min before release into S phase, and cells were treated with 200 mM HU for 60 min. RPA-bound ssDNA was then immunoprecipitated and quantified by qPCR^26^. In WT cells, ChIP-qPCR analysis revealed an RPA enrichment around *ARS305* and *ARS306*, consistent with increased RPA-coated ssDNA at HU-treated forks. Notably, RPA levels were significantly increased in condensin-depleted cells relative to controls (Fig. 2c and Supplementary Fig. 2h). A similar increase in RPA-bound ssDNA was also detected in *YCG1*-AID cells compared to control cells (Supplementary Fig. 2i), suggesting replication fork instability in the absence of condensin.

To test whether reducing RPA affinity for ssDNA could alleviate the increased sensitivity of condensin mutants to replication stress, we examined genetic interactions between condensin mutations and the *rfa1-G77E* allele, which weakens RPA-ssDNA binding^31^. While *smc2-8* cells grew poorly under HU-induced stress, the *rfa1-G77E* mutation partially restored their growth under normal conditions and fully suppressed HU sensitivity at semi-permissive temperature (Fig. 2d). Together, these results indicate that condensin promotes replication stress tolerance by limiting the accumulation or persistence of RPA-coated ssDNA at stalled replication forks.

### Topoisomerases cooperate with condensin to promote fork restart

**Fig. 3:**
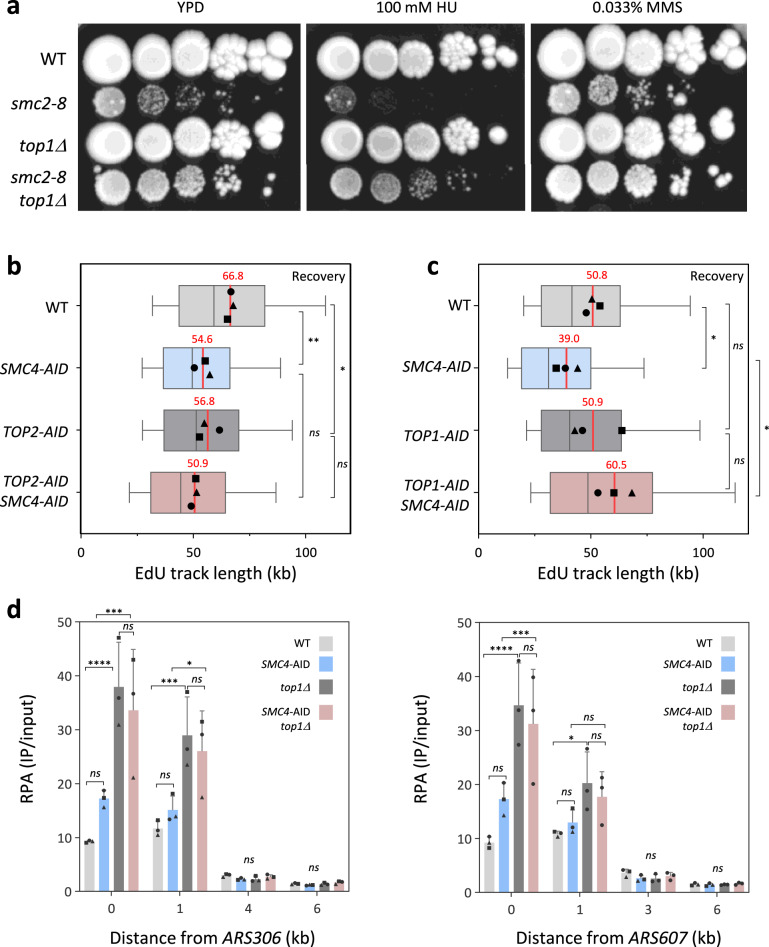
Topoisomerases cooperate with condensin to promote fork restart. **a** Deletion of *TOP1* partially suppresses the growth defect of a condensin mutant under replication stress. Wild-type (WT), *top1Δ*, thermosensitive *smc2-8*, and *top1Δ smc2-8* cells were grown for 6 days on YPD plates containing 0.033% MMS or 100 mM HU at the semi-permissive temperature of 30 °C. **b** Condensin acts with Top2 to promote fork restart after MMS exposure. Fork restart was analyzed by DNA combing in wild-type, *SMC4*-*PK*_*3*_*-AID*, *TOP2*-*MYC-AID* and *SMC4*-*PK*_*3*_*-AID*, *TOP2*-*MYC-AID* cells as indicated in Fig. 2b. The distribution of EdU track length is shown for three independent experiments. Mean length is indicated in red. **p < 0.01; *p < 0.05; ns non-significant (unpaired *t*-test, two-sided). **c** Top1 depletion restores fork restart in the absence of condensin. Fork restart after MMS exposure was monitored by DNA combing as in panel b in WT, *SMC4*-*PK*_*3*_*-AID*, *TOP1*-*AID* and *SMC4*-*PK*_*3*_*-AID*, *TOP1*-*AID* cells. The distribution of EdU tracks length is shown for three independent experiments. Mean length is indicated in red. *p < 0.05; ns non-significant (unpaired *t*-test, two-sided). **d** RPA-coated ssDNA accumulates at HU-treated forks in yeast cells lacking condensin and Top1. WT, *SMC4-AID*, *top1Δ* and *top1Δ SMC4*-*AID* cells were arrested in G_1_ with α-factor and SMC4 was depleted by addition of auxin for 60 min. Cells were then released into S phase in the presence of 200 mM HU for 60 min. RPA enrichment at indicated distances from *ARS306* and *ARS607* was measured by ChIP-qPCR and normalized to unreplicated regions. Mean +/− SD from three independent experiments are shown. Two-way ANOVA detected significant differences among conditions. Post-hoc comparisons between WT and mutants at each position were adjusted for multiple testing (one family). *p < 0.05; **p < 0.01; ***p < 0.001; ns not significant. Source data are provided as a Source data file.

Several lines of evidence suggest that the role of condensin at stalled replication forks may depend on DNA topoisomerases. Condensin is functionally coupled to both topoisomerases I and II, and condensin-mediated DNA supercoiling has been proposed to facilitate chromosome decatenation by topoisomerase II^32–35^. Topoisomerases also play essential roles in replication fork progression by relieving positive superhelical tension ahead of the fork^36^. To investigate the functional interplay between condensin and topoisomerases during replication stress, we compared the growth of *smc2-8, top1Δ*, *top2-4*, *smc2-8 top1Δ* and *smc2-8 top2-4* cells in the presence of HU or MMS. At 30 °C, a semi-permissive temperature for the *smc2-8* allele, the *smc2-8 top1Δ* double mutant consistently displayed improved growth relative to *smc2-8* cells under all tested conditions (Fig. 3a). In contrast, no rescue was observed for the *smc2-8 top2-4* double mutant (Supplementary Fig. 3a).

To further assess the functional interaction between condensin and topoisomerases, we used auxin-inducible degron (AID) alleles to enable acute depletion of topoisomerases. We first confirmed that AID tagging did not interfere with S-phase progression in cells expressing Smc4-AID, Top2-AID, or Top1-AID. To this end, cells were synchronized in G_1_ and released into S phase in the presence or absence of auxin. Flow cytometry analysis of DNA content revealed no cell cycle defect, and western blot analysis confirmed efficient degradation of the tagged proteins (Supplementary Fig. 3b, c). Furthermore, analysis of replication fork progression by DNA combing in asynchronous cells after 60 min of auxin treatment followed by a 15 min EdU pulse further showed that AID tagging did not alter replication dynamics (Supplementary Fig. 3d, e).

We next examined the ability of cells to restart MMS-arrested replication forks upon depletion of topoisomerase I, topoisomerase II, and/or condensin. Wild-type, *SMC4*-AID, *TOP2*-AID, *TOP1*-AID, *SMC4*-AID *TOP2*-AID, and *SMC4*-AID *TOP1*-AID cells were arrested in G_1_ phase with α-factor, and auxin was added 1 h later to induce degradation of the AID-tagged proteins (Supplementary Fig. 3f, g). α-factor was then removed to allow the cells to enter S phase in the presence of EdU and 0.05% MMS, and the length of EdU tracks was measured by DNA combing before or after release from MMS. No significant differences in EdU track length during the MMS treatment were observed in any depleted cells when compared with wild-type cells (Fig. 3b, c and Supplementary Fig. 3h, i). By contrast, EdU tracks measured after recovery from MMS were shorter in *SMC4*-AID and *TOP2*-AID cells relative to controls, but not in *TOP1*-AID cells (Fig. 3b, c, Supplementary Fig. 3h, i and Supplementary Table 1).

Strikingly, simultaneous depletion of Smc4 and Top1 restored fork restart to wild type levels, indicating that condensin is required for efficient fork restart in the presence of Top1, but dispensable when Top1 is absent (Fig. 3c and Supplementary Fig. 3i). In contrast, simultaneous depletion of Smc4 and Top2 did not further impair fork recovery relative to depletion of either protein alone, suggesting that condensin and Top2 act in a common pathway required for efficient fork restart following replication stress (Fig. 3b and Supplementary Fig. 3h).

Finally, we monitored RPA-coated ssDNA at HU-treated forks in wild-type, *SMC4-*AID, *top1Δ*, and *top1Δ SMC4*-AID cells released from G_1_ into S phase for 60 min in 200 mM HU (Fig. 3d and Supplementary Fig. 3j). As shown in Supplementary Fig. 2h, RPA levels increased in the absence of condensin, and were even more pronounced in *top1Δ* cells and in *top1Δ SMC4-AID* double mutant. Together, these data reveal a complex interplay between condensin, Top1 and Top2 at stalled forks, which is essential to prevent excessive RPA accumulation and to promote efficient fork restart.

### Condensin II travels with replication forks in human cells and promotes fork restart

**Fig. 4:**
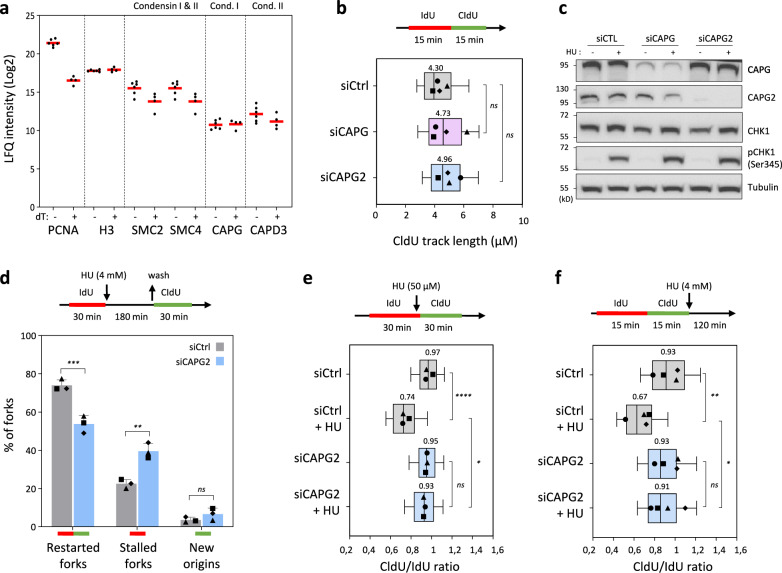
Condensin II promotes fork processing and restart in human cells. **a** HeLa-S3 cells were labeled for 20 min with 10 mM EdU and chased for 60 min with thymidine. Proteins bound to nascent DNA were analyzed by iPOND-MS^79^. Dots correspond to biological replicates. LFQ, label-free quantification. **b** HeLa-S3 cells depleted of condensin I (siCAPG) or condensin II (siCAPG2) for 48 h with siRNAs were labeled with IdU and CldU for 15 min each. CldU track length from four independent experiments is shown. **c** HeLa-S3 cells were transfected with siCtrl, siCAPG and siCAPG2 for 48 h and then treated with 4 mM HU for 2 h. CHK1 activation was detected by immunoblotting with an anti-pCHK1 (S345). CAPG and CAPG2 depletion was verified by Western blotting. **d** HeLa-S3 cells were transfected with siCtrl or siCAPG2 for 48 h, pulsed with IdU for 30 min, treated with 4 mM HU for 3 h and then labeled with CldU after HU washout. IdU and CldU tracks were analyzed. Red-green tracks: fork restart. Red tracks: stalled forks. Green tracks: new origin (*n* = 3). Two-way ANOVA and post hoc comparisons between siCtrl and siCAPG2 for each fork type were adjusted for multiple testing (one family). **p < 0.01; ***p < 0.001; ns not significant. **e** U2OS cells were transfected with siCtrl and siCAPG2 for 48 h, labeled with IdU for 30 min, and then with CldU for 30 min in the presence of 50 mM HU. IdU and CldU track lengths were measured by DNA fiber spreading and plotted as the ratio of CldU to IdU (*n* = 3). **f** HeLa-S3 cells were transfected with siCtrl and siCAPG2 for 48 h and sequentially labeled with IdU and CldU for 15 min each. Cells were either collected immediately or treated with 4 mM HU for 2 h before DNA fiber analysis. The ratio of CldU to IdU track length from four independent experiments is plotted. All box and whiskers plots indicate median, 25th–75th and 10th–90th percentiles. ****p < 0.0001; *p < 0.05; ns non-significant (unpaired *t*-test, two-sided). Source data are provided as a Source data file.

To further characterize the role of condensin in response to replication stress, we next asked whether it also associates with replication forks in human cells. To this end, we labeled HeLa S3 cells for 20 min with 10 µM EdU under normal growth conditions and analyzed the proteins associated with nascent DNA by using iPOND (isolation of proteins on nascent DNA) coupled with mass spectrometry (iPOND-MS)^37^. Smc2 and Smc4, two SMC subunits common to condensin I and II complexes, were associated with nascent DNA (Fig. 4a), consistent with previous reports^18,37^. Their abundance at forks decreased upon thymidine chase, similar to PCNA, which served as a positive control; whereas histone H3 levels remained unchanged. Notably, the condensin II subunit CAPD3 also showed modest enrichment at replication forks, whereas the condensin I subunit CAPG did not, suggesting that condensin II, rather than condensin I, associates with and travels with replication forks.

To test whether condensin participates in DNA synthesis, we depleted the condensin I subunit CAPG and the condensin II subunit CAPG2 from HeLa S3 cells using siRNAs and monitored replication fork progression. Exponentially growing cells were sequentially pulse-labeled with the thymidine analogues iododeoxyuridine (IdU) and chlorodeoxyuridine (CldU) for 15 min each, and individual DNA molecules were stretched on glass slides by DNA fiber spreading, followed by immunodetection of IdU and CldU tracks, as described previously^38,39^. Forks progressed at similar rates in all cell lines (Fig. 4b, c and Supplementary Fig. 4a), indicating that condensin I and II are dispensable for normal fork progression in the absence of exogenous replication stress, consistent with our observations in budding yeast.

To determine whether condensin promotes fork restart in human cells, as observed in budding yeast, we used DNA fiber spreading to quantify the percentage of restarted forks, stalled forks and newly activated origins after transient exposure to HU. HeLa S3 cells were transfected for 48 h with siRNAs against CAPG2 or with control siRNAs (siCtrl). Cells were labeled with IdU for 30 min and grown for 180 min in the presence of 4 mM HU. Then, HU was removed and the cells were labeled for a further 30 min with CldU and subjected to DNA fiber spreading (Fig. 4d). Strikingly, CAPG2-depleted cells exhibited a ~2-fold increase in the frequency of stalled forks and a significant decrease in fork restart, accompanied by an ~2-fold increase in new origin firing relative to control cells (Fig. 4d). In contrast, U2OS cells transfected for 48 h with siRNAs against CAPG or with control siRNAs (siCtrl), and treated in the same manner, showed no change in fork restart efficiency (Supplementary Fig. 4b). Together, these results indicate that condensin II, but not condensin I, promotes replication fork restart in human cells.

Our data suggest that condensin II is dispensable for normal fork progression but important for processing stalled forks, mirroring its role in budding yeast. Human cells respond to various replication stressors by ATP-dependent fork reversal, which slows replication and facilitates the resection of nascent DNA^40^. To determine whether condensin II is required for this slowing and resection, we first labeled CAPG2-depleted cells and control cells with IdU followed by CldU for 30 min in the presence of 50 µM HU and analyzed replication dynamics by DNA fiber spreading. In control cells, the CldU/IdU ratio decreased by 23% upon HU treatment (Fig. 4e and Supplementary Fig. 4c), consistent with fork slowing under conditions that do not significantly deplete dNTP pools but instead induce fork reversal^3,41^. In CAPG2-depleted cells, replication fork speed was unchanged under the same conditions (Fig. 4e and Supplementary Fig. 4c), indicating that condensin II is required for HU-induced fork slowing.

To assess whether condensin II is required for fork resection, we labeled HeLa cells depleted of CAPG or CAPG2, as well as control cells, with IdU followed with CldU and then cultured them for an additional 120 min in the presence or absence of 4 mM HU. DNA fibers were stretched on glass slides, the length of IdU and CldU tracks was measured for individual fork to calculate the CldU/IdU ratio. In the absence of HU, this ratio was close to 1 for all cell types, whereas it was reduced by 26% in HU-treated control cells (Fig. 4f and Supplementary Fig. 4d), consistent with limited MRE11-dependent resection reported in various BRCA -proficient cell lines^38,39^. This controlled resection is distinct from the excessive degradation seen in HU-treated, BRCA-deficient cells^42^. Remarkably, CAPG2-deficient HeLa cells failed to undergo resection of nascent DNA under these conditions (Fig. 4f and Supplementary Fig. 4d), and similar results were obtained in U2OS cells (Supplementary Fig. 4e), indicating that condensin II is required for this process. Consistently, acute degradation of SMC4 in Smc4-mAID-Halo cells using 5-PhIAA for 2.5 h^43^ similarly impaired fork resection (Supplementary Fig. 4f), whereas depletion of the condensin I subunit CAPG did not affect fork resection (Supplementary Fig. 4d). Together, these results indicate that condensin II, but not condensin I, is essential for nascent DNA resection at HU-treated forks in human cells.

To determine whether condensin II is required for human cell growth under replication stress, we monitored the growth of control and CAPG2-depleted HeLa S3 cells over 3 days in the presence of increasing HU concentrations (0, 100, and 300 µM). CAPG2-depleted cells displayed a dose-dependent growth defect, with proliferation reduced by approximately 8% in the absence of HU and up to 58% at 300 µM HU compared with control cells (Supplementary Fig. 4g). In line with this, CAPG2 depletion reduced colony formation by up to 50% following exposure to 1- or 2-mM HU (Supplementary Fig. 4h), indicating that condensin II promotes survival after replication stress. Despite this sensitivity, CAPG2-depleted cells efficiently activated the DNA replication checkpoint, as evidenced by CHK1 phosphorylation at Ser345 (Fig. 4c). Together, these data indicate that condensin II supports human cell proliferation under replication stress in a manner that does not depend on checkpoint activation.

### Condensin II and SMARCAL1 promote fork restart by different mechanisms

**Fig. 5:**
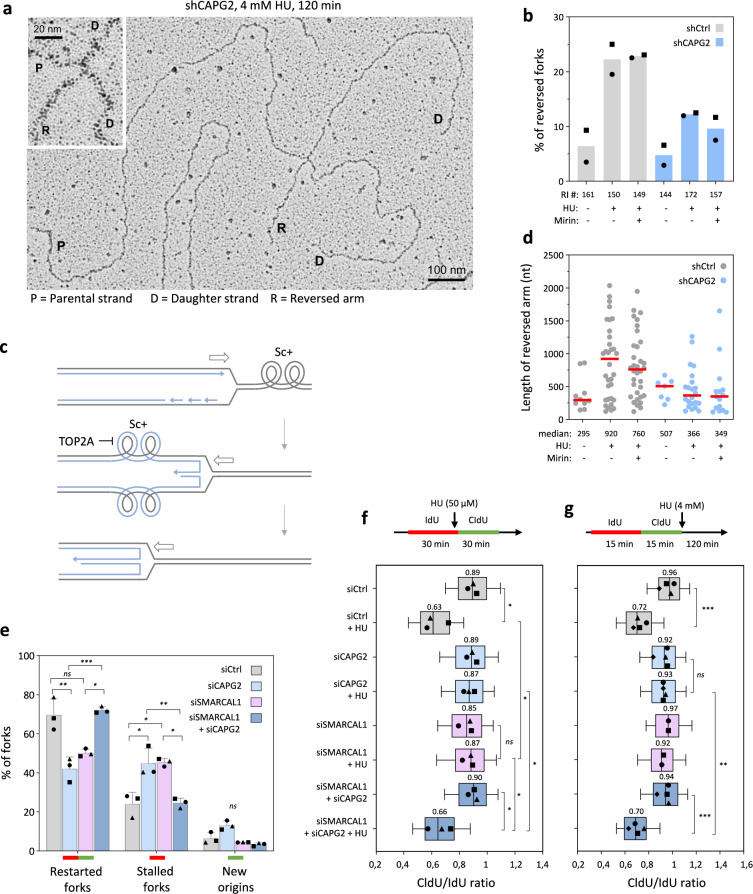
Condensin II promotes fork reversal. **a** Electron microscopy (EM) analysis of the frequency of reversed forks in control (shCtrl) and CAPG2- depleted (shCAPG2) HeLa-S3 cells treated for 72 h with 10 µg/ml doxycycline and then for 2 h with 4 mM HU with or without 50 µM mirin. Representative electron micrographs of replication intermediates from shCAPG2 cells are shown. **b** Quantification of reversed forks in shCtrl and shCAPG2 cells under the indicated conditions. Mean frequencies and the number of analyzed forks are indicated (*n* = 2). “RI #“: number of replication intermediates scored. **c** Schematic model of the fork reversal mechanism. See main text for details. **d** The length of reversed arms (nt) is plotted for the indicated samples, with median values shown (*n* = 2). “RI #” indicates the number of replication intermediates analyzed. **e** HeLa-S3 cells were transfected for 48 h with siCtrl, siSMARCAL1, siCAPG2 or both siSMARCAL1 and siCAPG2. Cells were pulsed with IdU for 30 min, treated with 4 mM HU for 3 h and fork restart was monitored 30 min after HU removal and CldU addition, as in Fig. 3d (*n* = 3). Two-way ANOVA detected significant differences among categories. Post-hoc comparisons between siCtrl, siCAPG2, siSMARCAL1 and siCAPG2 + siSMARCAL1 for each fork type were adjusted for multiple testing (one family). **p < 0.01; *p < 0.05; ***p < 0.001; ns not significant. **f** U2OS cells were transfected with siCtrl, siSMARCAL1, siCAPG2 or both siSMARCAL1 and siCAPG2 for 48 h. Cells were labeled for 30 min with IdU and 30 min with CldU in the presence of 50 mM HU and processed for DNA fiber spreading. The ratio of CldU to IdU track length from three independent experiments is shown. **g** HeLa-S3 cells were transfected as in panel f, sequentially labeled with IdU and CldU for 15 min each, and either collected immediately or treated for 2 h with 4 mM HU before DNA fiber analysis. The ratio of CldU to IdU track length from four independent experiments was plotted as box and whiskers, as indicated for Fig.4 (unpaired *t*-test, two-sided). Source data are provided as a Source data file.

Our findings indicate that condensin II promotes fork slowing in the presence of 50 µM HU and is essential for nascent DNA resection. Since both processes depend on fork reversal^3^, we next asked whether condensin II plays a direct role in this process by exposing control and shCAPG2-depleted cells to 4 mM HU (Supplementary Fig. 5a, b). Replication intermediates were analyzed by electron microscopy after psoralen crosslinking, with or without the MRE11 inhibitor mirin to block reversed fork degradation^44^. Notably, CAPG2-depleted cells exhibited 2–3-fold fewer reversed forks than HU-treated control cells, where reversed forks comprised ~23% of replication intermediates (Fig. 5a, b and Supplementary Table 2) consistent with previous studies^45,46^. These data indicate that condensin II facilitates fork reversal.

Replication fork reversal occurs in two steps. First, SMARCAL1, together with RAD51 and other factors, initiates limited replication fork reversal, generating positive superhelical strain in the newly replicated sister chromatids. TOP2A then resolves this topological strain to enable extensive fork reversal (Fig. 5c)^7,47^. In CAPG2-depleted cells, not only were reversed forks less frequent, but reversed arm lengths were also two-fold shorter than in controls (Fig. 5d), a phenotype reminiscent of TOP2A-depleted cells^7^ and consistent with condensin II promoting the extension phase of fork reversal.

To define how condensin II and SMARCAL1 process stalled forks, we analyzed fork restart by DNA fiber spreading in cells lacking CAPG2, SMARCAL1, or both. Depletion of either protein alone significantly increased stalled forks and decreased fork restart (Fig. 5e), supporting independent contributions to fork restart. Strikingly, co-depletion of both restored stalled and restarted forks proportions to near control levels, suggesting cells engage an alternative restart mechanism when both factors are absent.

To further dissect the roles of SMARCAL1 and condensin II in fork reversal, we analyzed fork slowing and resection in cells lacking CAPG2, SMARCAL1, or both (Fig. 5f, g and Supplementary Fig. 5c–f). Like CAPG2-depleted cells, SMARCAL1-depleted cells failed to slow forks (Fig. 5f and Supplementary 5c) or resect nascent DNA under HU treatment (Fig. 5g and Supplementary 5e), consistent with both promoting fork reversal. Remarkably, CAPG2 depletion restored both fork slowing and resection in SMARCAL1-depleted cells (Fig. 5f, g and Supplementary Fig. 5c–f). These results suggest that SMARCAL1 and condensin II are mutually dispensable for fork reversal, revealing a coordinated or compensatory relationship.

Two RAD51-dependent fork reversal pathways exist: one driven by SMARCAL1 and other DNA translocases and another by the F-box DNA helicase FBH1^48^. CAPG2 co-depletion similarly restored resection in FBH1-deficient cells and in cells lacking both SMARCAL1 and FBH1 (Supplementary Fig. 5g). Together, these data suggest that while condensin II promotes fork reversal extension together with TOP2A, it acts as a physical barrier to fork reversal - and thus resection - in the absence of SMARCAL1 or FBH1.

### Condensin II acts with TOP1 and TOP2A to promote fork slowing and resection

**Fig. 6:**
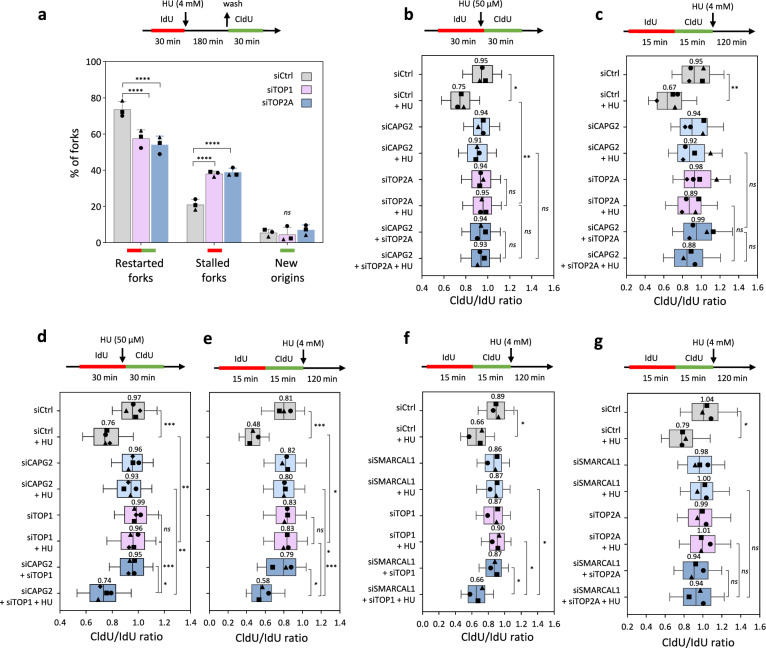
TOP1 and TOP2A play different roles in fork slowing and resection. **a** HeLa-S3 cells were transfected with siCtrl, siTOP1 and siTOP2A for 48 h and were treated as described in Fig. 4d. **b** U2OS cells were transfected for 48 h with siCtrl, siCAPG2, siTOP2A, or both siTOP2A and siCAPG2. Cells were labeled with IdU for 30 min and with CldU for 30 min in the presence of 50 mM HU, then processed for DNA fiber analysis. **c** HeLa-S3 cells were transfected with siCtrl, siCAPG2, siTOP2A, or both siTOP2A and siCAPG2 for 48 h. Cells were sequentially labeled with IdU and CldU for 15 min each and either collected immediately or treated for 2 h with 4 mM HU before DNA fiber analysis. The ratio of CldU to IdU track lengths from four independent experiments was plotted as box and whiskers. **d** U2OS cells were transfected with siCtrl, siCAPG2, siTOP1, or both siTOP1 and siCAPG2 for 48 h. Cells were labeled and analyzed by DNA fiber spreading as in panel (**b**) (*n* = 4). **e** TOP1 depletion restores fork resection in CAPG2-deficient cells. HeLa-S3 cells were transfected with siCtrl, siCAPG2, siTOP1, or both siTOP1 and siCAPG2 for 48 h. Cells were labeled and analyzed by DNA fiber spreading as indicated in panel (**c**) (*n* = 3). **f** HeLa-S3 cells were transfected with siCtrl, siSMARCAL1, siTOP1, or both siSMARCAL1 and siTOP1 for 48 h. Cells were labeled and analyzed by DNA fiber spreading as in panel (**c**) (*n* = 3). **g** HeLa-S3 cells were transfected with siCtrl, siSMARCAL1, siTOP2A, or both siSMARCAL1 and siTOP2A for 48 h. Cells were labeled and analyzed by DNA fiber spreading as in panel (**c**) (*n* = 3). All box and whiskers plots indicate median, 25th–75th and 10th–90th percentiles. ****p < 0.0001; *p < 0.05; ns non-significant (unpaired *t*-test, two-sided). Source data are provided as a Source data file.

To determine whether TOP1 and TOP2A also promote fork restart in human cells, as observed for condensin II (Fig. 4d), we performed DNA fiber assays in U2OS cells depleted of TOP1, TOP2A, or treated with control siRNAs (siCtrl). Cells were transfected for 48 h with siRNAs, pulse-labeled with IdU (30 min), exposed to 4 mM HU for 180 min, and then labeled with CldU for 30 min after HU washout (Fig. 6a). TOP1- and TOP2A-depleted cells both exhibited increased stalled forks, reduced fork restart and increased origin firing relative to controls, indicating that both TOP1 and TOP2A promote fork restart in HeLa cells.

Since TOP2A resolves positive torsional stress during fork reversal^6,7^, we examined whether condensin II collaborates with TOP2A by assessing fork slowdown and fork resection as proxies for reversal in cells lacking CAPG2, TOP2A or both. TOP2A-depleted cells were defective in both processes (Fig. 6b, c and Supplementary Fig. 6a–d), consistent with its established role in fork reversal^7^ and mirroring the phenotype of condensin II-depleted cells. In contrast, TOP2B - which organizes chromatin loops and TADs^8^ - was dispensable for nascent DNA resection (Supplementary Fig. 6e, f). Co-depletion of TOP2A and CAPG2 did not exacerbate the defects, suggesting these factors function in the same pathway.

Given that condensin activity depends on TOP1 in vitro^49,50^, we next examined the role of TOP1 in fork slowing and resection using cells lacking CAPG2, TOP1 or both. Both processes were similarly impaired in cells depleted of either TOP1 or CAPG2 alone, yet co-depletion restored fork slowing and resection to control levels (Fig. 6d, e and Supplementary Fig. 6g–j). This synthetic rescue contrasts with the TOP2A/CAPG2 epistasis, indicating that TOP1 and TOP2A play distinct roles at stalled forks.

Because TOP1 relaxes negative supercoils and condensin restricts RPA binding to ssDNA^51–53^, we quantified RPA accumulation on chromatin by immunofluorescence in cells depleted of CAPG2, TOP1, TOP2A, or their combinations. Following HU treatment, RPA foci increased 2.4-fold in CAPG2-depleted cells relative to controls, and more modestly in siTOP1, siTOP2A or siCAPG2 + siTOP2A cells (Supplementary Fig. 6k). In contrast, co-depletion of TOP1 and CAPG2 further elevated chromatin-bound RPA to 4.8-fold, a phenotype reminiscent of yeast *SMC4-AID top1Δ* cells (Fig. 3d). Although the underlying source of this excess ssDNA remains currently unclear, these data reinforce the idea that TOP1 and TOP2A have distinct, non-redundant functions in the replication stress response mediated by condensin II.

**Fig. 7:**
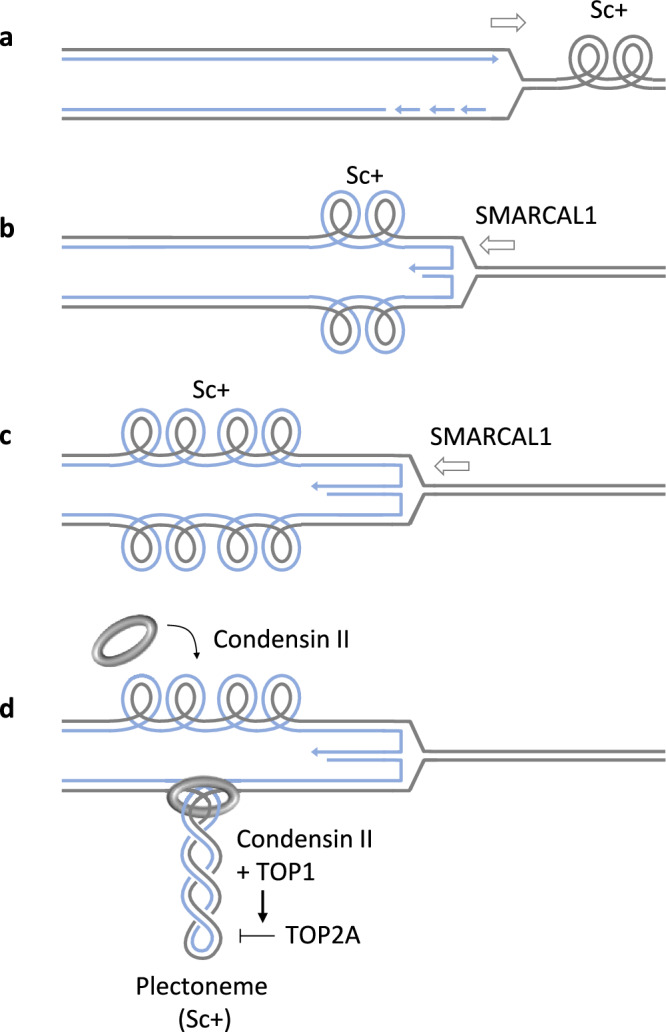
Model of the roles of condensin and topoisomerases in fork reversal in human cells. **a** During replication elongation, unwinding of the parental duplex generates positive supercoils (Sc+) ahead of replication forks, which are normally relaxed by TOP1. Fork rotation also produces precatenanes behind the fork, which are resolved by TOP2. **b** When a replication fork stalls and reverses, Sc+ accumulates behind the fork owing to the unwinding of parental and nascent strands, creating a topological barrier that limits the extent of fork reversal. **c** Further fork reversal is driven by DNA translocases such as SMARCAL1, which generate additional Sc+ along newly replicated sister chromatids. This slows replication and produces a one-ended DNA arm that is vulnerable to nucleolytic degradation. **d** Condensin II binds Sc+ and, together with TOP1, converts dispersed Sc+ into positively supercoiled plectonemes that facilitate TOP2-mediated relaxation and limit the formation of harmful catenanes and knots between sister chromatids. In the absence of condensin II or TOP1, these supercoiled loops do not form, and TOP2 alone can drive fork reversal in a SMARCAL1‑independent manner, but at the cost of increased defects in fork restart. Whether a similar mechanism operates in budding yeast remains unclear. Indeed, reversed arm extension is more limited than in human cells, and alternative pathways for parental DNA reannealing may be equally important. Further work is needed to elucidate how condensin and topoisomerases cooperate to promote fork restart in this unicellular organism.

We next investigated functional interactions between SMARCAL1 and topoisomerases at HU-treated forks. As in CAPG2-depleted cells, SMARCAL1 was dispensable for fork slowing and resection in TOP1-deficient cells (Fig. 6f and Supplementary Fig. 6l, m). However, SMARCAL1 remained essential for resection in TOP2A-deficient cells, consistent with previous findings that TOP2A and SMARCAL1 act in the same pathway^7^ (Fig. 6g and Supplementary Fig. 6n). Together, these results suggest that SMARCAL1 becomes dispensable for reversal and resection when condensin II or TOP1 are absent, but remains required in the absence of TOP2A. This pattern supports a model in which condensin II and TOP1 organize positively supercoiled DNA into plectonemic structures at reversed forks, which TOP2A subsequentially relaxes to extend the regressed arm (Fig. 7).

## Discussion

In this study, we investigated the role of condensin in S phase and, in particular, whether condensin and topoisomerases cooperate to relieve positive DNA supercoiling at replication stress sites. In budding yeast, we show that condensin is recruited to stalled forks in by the MRX complex, where it cooperates with the topoisomerases Top1 and Top2 to promote fork restart. In human cells, we further find that condensin II is specifically required for resection of nascent DNA at stalled forks, thereby enabling replication restart. Condensin II and SMARCAL1 carry out distinct functions to promote fork restart in human cells. In addition, human condensin II cooperates with TOP1 and TOP2A to promote fork reversal. Together, our findings support a model in which condensin II and TOP1 act at reversed forks to convert positive supercoils into higher-order topological DNA structures that are subsequently relaxed by TOP2A, allowing replication to resume.

### Condensin is recruited to replication forks in budding yeast and human cells

In budding yeast, condensin has been reported to bind multiple loci during interphase, including replication origins and transcription sites^12^. Here, we found that condensin is enriched specifically around active early replicating origins, but not at repressed late origins in HU-treated cells, indicating that it binds origins after initiation. This is reminiscent of condensin recruitment to centromeric regions, which also depends on DNA replication^14^. In HU-treated *S. cerevisiae* cells, replication forks progress very slowly (0.1 kb per minute)^23^, allowing us to track condensin in time-resolved ChIP-seq experiments. We observed that condensin moves away from origins with kinetics similar to DNA replication, suggesting that it follows stressed replication forks. In human cells, iPOND analysis showed that condensin associates with newly replicated DNA in the absence of HU^54^, consistent with previous reports^55^ and indicating that active forks can also recruit condensin.

Our findings raise the question of how condensin is recruited to replication sites. Condensin is recruited to transcriptionally active chromatin regions by transcription factors and nucleosome displacement^56–59^, although the underlying mechanism remains unclear. In HU-treated yeast cells, we found that MRX-dependent remodeling of nascent chromatin is a key determinant of condensin recruitment to replication sites, as previously described for cohesin^26^. Our data do not exclude the possibility that condensin is loaded elsewhere in the genome and ten slides along DNA until it encounters barriers such as tightly bound protein complexes^60–62^, closed topological domains^5,6^ or entangled DNA structures^34,63^. An important open question is whether condensin complexes recruited to replication pause sites in S phase also contribute to chromosome condensation in G_2_/M.

### A role for condensin in fork repair

We show that loss of condensin in budding yeast and human cells reduces cell growth and survival under replication stress. This is consistent with earlier studies reporting increased DNA damage sensitivity in condensin mutants across several species^17–22,27^. Collectively, these observations suggest that condensin either has a direct role in DNA repair during interphase or that DNA damage secondarily exacerbates chromosome segregation defects, which are frequent in condensin-deficient cells. To distinguish between these possibilities, we depleted condensin in G_1_-arrested yeast cells and used DNA combing to monitor fork progression, arrest and restart in the subsequent S phase. This analysis revealed that condensin is required for timely recovery of stressed forks after HU or MMS treatment, independently of its canonical role in chromosome segregation and independently of S-phase checkpoint activation, in contrast to fission yeast^27^. Condensin II depletion in human cells similarly impaired fork restart without altering checkpoint activation, supporting a direct role for condensin in fork repair.

### Condensin acts with TOP2A to promote fork reversal in human cells

Using three independent experimental approaches, we demonstrate that condensin promotes fork reversal in human cells. Depletion of the condensin II subunit CAPG2 reduced both fork resection and fork slowdown in HU-treated cells, two hallmarks of fork reversal^3^. Electron microscopy of replication intermediates confirmed that fork reversal is impaired upon condensin II depletion, and also revealed that the remaining regressed arms are shorter than in controls, suggesting a role for condensin II in the extension phase of fork reversal. Indeed, fork reversal is a two-step process involving formation of a short, unstable reversed arm followed by its extension in a process involving TOP2A, the SUMO E3 ligase ZATT, and a SUMO-targeted DNA translocase, PICH^3,7^. TOP2A promotes the extension of reversed forks by relaxing positive DNA supercoiling generated when SMARCAL1 and other translocases unwind sister chromatid DNA. Consistent with condensin II and TOP2A act in the same pathway, co-depletion of CAPG2 and TOP2A did not further impede fork resection or slowdown.

This collaboration between condensin and TOP2A mirrors their functional interplay in M phase^9^. In G_2_/M, TOP2A catalyzes both catenation and decatenation of sister chromatids, and condensin promotes chromosome segregation by shifting the balance towards decatenation^33,34,64–66^. Inactivation of condensin in metaphase leads to de novo formation of sister chromatid intertwines^67,68^, indicating that condensin promotes TOP2A-mediated unlinking while close chromatid proximity favors interlinking. The underlying mechanism, however, remains debated. Condensin may stimulate TOP2 decatenation either by inducing positive supercoiling^32,67^, by constraining intra- and inter-molecular DNA contacts without altering DNA supercoiling^34,69^, or by binding positively supercoiled plectonemes and extruding them into a stable supercoiled loop to prevent knots and intertwines^70^. By analogy, we propose that during fork reversal, condensin II sequesters positive superhelical DNA into large plectonemes, thereby facilitating TOP2A-mediated relaxation and preventing the formation of harmful topological structures (Fig. 7). Therefore, condensin likely uses similar molecular activities in mitosis and S phase to relieve torsional stress by promoting Top2 function. In mitosis, this activity supports chromosome condensation and segregation^14,49^, whereas in S phase it resolves torsional stress generated by fork reversal. The distinction is therefore contextual rather than mechanistic, emphasizing a non-mitotic role for condensin.

### Condensin II and SMARCAL1 cooperate to promote fork reversal

It has been proposed that TOP2A, ZATT, and PICH promote fork reversal by relaxing positive supercoiling generated by SMARCAL1 and other translocases^7^. Our data are consistent with this model, as depletion of either TOP2A, SMARCAL1 or both similarly prevented fork slowdown and resection. We further found that simultaneous depletion of condensin II and SMARCAL1 restored fork slowdown and resection in HU-treated cells, indicating that SMARCAL1 becomes dispensable for fork reversal when condensin II is absent. This is unexpected, given the prevailing view that SMARCAL1 is essential for fork reversal in many contexts^3^. One possible explanation is that the plectonemic structures generated by condensin during fork reversal act as a physical barrier to extension of the reversed arm. In the absence of this barrier, SMARCAL1’s activity may no longer be required or may be compensated by other factors, such as RAD51^3,71^.

### TOP1 and TOP2A promote fork reversal by different mechanisms

TOP1 resolves both negative and positive superhelical tension^8^. Like TOP2A and CAPG2, TOP1 is essential for fork slowing and reversal. However, unlike TOP2A, TOP1 depletion rescues fork defects in SMARCAL1-depleted cells, indicating that TOP1 and TOP2A promote fork reversal through distinct mechanisms.

It has been proposed that TOP1 promotes productive condensin activity through its participation in a “pinch-and-merge” mechanism of condensin-mediated loop extrusion. During this process, condensin generates torsional stress and local supercoiling as it pinches off and merges DNA loops. TOP1 relieves the negative supercoiling generated during these steps via transient single-strand cleavage and controlled DNA rotation, thereby allowing condensin to continue its activity^35,49,72^.

This model suggests that TOP1 may also support condensin function at reversed forks. However, co-depletion of TOP1 and CAPG2 rescued the fork defects seen upon depletion of either factor alone, indicating that TOP1 also contributes to replication stress responses independently of condensin II. This contrasts with TOP2A, which acts in the same pathway as condensin II to promote for fork reversal. Interestingly, in budding yeast and human cells lacking condensin and TOP1, HU treatment induced accumulation of RPA-coated ssDNA, consistent with a role for these factors in parental DNA strand reannealing, as previously proposed for replication of centromeric DNA in *Xenopus* egg extracts^52^. Interestingly, weakening RPA-ssDNA binding with the *rfa1-G77E* allele^31^ suppressed the HU sensitivity of the *smc2-8* mutant, supporting the view that excess RPA-coated ssDNA is toxic, presumably by preventing the reannealing of parental DNA strands during fork restart. However, we cannot exclude the possibility that this excess ssDNA arises independently of fork reversal and further work is required to clarify its origin. Nevertheless, our genetic data show that reducing RPA affinity for ssDNA alleviates the HU hypersensitivity of condensin mutants, which reinforces the idea that condensin also prevents the accumulation of excess ssDNA under replication stress, in addition to its role in fork reversal.

Taken together, our findings support a model in which condensin collaborates with DNA topoisomerases to relieve topological constraints generated during fork reversal while preventing the formation of harmful knots and sister chromatid intertwines by TOP2A. They raise important questions about the precise topological structures generated by condensin II at reversed forks, and about the role of TOP1 in this process. Because other SMC complexes (cohesin, MRN/X, and SMC5,6) are also recruited to stalled forks under replication stress^26,73,74^, it will be important to decipher how condensin is coordinated with these complexes during fork reversal. In budding yeast, both cohesin and condensin depend on MRX for recruitment to HU-treated forks^26^. Given that cohesin and condensin cooperate during mitosis to ensure accurate chromosome segregation, they may likewise collaborate at reversed forks. While condensin promotes fork restart in both yeast and human cells, underlying mechanisms likely differ, as template switching and other recombinational repair pathways are more prominent in budding yeast^75,76^. Finally, it remains unclear whether these condensin-dependent processes operate during unperturbed S phase and, if so, whether they contribute to interphase condensin loading for mitotic chromosome condensation and segregation.

## Methods

### Standard yeast genetics

All strains used were listed in Supplementary Table 3. All strains used were haploid and derived from W303. For the cell sensitivity to genotoxic drugs, cells were adjusted to 1 × 10^7^ cells/mL and serial dilution (1:10) were spotted on plates with HU (Sigma, H8627) or MMS (Sigma, 129925). Of note, the *smc2-8* mutation corresponds to the insertion of four perfect repeats and two imperfect repeats of the sequence MEQKLISEEDLNE, which corresponds to the canonical Myc epitope sequence EQKLISEEDL (10 amino acids) with additional flanking sequences. This sequence is inserted close to the hinge region of Smc2, interfering with the function of the condensin complex (Manuel Mendoza, IGBMC, personal communication).

### Cell growth and synchronization

Cells were grown at 25 °C in YPD or in YN- without methionine, supplemented with 2% glucose. 6 × 10^6^ exponentially growing cells were synchronized in G_1_ using 6 μg/mL of α-factor (Biotem, 2968) during 170 min. G_1_ arrested cells were released into S phase by filtration and resuspension in fresh medium or by the addition of 75 μg/mL Pronase (Sigma, 53702). Cells were treated or not with 200 mM HU or 0.05% MMS, as indicated. For degron strains, 1 mM auxin (IAA) was added to the liquid culture after 120 min of synchronization in G_1_ and every hour after release in S phase.

### Human cell culture

Human cervical adenocarcinoma HeLa S3 cells (ATCC, CCL2-2) and osteocarcinoma U2OS cells (ATCC, HTB-86) were cultured in Dulbecco’s modified Eagle’s medium (DMEM) or McCoy’s 5a medium, respectively, supplemented with 10% fetal calf serum (FCS) and 100 U/mL penicillin/streptomycin (PS). HeLa-S3 shCtrl and shCAPG2 were cultured in DMEM supplemented with 10% tetracyclin-free FCS and 100 U/mL PS. All cells were cultured at 37 °C in a 5% CO2 incubator.

### Production of lentiviral vectors and transduction

HIV-1-derived lentiviral vectors were produced in HEK293T cells as previously described^77^. Cells were seeded on poly-D-lysine coated plates and transfected with packaging plasmid (psPAX2, Addgene plasmid #12260): transfer vector (pLVX-Tet-on; TRIPZ-shCtrl, TRIPZ-shCAPG2): vesicular stomatitis virus envelop plasmid (pMD2.G, plasmid #12259) at a ratio 5:3:2 by the calcium phosphate method. The culture medium was collected 48 h post-transfection, filtrated using 0.45 µm filters and concentrated at 100-fold by ultracentrifugation at 89,000 × *g* at 4 °C for 1h30. HeLa cells were transduced at a M.O.I = 10 (Multiplicity of Infection) by centrifugation at 1500 × *g* at 30 °C for 90 min in the presence of 5 µg/ml of polybrene.

### Cell transfection and RNA interference

HeLa-S3 and U2OS cells were transfected for 48 h using the lipofectamine RNAiMAX reagent and siRNAs as indicated in Supplementary Table 4. HeLa-S3 shCtrl and shCAPG2 were selected with puromycin (1 μg/mL) and shRNAs were induced with 4 μg/ml of Doxycycline (D3072) for 72 h.

### Flow cytometry

Yeast cells: 500 μL of cells were fixed with 1 mL of ethanol 100%. Cells were centrifuged for 1 min at 16,000 RCF, resuspended in 500 μl of 50 mM Na-citrate pH = 7.5 containing 10 μL of RNase A (10 mg/mL) and incubated 2 h at 50 °C. Then, 10 μL of proteinase K (20 mg/mL) were added and incubated 2 h at 50 °C. Aggregates of cells were dissociated by sonication. 30 μL of cells were incubated with 270 μL of 50 mM Na-citrate pH = 7.5 containing 1X of Sytox (10,000X) for label DNA.

Human cells: Cells were labeled with 10 μM EdU for 20 min. For the fixation, cells were incubated with 1% formaldehyde/PBS in the dark during 10 min at room temperature and washed in 5% BSA in PBS. For the permeabilization, cells were incubated 30 min in the dark at room temperature with 0.2% Triton X-100 in 1% BSA in PBS. Click reaction (PBS, 3 mM CuSO4, 29 μM A488 azide dye, 10 mM sodium ascorbate) was performed during 30 min at room temperature in the dark. Cells were then incubated for 30 min with 0.1 mg/mL RNase and 2 μg/mL DAPI.

For both type of experiments, data were acquired on a MACSQuant and analyzed with FlowJo software.

### Chromatin immunoprecipitation

Chromatin immunoprecipitation was performed as described in Delamarre et al.^26,73^. 5 × 10^8^ cells were crosslinked for 15 min with 1% formaldehyde (Sigma F8775) at RT under agitation. Fixation was quenched by addition of 0.25 M Glycine (Sigma G8898) for 5 min under agitation. Cells were washed three times with cold TBS1X (4 °C). Dry pellets were frozen and conserved at −20 °C. Cell pellets were resuspended in 600 µL lysis buffer (50 mM HEPES-KOH pH7.5, 140 mM NaCl, 1 mM EDTA, 1% Triton X-100, 0.1% Na-deoxycholate) supplemented with 1 mM PMSF and anti-protease (cOmplete Tablet, Roche, 505649001) and lysed by beads-beat method (MB400 U, Yasui Kikai, Osaka). Recovered lysate (WCE, Whole Cell Extract) was sonicated with a Q500 sonicator Bioruptor (4 cycles: 30 s ON, 30 s OFF). Dynabeads were washed three times and resuspended in 1 mL of PBS, 0.5% BSA, 0.1% Tween and incubated with antibodies on a rotating wheel for two h at 4 °C. 2.5 µL of anti-RPA (Agrisera, AS07214) with 90 µL Dynabeads Prot. A (DPA). 20 µl of anti-PK (Anti-V5 tag, AbD Serotec, MCA1360G) with 90 µl DPA. 25 µl of WCE were kept for the Input sample and 25 µl were collected for western-blotting (WB). Antibodies coupled Dynabeads were washed three times with 1 mL of PBS, 0.5% BSA, 0.1% Tween and added to 500 µL of WCE overnight on a rotating wheel at 4 °C. Beads were then collected on a magnetic rack. 25 µl of the supernatant were collected for WB analysis (Flow-Through sample) and beads were washed on ice with cold solutions: two times with Lysis buffer (50 mM HEPES-KOH pH7.5, 140 mM NaCl, 1 mM EDTA, 1% Triton X-100, 0.1% Na-deoxycholate), twice with Lysis buffer + 0.36 M NaCl (50 mM HEPES-KOH pH7.5, 360 mM NaCl, 1 mM EDTA, 1% Triton X-100, 0.1% Na-deoxycholate), twice with Wash buffer (10 mM Tris-HCl pH8, 0.25 M LiCl, 0.5% IGEPAL, 1 mM EDTA, 0.1% Na-deoxycholate) and once with TE (10 mM Tris-HCl pH8, 1 mM EDTA). Antibodies were uncoupled from beads with 120 µl of Elution Buffer (50 mM Tris-HCl pH8, 10 mM EDTA, 1% SDS) for 10 min at 65 °C. 5 µl of eluates were collected for WB (IP sample) and 115 µl were incubated with 120 µl of TE, 0.1% SDS for de-crosslinking at 65 °C overnight. 130 µl of TE containing 60 mg RNase A (Sigma, R65-13) were added to the samples and incubated for 2 h at 37 °C. Proteins were digested by addition of 20 µl of Proteinase K (Sigma, P6556) at 20 mg/ml and incubated for 2 h at 37 °C. 50 µL of 5 M LiCl were added to DNA before purification by two rounds of Phenol: Chloroform: Isoamyl Alcohol 25:24:1 (Sigma, P2069) extractions and precipitation by addition of 100 mM Sodium Acetate (Sigma, S2889), 26 mg/ml of Glycogen (Roche, 10901393001) and two volumes of 100% ethanol overnight at −20 °C. Samples were centrifuged for 45 min at 16.000 RCF at 4 °C, washed with cold 70% ethanol and centrifuged again 15 min at 16.000 RCF at 4 °C. DNA pellets were dried and resuspended in 300 µl of H20 prior to qPCR reactions or in 22 µL prior to deep-sequencing. qPCR reaction was performed with LightCycler480 (Roche) using primers indicated in Supplementary Table 5. IP/Input ratio were calculated and qPCR results were normalized with five negative zones: ChrV, NegV, NTE1, 305 + 11, GLT1. Aspecific IP and western-blotting of the protein samples were performed for each experiment.

### Genome-wide profiling

Genomic DNA was isolated using Qiagen genomic DNA extraction kit. DNA was fragmented using sonication (200–500 bp). ChIP sequencing libraries were prepared using the NEBNext Ultra II DNA Library Prep Kit for Illumina (NEB). Next generation sequencing was performed on a NovaSeq 6000 (Illumina). Single-end reads of 50 bp and paired-end reads were aligned to *S. cerevisiae* genome (2011) sequence with Bowtie/2. ChIP-seq profiles expressed as RPKM were obtained as a ration of IP/input reads. Relative DNA content was determined as a ratio of normalized HU reads to G_1_ reads.

### Western botting

Yeast cells: Total protein extracts extraction was performed as described in Poli et al. 2016.

Human cells: cells pellets were resuspended in 2X Laemmli buffer and treated with Benzonase (25 U/μL) for 30 min at 37 °C twice. Proteins were resolved by SDS-PAGE (Biorad) and then transferred to nitrocellulose membranes. After blocking, proteins were probed with antibodies listed in Supplementary Table 6. Membranes were scanned with a ChemiDoc MP (Biorad).

### DNA fiber spreading and DNA combing

DNA combing was performed as described^29^. For BrdU labeling detection, a rat monoclonal anti-BrdU was used. A click-it reaction kit was used for EdU labeling detection. A mouse monoclonal anti-ssDNA was used to detect DNA molecules. DNA fiber spreading was performed as described previously^38,39^. Briefly, subconfluent cells were sequentially labeled with 10 µM 5-iodo-2’-deoxyuridine (IdU) and with 100 µM 5-chloro-2’-deoxyuridine (CldU) for the indicated times. One thousand cells were loaded onto a glass slide (StarFrost) and lysed with spreading buffer (200 mM Tris-HCl pH 7.5, 50 mM EDTA, 0.5% SDS) by gently stirring with a pipette tip. The slides were tilted slightly and the surface tension of the drops was disrupted with a pipette tip. The drops were allowed to run down the slides slowly, then air dried, fixed in methanol/acetic acid 3:1 for 10 min, and allowed to dry. Glass slides were processed for immunostaining with mouse anti-BrdU to detect IdU, rat anti-BrdU to detect CldU, mouse anti-ssDNA antibodies and corresponding secondary antibodies conjugated to various Alexa Fluor dyes. Antibodies are indicated in Supplementary Table 6.

Images were acquired by using Zeiss Apotome microscope. The length of tracks was measured by using ImageJ software and analysis was performed using GraphPad Prism analysis. The lengths of at least 100 CldU/IdU tracks were measured per sample. To assess statistical significance, the means of at least three biological replicates were compared using a non-parametric test. Because the sample size at the replicate level is small (*n* = 3), differences between replicate means may not reach statistical significance, even when comparisons at the level of individual data points (*n* > 100) appear significant.

### Electronic microscopy

For EM analysis of replication intermediates, 4 µg/mL doxycycline was added to the media of actively replicating shCtrl or shCAPG2 Hela cells for 72 h. In the last 2 h of doxycycline induction, the cells were treated with either 4 mM hydroxyurea alone or combined 4 mM hydroxyurea with 50 µM mirin. Non-treated samples were also included. Cells were immediately collected after treatment, washed, and DNA was cross-linked by incubating with 10 µg/mL 4,5’,8-trimethylpsoralen (T6137, Millipore Sigma) followed by a 3-min exposure to 366 nm UV light on a precooled metal block, for a total of three rounds. Cells were lysed and genomic DNA was isolated from the nuclei by proteinase K (25530-015, Life Technologies) digestion and chloroform-isoamyl alcohol extraction. Genomic DNA was precipitated using isopropanol and digested with *Pvu*II HF (R3151S, New England Biolabs) with the appropriate buffer for 4 h at 37 °C. Replication intermediates were enriched on QIAGEN plasmid mini kit columns (12123, QIAGEN) and concentrated using Amicon Ultra size exclusion columns (UFC510096, Millipore Sigma). Samples were prepared for EM visualization by spreading the enriched, concentrated DNA on a carbon-coated grid in the presence of benzyl-dimethyl-alkylammonium chloride (B6295, Millipore Sigma) as well as n,n-dimethylformamide (227056, Millipore Sigma), followed by low angle platinum rotary shadowing. Images were obtained on a JEOL JEM-1400 electron microscope using a bottom mounted AMT XR401 camera. Analysis was performed using ImageJ software (National Institute of Health). EM analysis allows for distinguishing duplex DNA—which is expected to appear as a 10 nm thick fiber after the platinum coating step necessary for EM visualization—from ssDNA, which has a reduced thickness of 5–7 nm and appears lighter in contrast as that of dsDNA. Criteria used for the assignment of a three-way junction, indicative of a replication fork, include the joining of three DNA fibers into a single junction, with two generally symmetrical daughter strands and a single parental strand. Reversed replication forks consist of four DNA fibers joined at a single junction, consisting of two commonly symmetrical daughter strands, one parental strand and the addition of a typically shorter fourth strand, representative of the reversed arm. The length of the two daughter strands corresponding to the newly replicated duplex oftentimes are equal (b = c), whereas the length of the parental arm and the regressed arm can vary (a ≠ b = c ≠ d). Conversely, canonical Holliday junction structures will be characterized by arms of equal length (a = b, c = d). Particular attention is paid to the junction of the reversed replication fork in order to observe the presence of a bubble structure, indicating that the junction is opened up and that it is simply not the result of the occasional crossover of two DNA molecules. These four-way junctions of reversed replication forks may also be collapsed and other indicators such as daughter strand symmetry, presence of single-stranded DNA at the junction or the entire structure itself, all are considered during analysis^78^. The frequency of reversed forks in a sample is computed using the Prism software.

### Cell survival assay

200 cells were platted and treated during 24 h with HU concentration as indicated. Then, cells were washed and grown during one week. The cells were fixed with cold 80% methanol for 10 min and stained with crystal violet. Distinct colonies were counted.

For drug sensitivity assay, 200 cells were plated. The next day, HU was added as indicated and cells were grown during 3 days. Cells were incubated with Cell Proliferation Reagent WST1 for 1 h and the cell colorimetry was read with an ELISA reader.

### Immunofluorescence microscopy

Cells were incubated 5 min at 4 °C with CSK buffer (20 mM HEPES, 50 mM NaCl, 300 mM sucrose, 3 mM MgCl2, 0.5% Triton X-100, one tablet of protease EDTA-free inhibitor). Then, cells were fixed with 2% paraformaldehyde in PBS for 10 min and permeabilized with 0.5% Triton X-100 for 10 min. The coverslips were incubated 1 hour with an anti-RPA70 antibody (1/500) at 37 °C and then with a secondary antibody conjugated to an Alexa Fluor dye (1/500) for 1 h at 37 °C. The click-it reaction (PBS, 3 mM CuSO4, 29 μM A647 azide dye, 10 mM sodium ascorbate) was performed for 30 min at room temperature in the dark, followed by a DAPI staining and ProlongGold mouting. Images were acquired by using Zeiss Apotome microscope and analyzed with CellProfiler software.

### iPOND

iPOND data was extracted from ref. ^54^.

### Statistical analysis

The number of biological replicates is indicated in the figure legends. Statistical tests were performed with GraphPad Prism 10.

### Reporting summary

Further information on research design is available in the Nature Portfolio Reporting Summary linked to this article.

## Supplementary information

Supplementary Information

Reporting Summary

Transparent Peer Review file

## Source data

Source Data

## Data Availability

The NGS data sets generated and analyzed during the current study are available in NCBI’s Gene Expression Omnibus with the GEO series accession number GSE280436. The iPOND data was extracted from^54^. The data sets generated and/or analyzed during the current study are available from the corresponding authors upon request. Source data are provided with this paper.
