# Supplementary material for: Condensin and topoisomerases cooperate to relieve topological stress at stalled replication forks
Source: Nat Commun. 2026 May 8;17:6211. doi: 10.1038/s41467-026-72936-1 (PMC13369196; doi:10.1038/s41467-026-72936-1)
Supplement: Supplementary file 1 — Supplementary Information [file 41467_2026_72936_MOESM1_ESM.pdf]

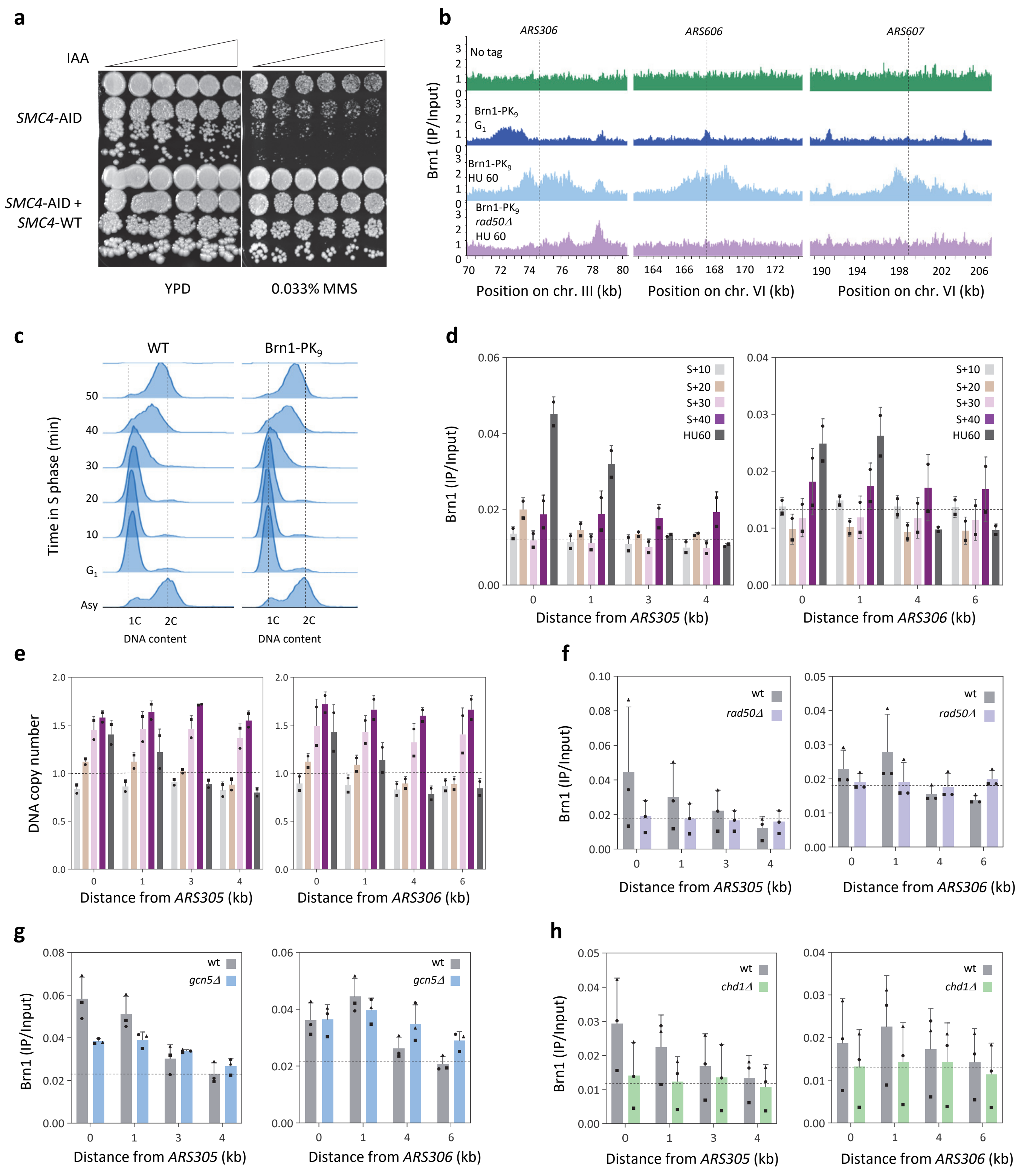

Supplementary Figure 1

Supplementary Fig. 1 Condensin is recruited to replication stress sites in budding yeast in a Rad50-dependent manner. a Condensin promotes cell growth in the presence of MMS. SMC4-AID degron cells complemented or not with WT SMC4 were grown for 4 days on YPD plates containing 0.033% MMS and a 0 to 25 µM gradient of auxin (IAA). **b** Condensin is enriched at active origins in HU-arrested cells. Genome-wide distribution of Brn1-PK9 in wild type (untagged), Brn1-PK9 and *rad50Δ* Brn1-PK9 cells arrested in G_1 with α-factor (G1) or released for 60 min into S phase in the presence of 200 mM HU. Representative regions on chromosomes III and VI are shown. The positions of the early origins *ARS306*, *ARS606* and *ARS607* are indicated. **c-e** Condensin is not enriched at replication sites in the absence of replication stress. Cells were arrested in G_1 with α-factor and were released in S phase for 10, 20, 30 and 40 min or for 60 min into medium containing 200 mM HU. Primers correspond to *ARS305* and *ARS306* and to regions located 1,3 and 4 kb downstream of *ARS305* and 1, 4, and 6 kb upstream of *ARS306*. **c** Analysis of DNA content in normal S phase by flow cytometry in WT and Brn1-PK_9 cells. **d** ChIP-qPCR analysis of Brn1-PK_9 enrichment around early origins. Brn1 enrichment is shown as the signal ratio (IP/Input). Mean +/- SD correspond to two independent experiments. **e** Origin firing and DNA replication progression were monitored by analyzing DNA copy number variation. DNA was extracted and analyzed by qPCR using the same primers as in d, corresponding to the *ARS305* and *ARS306* replication origins, and to a late-replicating negative control region. Data represent the mean +/- SD from two independent experiments. **f** Condensin enrichment at stressed forks depends on the MRX subunit Rad50. Wild-type (WT) and *rad50Δ* cells expressing *BRN1-PK$_9$* were released from G_1 into S phase for 60 min in the presence of 200 mM HU. Brn1 enrichment was determined by ChIP–qPCR at the indicated distances from *ARS305* and *ARS306*. Brn1 enrichment is shown as the signal ratio (IP/Input). Data represent the mean ± SD of three independent experiments. **g,h** Condensin binding to HU-arrested forks depends on the Gcn5 histone acetyltransferase and the Chd1 chromatin remodeler. Brn1 enrichment at increasing distances from the early origins *ARS305* and *ARS306* was determined by ChIP–qPCR in wild-type (grey), *gcn5Δ* (blue), and *chd1Δ* (green) cells. Cells were released from G_1 into S phase for 60 min in the presence of 200 mM HU. Brn1 enrichment is shown as the signal ratio (IP/Input). Data represent the mean +/- SD of three independent experiments.

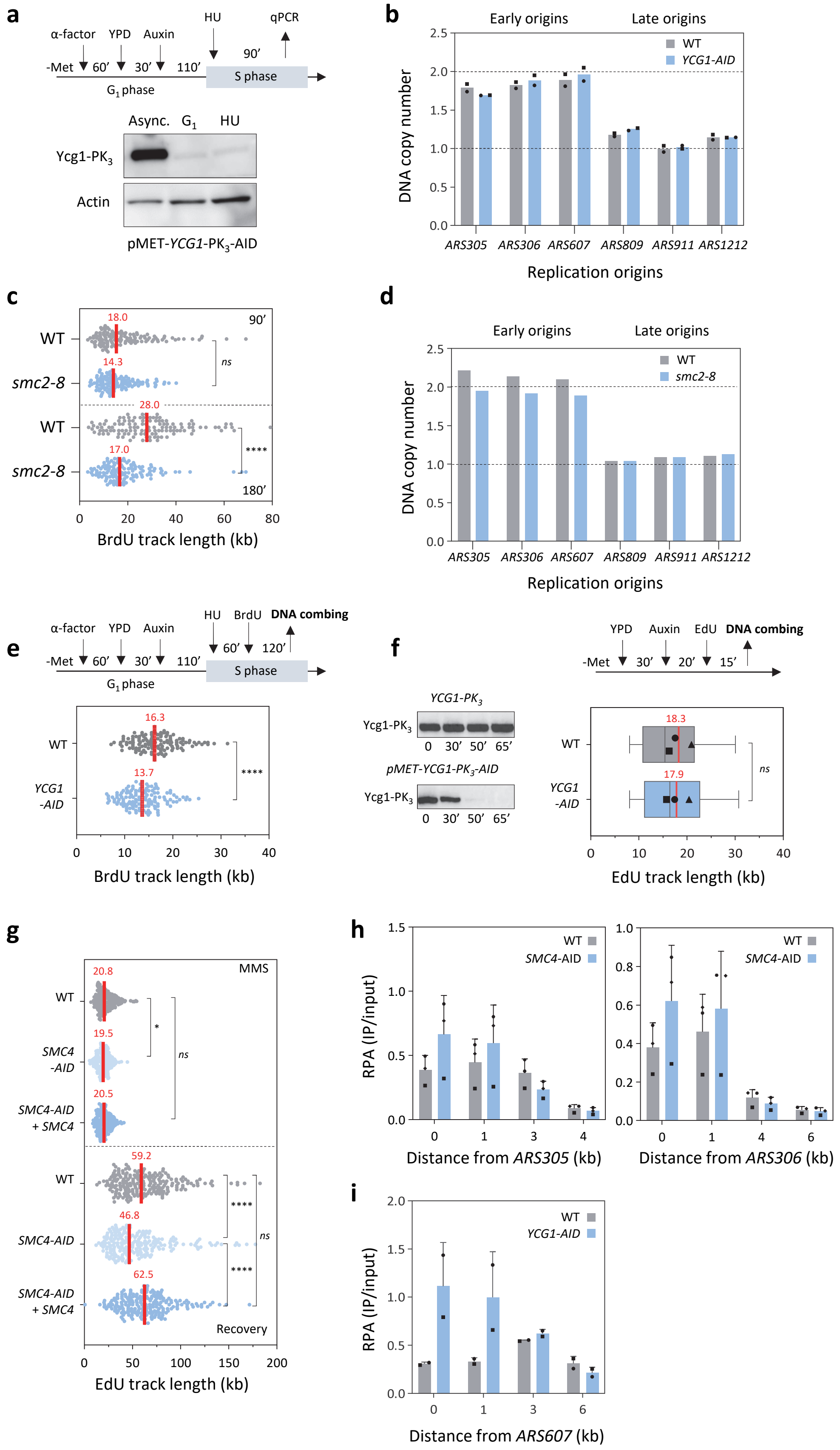

Supplementary Figure 2

Supplementary Fig. 2 Condensin is dispensable for normal fork progression and for checkpoint activation, but contributes to fork restart in budding yeast. a, b The replication checkpoint remains functional in cells depleted for the condensin subunit Ycg1. WT and pMET-*YCG1*-PK_3-AID cells grown in medium without methionine were synchronized in G_1 with α-factor and released into S phase in the presence of 200 mM HU. Cells were shifted to YPD medium after 60 min of α-factor addition and auxin was added 100 min before G_1 release to deplete Ycg1. Origin firing was monitored by DNA copy number variation. DNA was extracted and analyzed by qPCR with primers corresponding to the indicated early and late origins. DNA content was normalized to a late-replicating negative control region in two independent experiments. The repression of the late origins *ARS809*, *ARS911* and *ARS1212* is indicative of the timely activation of the replication checkpoint in the presence of HU. **c** Fork progression is impaired in HU-treated *smc2-8* mutants. A representative experiment from Fig. **2a** (n=3) is shown. Median length is indicated. ****: p<0.0001; ns: non-significant (Mann–Whitney rank-sum test). **d** Origin firing was monitored in WT and *smc2-8* cells by analyzing DNA copy number variation in cells released synchronously into S phase at 35°C in the presence of 200 mM HU for 60 min. **e** Ycg1-depleted cells show a slower fork progression in the presence of 200 mM HU. Cells were grown as described in panel a. BrdU was added after 60 min in HU for 120 min. Median length is indicated. ****: p<0.0001 (Mann–Whitney rank-sum test). **f** Condensin is dispensable for normal fork progression. Exponentially growing *YCG1*-PK_3 and pMET-*YCG1*-PK_3-AID cells grown in medium lacking methionine were transferred for 30 min into YPD medium before auxin addition to deplete Ycg1. EdU was then added for 15 min to label newly replicated DNA and the length of EdU tracks was measured by DNA combing. Box and whiskers indicate median, 25th–75th and 10th–90th percentiles. Mean values are indicated. ns: non-significant (unpaired t-test on means, two-tailed). **g** Condensin is required for timely fork restart after MMS exposure. A representative experiment from Fig. **2b** (n=3) is shown. Median is indicated ****: p<0.0001; *: p<0.05; ns: non-significant (Mann–Whitney rank-sum test). **h** RPA-coated ssDNA accumulates at HU-arrested forks in yeast cells lacking condensin. WT and *SMC4*-AID cells were arrested in G_1 with α-factor and SMC4 was depleted by addition of auxin for 60 min. Then, cells were released into S phase in the presence of 200 mM HU for another 60 min. RPA enrichment at indicated distances from *ARS305* and *ARS306* was determined by ChIP-qPCR and is shown as the signal ratio (IP/Input). Data represent the mean ± SD of three independent experiments. **i** WT and pMET-*YCG1*-PK_3-AID cells were synchronized in G_1 with α-factor and released into S phase in the presence of 200 mM HU. RPA enrichment was determined by ChIP-qPCR at the indicated distances from *ARS607* and is shown as the signal ratio (IP/Input). Data represent the mean +/- SD of two independent experiments.

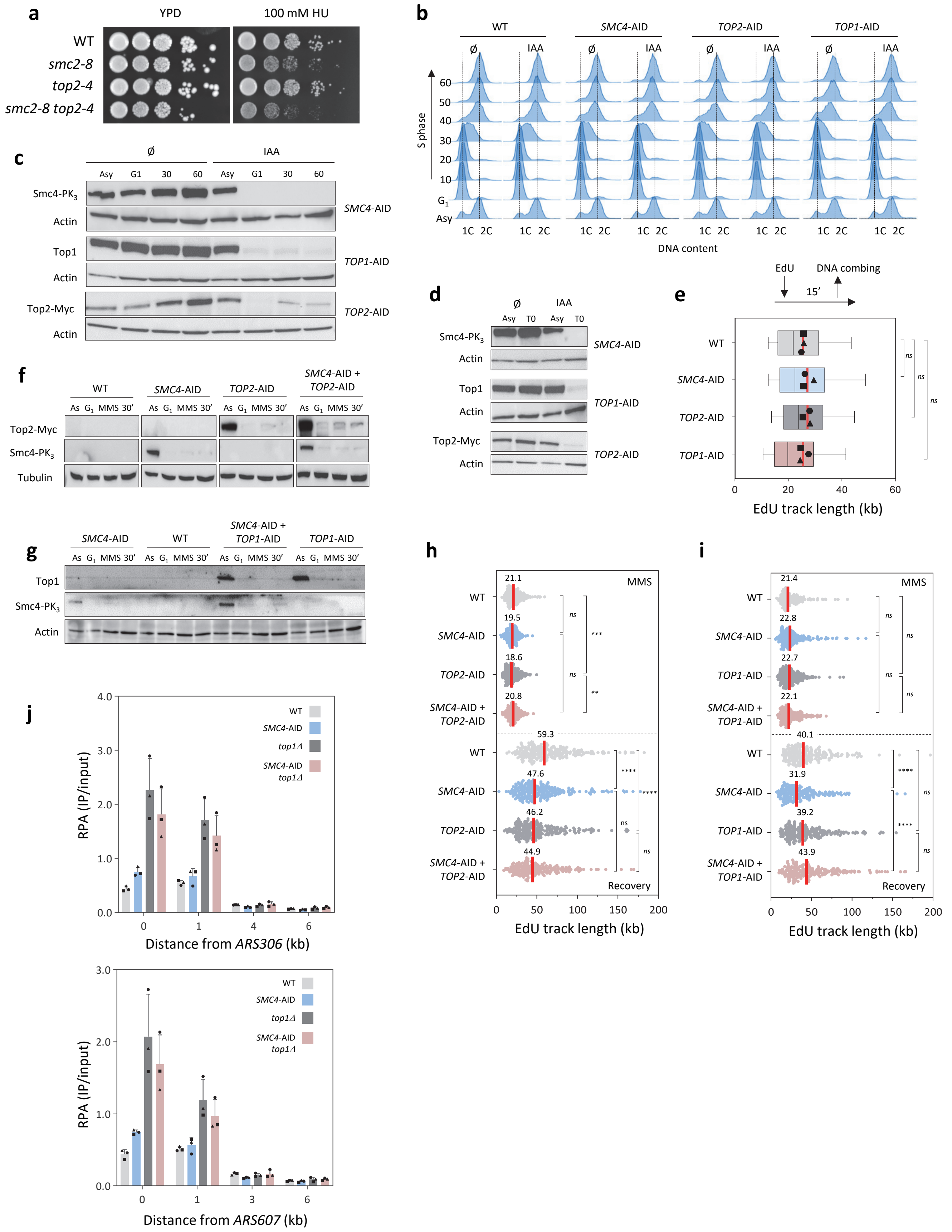

Supplementary Figure 3

Supplementary Fig. 3 Topoisomerases and condensin promote fork processing upon replication stress. a The *top2-4* mutation does not exacerbate the growth defect of a condensin mutant under replication stress. Wild-type (WT), thermosensitive *smc2-8*, *top2-4* and *smc2-8 top2-4* cells were grown for 5 days on YPD medium containing 100 mM HU at the semi-permissive temperature of 30 °C. **b** Flow cytometry analysis of cell-cycle progression in wild-type, *SMC4-AID*, *TOP2-AID*, and *TOP1-AID* cells. Cells were synchronized in G_1 with α-factor and released into S phase in the presence or absence of auxin (IAA) to induce degradation of AID-tagged proteins. DNA content was monitored by flow cytometry at the indicated time points to assess cell-cycle progression. **c** Western blot analysis of Smc4, Top1 and Top2 depletion in the experiment described in panel **b**. **d, e** Acute depletion of Smc4, Top2, and Top1 does not affect DNA replication under unperturbed conditions. **d** Western blot showing auxin-induced depletion of AID-tagged proteins in asynchronously growing WT, Smc4-PK3-mAID, Top2-AID-Myc, and Top1-AID cells after 60 min of auxin treatment. **e** Distribution of EdU track lengths measured by DNA combing following a 15 min EdU pulse. Box and whiskers plots (median, 25th–75th and 10th–90th percentiles) and means (red) represent data from three independent experiments. Statistical significance was determined using an unpaired t-test on mean values, two-sided. ns: not significant. **f-g** Western blot analysis of Top1 and Top2 depletion in the experiments described in panels **h** and **i**. **h** Condensin acts with Top2 to promote fork restart after MMS exposure. A representative experiment from Fig. **3b** (n=3) is shown. Median is indicated. ****: p<0.0001; ***: p<0.001; **: p<0,01; ns: non-significant (Mann–Whitney rank-sum test). **i** Top1 depletion restores fork restart in the absence of condensin. A representative experiment from Fig. **2c** (n=3) is shown. Median is indicated. ****: p<0.0001; ns: non-significant (Mann–Whitney rank-sum test). **j** RPA-coated ssDNA accumulates at HU-arrested forks in yeast cells lacking condensin and Top1. WT, *SMC4*-AID, *top1Δ* and *top1Δ SMC4*-AID cells were arrested in G_1 with α-factor and SMC4 was depleted by addition of auxin for 60 min. Then, cells were released into S phase in the presence of 200 mM HU for another 60 min. RPA enrichment at indicated distances from *ARS306* and *ARS607* was determined by ChIP-qPCR and is shown as the signal ratio (IP/Input). Data represent the mean ± SD of three independent experiments.

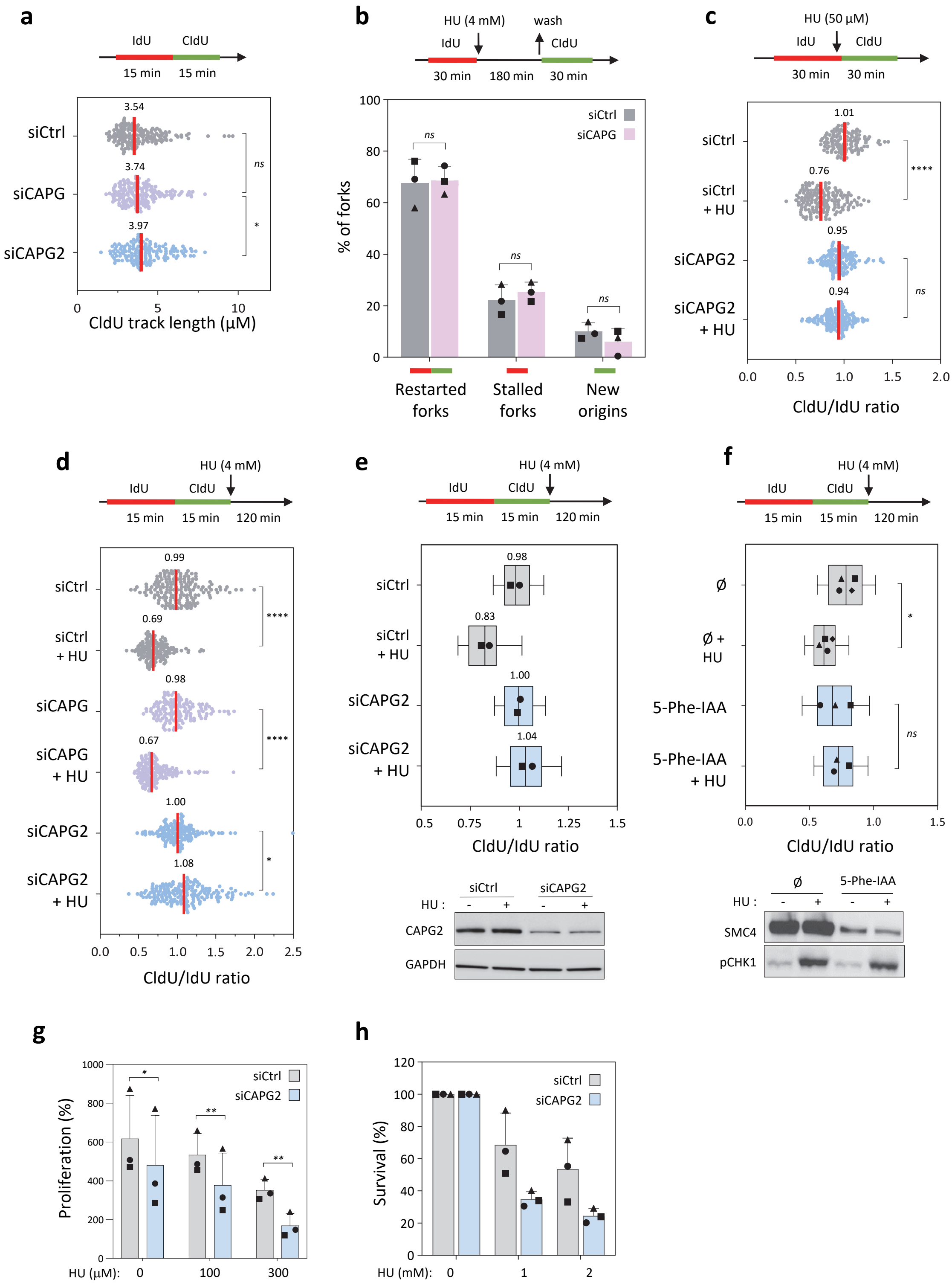

Supplementary Figure 4

Supplementary Fig. 4 Condensin promotes cell growth under replication stress conditions. a Condensin I and II are dispensable for normal fork progression. A representative experiment from Fig. **3b** is shown. *: p<0.05; ns: non-significant (Mann–Whitney rank-sum test). **b** Condensin I is not involved in fork restart. U2OS cells were transfected with siCtrl or siCAPG for 48 h and were treated with 4 mM HU for 3 h after a 30 min IdU pulse. IdU and CldU tracks were analyzed by DNA fiber spreading 30 min after HU removal and CldU addition. Red and green signals are indicative of fork restart. Red only tracks correspond to stalled forks and green tracks to new origin firing (n = 3). Two-way ANOVA test was used. Post-hoc comparisons between siCTL and siCAPG for each type of forks were performed for multiple comparisons (1 family). ns: not significant. **c** Condensin II is required for fork slowing after exposure to a low dose of HU. A representative experiment from Fig. **3e** is shown. ****: p< 0.0001; ns: non-significant, Mann–Whitney rank-sum test. **d** Condensin II, but not Condensin I, promotes the resection of nascent DNA at HU-arrested forks. HeLa-S3 cells were transfected with siCtrl, siCAPG or siCAPG2 for 48 h and were sequentially labeled for 15 min with IdU and CldU. Cells were either collected immediately or treated for 2 h with 4 mM HU before DNA fiber analysis. The ratio of CldU to IdU track length is shown (n=3). ****: p< 0.0001; *: p< 0.05, Mann–Whitney rank-sum test. **e** Condensin II promotes the resection of nascent DNA at HU-arrested forks in U2OS cells. U2OS cells were transfected with siCtrl and siCAPG2 for 48 h. After sequential labelling of IdU and CldU for 15 min, cells were either collected immediately or treated for 2 h with 4 mM HU before DNA fiber analysis. The ratio of CldU to IdU track length was plotted for two independent experiments. Box and whiskers indicate median, 25th–75th and 10th–90th percentiles. Unpaired t-test on mean values, two-sided. **f** SMC4-mAID-Halo cells were treated or left untreated with 5-Phe-IAA for 2.5 h, followed by sequential labeling with IdU and CldU for 15 min each. Cells were then either collected immediately or treated with 4 mM HU for 2 h before DNA fiber analysis. The ratio of CldU to IdU track length was plotted for two independent experiments. Box and whiskers indicate median, 25th–75th and 10th–90th percentiles. Unpaired t-test on mean values, two-sided. **g** Condensin II is required for growth in the presence of HU. HeLa-S3 cells were transfected with siRNAs (siCtrl or siCAPG2) for 48 h. Cells were incubated with increasing doses of HU for 3 days. Cell proliferation was quantified and normalized to control cells at day 0. Mean and SD are indicated (n=3). Two-way ANOVA test was used. Post-hoc comparisons between siCTL and siCAPG for each concentration of HU were performed for multiple comparisons (1 family). **: p<0.01; *: p<0.05. **h** Condensin II promotes cell survival after acute HU exposure. HeLa-S3 cells were transfected with siRNAs (siCtrl or siCAPG2) for 48 h. Cells were incubated for 24 h with 1 or 2 mM of HU. Colonies were counted after 7 days and the percentage of survival for each cell line was normalized to untreated condition. Mean +/- SD are indicated (n = 3).

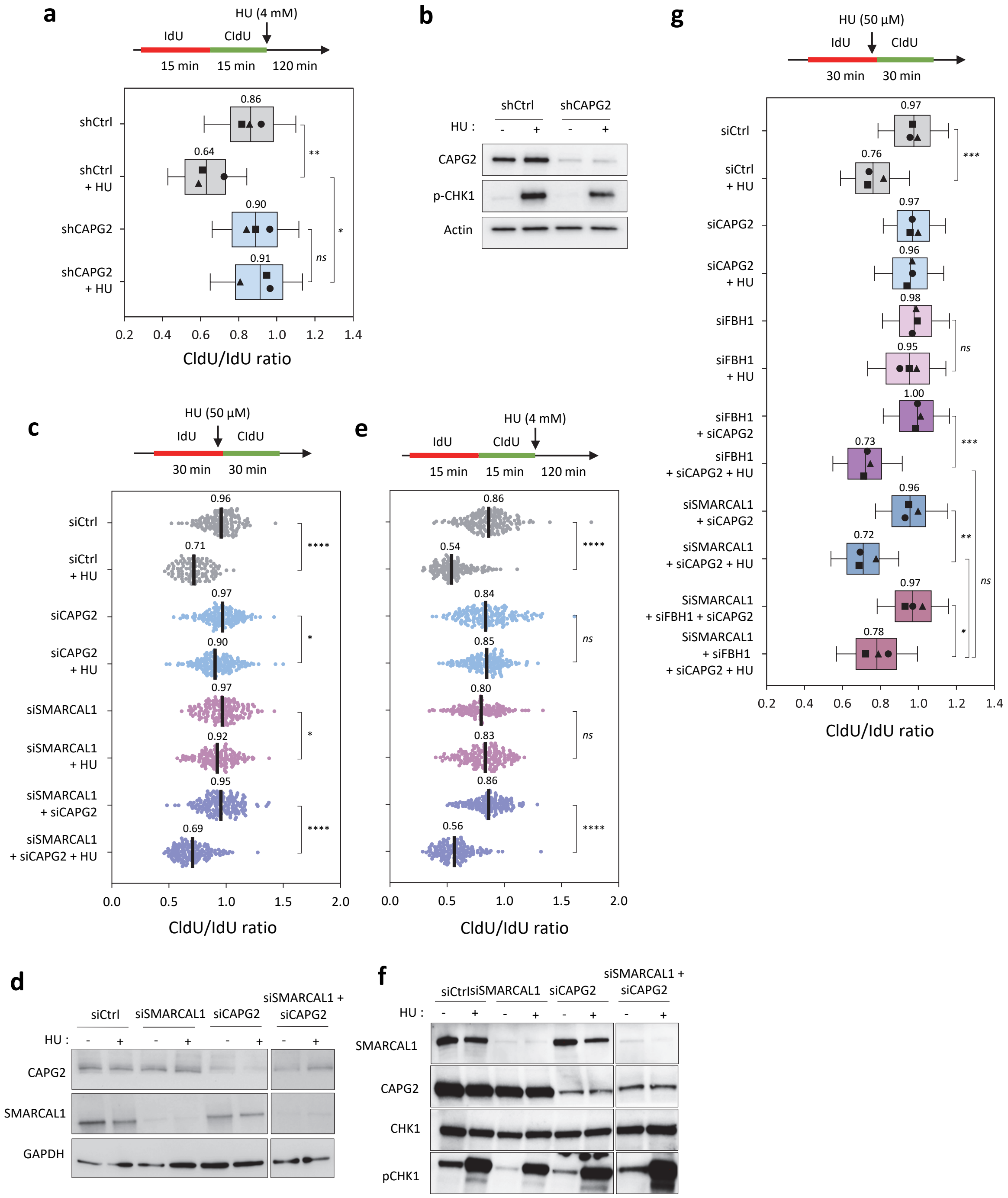

Supplementary Figure 5

Supplementary Fig. 5 Condensin II acts with DNA translocases to promote fork reversal. a Control (shCtrl) and CAPG2-depleted cells (shCAPG2) HeLa-S3 cells were treated for 72 h with 10 μM/ml doxycycline and then for 2 h with 4 mM HU, under the same experimental conditions used for EM analysis (**Fig. 4a** and **b**). Fork resection was monitored by DNA fiber spreading after sequential labelling with IdU and CldU for 15 min. Cells were either collected immediately or treated for 2 h with 4 mM HU before DNA fiber analysis. Box and whiskers indicate median, 25th–75th and 10th–90th percentiles (n=3). Mean is indicated. **: $p<0.01$; *: $p<0.05$, ns: non-significant (paired t-test on means). **b** CAPG2 depletion was analyzed by Western blotting. CHK1 phosphorylation was detected with an anti-p-CHK1 (S345) antibody. Actin was used as loading control. **c** Defective fork slowdown in SMARCAL1-deficient cells is rescued by CAPG2 depletion. A representative experiment from Fig. **4d** is shown. ****: $p<0.0001$; *: $p<0.05$ (Mann–Whitney rank-sum test). **d** Analysis of CAPG2 and SMARCAL1 depletion by Western blotting. GAPDH is used as loading control. **e** CAPG2 depletion restores fork resection in SMARCAL1-depleted cells. A representative experiment from Fig. 4e is shown. ****: $p<0.0001$; ns: non-significant (Mann–Whitney rank-sum test). **f** Analysis of CAPG2 and SMARCAL1 depletion by Western blotting. CHK1 phosphorylation was detected with an anti-pCHK1 (S345) antibody. Total CHK1 was used as loading control. **g** CAPG2 depletion restores fork slowdown in both SMARCAL1- and FBH1-depleted cells. U2OS cells were transfected for 48 h with siCtrl, siCAPG2, siFBH1, or co-transfected with siSMARCAL1 as indicated. Cells were first labelled for 30 min with IdU, and CldU was added for another 30 min in the presence of 50 μM HU. The ratio of CldU to IdU track length was plotted for four independent experiments. Box and whiskers indicate median, 25th–75th and 10th–90th percentiles. Mean is indicated. ***: $p<0.001$, **: $p<0.01$, *: $p<0.05$ (unpaired t-test on means, two-sided).

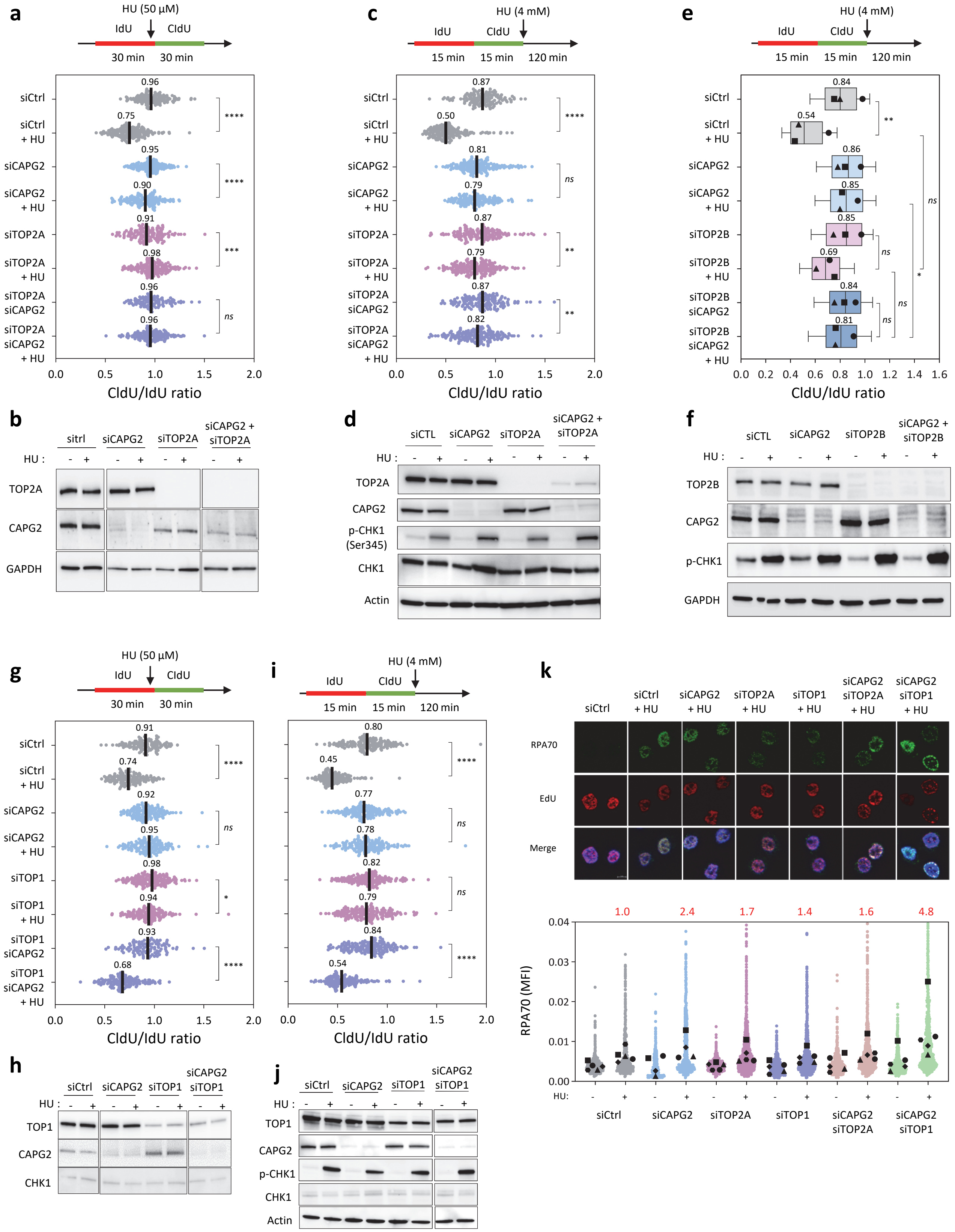

Supplementary Figure 6

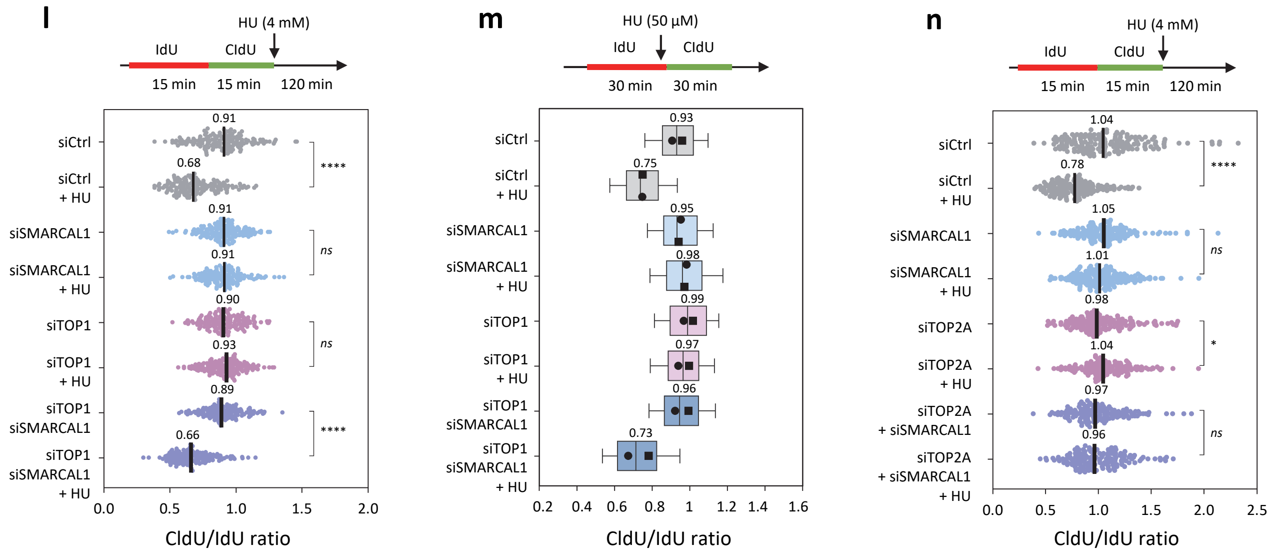

Supplementary Figure 6 (*continued*)

Supplementary Fig. 6 Interplay between condensin II and topoisomerases at stalled replication forks. a Condensin II acts with TOP2A to promote fork slowing. A representative experiment from Fig. **6b** is shown. ****: p<0.0001; ***: p<0.001; ns: non-significant (Mann–Whitney rank-sum test). **b** TOP2A and CAPG2 levels were analyzed by Western blotting using GAPDH as loading control. **c** Condensin II acts with TOP2A to promote fork resection. A representative experiment from Fig. **6c** is shown. ****: p<0.0001; **: p<0.01; ns: non-significant (Mann–Whitney rank-sum test). **d** Western blot analysis of TOP2A and CAPG2 depletion. CHK1 activation was detected with an anti-pCHK1 (S345) antibody. Total CHK1 and actin were used as loading controls. **e** TOP2B act with CAPG2 to promote fork resection. HeLa-S3 cells were transfected with siCtrl, siCAPG2, siTOP2B or co-transfected with siTOP2B and siCAPG2 for 48 h. After sequential labelling of IdU and CldU for 15 min, cells were either collected immediately or treated for 2 h with 4 mM HU before DNA fiber analysis. The ratio of CldU to IdU track length is shown for three independent experiments. Box and whiskers correspond to median, 25th–75th and 10th–90th percentiles. Mean length is indicated. **: p<0.01, *: p<0.05; ns: non-significant (unpaired t-test on means, two-sided). **f** Western blot analysis of TOP2B and CAPG2 levels. CHK1 activation was detected with an anti-pCHK1 (S345) antibody. GAPDH was used as loading control. **g** TOP1 depletion restores fork slowing in CAPG2-deficient cells. A representative experiment from Fig. **6d** is shown. ****: p<0.0001; *: p<0.05; ns: non-significant (Mann–Whitney rank-sum test). **h** TOP1 and CAPG2 levels were analyzed by Western blotting using total CHK1 is used as loading control. **i** TOP1 depletion restores fork resection in CAPG2-deficient cells. A representative experiment from panel **6e** is shown. ****: p<0.0001; ns: non-significant (Mann–Whitney rank-sum test). **j** Western blot analysis of TOP1 and CAPG2 levels and CHK1 activation. Total CHK1 and actin were used as loading controls. **k** Levels of chromatin-bound RPA increase in the absence of both CAPG2 and TOP1. HeLa-S3 cells were transfected for 48 h with siCtrl, siCAPG2, siTOP2A, siTOP1 or co-transfected as indicated. Cells were labeled with EdU and were treated with 4mM of HU for 2 h. RPA70 (green) and EdU-labelled replication sites (red) were immunodetected. The mean fluorescence intensity (MFI) of RPA70 was quantified from five independent experiments using CellProfiler. Changes in median intensity relative to control cells are indicated in red. **l** TOP1 depletion restores fork resection in SMARCAL1-deficient cells. A representative experiment from panel **6f** is shown. ****: p<0.0001; ns: non-significant (Mann–Whitney rank-sum test). **m** TOP1 depletion restores fork slowing in SMARCAL1-deficient cells. HeLa-S3 cells were transfected with siCtrl, siSMARCAL1, siTOP1, or co-transfected with siSMARCAL1 and siTOP1 for 48 h. Cells were labeled and analyzed by DNA fiber spreading as described in Fig. 6c (n=2). Box and whiskers correspond to median, 25th–75th and 10th–90th percentiles. Mean is indicated. **n** Depletion of TOP2A does not restore fork resection in SMARCAL1-deficient cells. A representative experiment from Fig. 6g is shown. ****: p<0.0001; *: p<0.05; ns: non-significant (Mann–Whitney rank-sum test).

SUPPLEMENTARY TABLES

Supplementary Table 1: Summary of p-values values for Fig. 2 and 3. p-values were calculated using Mann–Whitney tests for individual experiments and unpaired t-tests to compare means of biological replicates.

Figure panels	Condition	Biological replicates (median)	Mann Whitney test (comparison of individual datasets)	Biological replicates (mean)	t test (comparison of means)
Fig. 2a	WT HU90'	18.0; 9.2; 13.0	-	23.1; 10.9; 14.3	-
	smc2-8 HU90'	14.3; 8.4; 14.4	**; ns; ns	16.4; 9.1; 16.3	ns
	WT HU 180' HU	27.9; 19.8; 18.7	-	31.8; 22.2; 24.0	-
	smc2-8 180' HU	17.0; 14.9; 14.2	****; **; ***	19.3; 18.6; 17.1	ns
Fig. 2b	WT MMS	21.2; 21.1; 19.9	-	21.6; 22.9; 20.5	-
	SMC4-AID MMS	22.0; 19.5; 19.2	ns; **; ns	21.7; 20.2; 20.2	ns
	SMC4-AID + *SMC4* WT MMS	20.2; 20.5; 19.2	ns; ns; ns	21.1; 21.0; 19.6	ns
	WT Recovery	60.6; 59.3; 57.9	-	68.1; 65.5; 67.3	-
	SMC4-AID Recovery	51.2; 47.6; 46.8	****; ****; ****	57.6; 55.6; 50.7	**
	SMC4-AID + *SMC4* WT Recovery	57.0; 62.5; 65.3	ns; ns; **	66.1; 65.0; 75.2	ns
Fig. 3b	WT	60.63; 59.26; 57.88	-	68.1; 65.5; 67.0	-
	SMC4-AID	51.17; 47.63; 46.83	****; ****; ****	57.6; 55.6; 50.7	**
	TOP2-AID	56.6; 46.1; 51.2	*; ****; **	61.9; 53.1; 55.2	*
	TOP2-AID *SMC4*-AID	37.1; 44.9; 48.0	****; ns; ns (vs *SMC4*-AID) ****; ns; * (vs *TOP2*-AID)	49.4; 51.4; 51.8	ns (vs *SMC4*-AID) ns (vs *TOP2*-AID)
Fig. 3c	WT	40.1; 45.0; 41.6	-	47.9; 54.0; 50.4	-
	SMC4-AID	31.9; 26.0; 36.1	****; ****; *	38.6; 34.5; 44.0	*
	TOP1-AID	39.2; 49.7; 34.9	ns; **; **	46.2; 63.8; 42.8	ns
	TOP1-AID *SMC4*-AID	43.9; 47.8; 56.0	****; ****; **** (vs *SMC4*-AID) ns; ns; **** (vs *TOP1*-AID)	53.0; 60.2; 68.2	* (vs *SMC4*-AID) ns (vs *TOP1*-AID)

Supplementary Table 2: Individual EM experiments

	siCtrl<br>NT	siCtrl<br>HU	siCtrl<br>HU+Mirin	siCAPG2<br>NT	siCAPG2<br>HU	siCAPG2<br>HU+Mirin
E1	3<br>(86)	20<br>(82)	23<br>(71)	3<br>(68)	12<br>(92)	8<br>(80)
E2	9<br>(75)	25<br>(68)	23<br>(78)	7<br>(76)	13<br>(80)	12<br>(77)

*Percentage of reversed forks – top number
Number of molecules analyzed – bottom number

Supplementary Table 3: Yeast strains used in this study

Name	Genotype	Figure
PP560	MATalpha, ade2-1, trp1-1, can1-100, leu2-3,112, his3-11,15, ura3-1, GAL, RAD5, top2-4	S3a
PP870	MATa, ade2-1, trp1-1, can1-100, leu2-3,112, his3-11,15, ura3, GAL, psi+, RAD5	1A
PP872	MATa, ade2-1, trp1-1, can1-100, leu2-3,112, his3-11,15, ura3, GAL, psi+, RAD5, URA3::GPD-TK7	1b, 1c, 1d, S1b, S1c, S1d, S1e, 2a, 2d, S2c, S2d, 3a, S3a
PP1919	MATa, ade2-1, trp1-1, can1-100, leu2-3,112, his3-11,15, ura3, GAL, psi+, RAD5, URA3::GPD-TK7 Brn1-PK9::TRP	1b, 1c, 1d, 1e, S1b, S1c, S1d, S1e, S1f, S1g
1923	MATa, RAD5, URA3::GDP-TK7, smc2.8::KAN	2a, 2d, S2c, S2d, 3a S3a
PP1941	MATa, ade2-1, trp1-1, can1-100, leu2-3,112, his3-11,15, ura3, GAL, psi+, RAD5, URA3::GPD-TK7 Brn1-PK9::TRP, rad50::KAN	S1b, S1f
PP2041	MATa, ade2-1, trp1-1, can1-100, leu2-3,112, his3-11,15, ura3, GAL, psi+, RAD5, smc2-8::KAN	1a
2071	MATalpha, ade2-1, trp1-1, can1-100, leu2-3,112, his3-11,15, ura3-1, GAL, RAD5, top2-4, URA3::GPD-TK7, smc2.8::KAN	S3a
PP2103	MATa, ade2-1, trp1-1, can1-100, leu2-3,112, his3-11,15, ura3, GAL, psi+, RAD5, Brn1-PK9::TRP	1f, S1h
PP2405	MATa, ade2-1, trp1-1, can1-100, leu2-3,112, his3-11,15, ura3, GAL, psi+ , RAD5, TK-hENT1::LEU2, ADH1pr-O.s.Tir1-myc9::TRP1	2c, 3d, S3j
PP2408	MATa, ade2-1, trp1-1, can1-100, leu2-3,112, his3-11,15, ura3, GAL, psi+ , RAD5, TK-hENT1::LEU2, ADH1pr-O.s.Tir1-myc9::TRP1, SMC4-3PK-1xminiAID-KAN	2c, 3d, S3j
PP2658	MATalpha, ade2-1, trp1-1, can1-100, leu2-3,112, his3-11,15, ura3, GAL, psi+, RAD5, top1::NAT	3a
PP2668	MATa, ade2-1, trp1-1, can1-100, leu2-3,112, his3-11,15, ura3, GAL, psi+ , RAD5, , ADH1pr-O.s.Tir1-myc9::TRP1, SMC4-3PK-1xminiAID-KAN, top1::NAT	3d, S3j
PP2670	MATa, ade2-1, trp1-1, can1-100, leu2-3,112, his3-11,15, ura3, GAL, psi+ , RAD5, , ADH1pr-O.s.Tir1-myc9::TRP1, top1::NAT	3d, S3j
PP2685	MATa, ade2-1, trp1-1, can1-100, leu2-3,112, his3-11,15, ura3, GAL, psi+, RAD5 , smc2-8::KAN, top1::NAT	3a
PP3136	MATa, ade2-1, trp1-1, can1-100, leu2-3,112, his3-11,15, ura3, GAL, psi+, RAD5, Brn1-PK9::TRP, gcn5::HIS	1e, S1g

3320	MATalpha, ade2-1, trp1-1, can1-100, leu2-3,112, his3-11,15, ura3, GAL, rfa1(G77E)-HA3::KAN	2d
3556	MATa, ade2-1, trp1-1, can1-100, leu2-3,112, his3-11,15, ura3, GAL, psi+, RAD5, smc2.8::KAN, rfa1(G77E)-HA3::KAN	2d
3601	MATa, ade2-1, trp1-1, can1-100, leu2-3,112, his3-11,15, ura3, GAL, psi+, RAD5, Brn1-PK9::TRP, chd1::TRP	1f, S1h
PP4947	MATa,ade2-1, trp1-1, can1-100, leu2-3,112, his3-11,15, ura3, GAL, psi+, RAD5+, ade2-1::OsTIR1-9myc:ADE2 ura3-1::ADH1-OsTIR1-2-9Myc (URA3) SMC4-3Pk-miniAID:kanR, AUR1::yTK-yENT1::AUR1C	S1a, 2b, S2g, 3b, 3c, S3b, S3c, S3d, S3e, S3f, S3g, S3h, S3i
PP4948	MATa,ade2-1, trp1-1, can1-100, leu2-3,112, his3-11,15, ura3, GAL, psi+, RAD5+, ade2-1::OsTIR1-9myc:ADE2 ura3-1::ADH1-OsTIR1-2-9Myc (URA3) SMC4-3Pk-miniAID:kanR, trp1-1::PSMC4–SMC4WT-3HA:TRP1, AUR1::yTK-yENT1::AUR1C	S1a, 2b, S2g
PP4949	MATa, leu2-3,112 his3-11,15 ade2-1 trp1-1 can1-100 RAD5+ ura3-1::pADH1-OsTIR1-URA3, ade2-1::OsTIR1-9myc:ADE2, TOP2-AID*-MYC-HPH, AUR1::yTK-yENT1::AUR1C	3b, S3b, S3c, S3d, S3e, S3f, S3h
PP4950	MATa, leu2-3,112 his3-11,15 ade2-1 trp1-1 can1-100 rad5-535 ura3-1::pADH1-OsTIR1-URA3, ade2-1::OsTIR1-9myc:ADE2, AUR1::yTK-yENT1::AUR1C	2b, S2g, 3b, S3f, S3h
PP4952	MATa, leu2-3,112 his3-11,15 ade2-1 trp1-1 can1-100 RAD5+ ura3-1::pADH1-OsTIR1-URA3, ade2-1::OsTIR1-9myc:ADE2, TOP2-AID*-MYC-HPH, SMC4-3Pk-miniAID:kanR, , AUR1::yTK-yENT1::AUR1C	3b, S3f, S3h
PP5214	MATa, leu2-3,112 his3-11,15 ade2-1 trp1-1 can1-100 rad5-535 ura3-1::pADH1-OsTIR1-URA3, ade2-1::OsTIR1-9myc:ADE2, AUR1::yTK-yENT1::AUR1C	3c, S3b, S3c, S3d, S3e, S3g, S3i
PP5481	MATa, ade2-1, trp1-1, can1-100, leu2-3,112, his3-11,15, ura3, GAL, psi+, RAD5+, YCG1-3PK:: K.lactis TRP1, AUR1::yTK-yENT1::AUR1C	S2a, S2b, S2e, S2f, S2h, S2i
PP5579	MATa, ade2-1, trp1-1, can1-100, leu2-3,112, his3-11,15, ura3, GAL, psi+, rad5+, (K.lactis URA3)pMET3-YCG1-3PK-miniAID::KANMX6, ADE2::pADH1-OsTIR1-9Myc, AUR1::yTK-yENT1::AUR1C	S2a, S2b, S2e, S2f, S2h, S2i
PP5589	MATa,ade2-1, trp1-1, can1-100, leu2-3,112, his3-11,15, ura3, GAL, psi+, RAD5+, ade2-1::OsTIR1-9myc:ADE2 ura3-1::ADH1-OsTIR1-2-9Myc (URA3) SMC4-3Pk-miniAID:kanR TOP1-AID-KanMX6? AUR1::yTK-yENT1::AUR1C	3c, S3g, S3i
PP5599	MATa,ade2-1, trp1-1, can1-100, leu2-3,112, his3-11,15, ura3, GAL, psi+, RAD5+, ade2-1::OsTIR1-9myc:ADE2 ura3-1::ADH1-OsTIR1-2-9Myc (URA3) TOP1-AID-KanMX6 AUR1::yTK-yENT1::AUR1C	3c, S3b, S3c, S3d, S3e, S3g, S3i

Supplementary Table 4: siRNA or shRNA used in this study

Name	Sequence	References	Supplier	
siCtrl	5' AGGUAGUGUAAUCGCCUUG 3'	Custom		
siTOP2A	5' CCACGAAUAACCAUAGAAA 3'	Custom		Tian *et al.* 2021
siTOP2B	5' GCUAAAUGGAACAGAUAAA 3'	Custom		Tian *et al.* 2021
siTOP1	5' GGAUGAUCUUUUUGAUAGAtt 3'	s14304	Ambion	
siCAPG	5' CUUAAAGUCUCAUGAAGCAAACAGC 3'	Custom		
siCAPG2	5' GCCCUACUGGAAUGUGUUAUUAUA 3'	Custom		
siSMARCAL1	5' UUGCUAAGAAGGUCAAAGC 3'	Custom		
FBH1 #5	5' CCUCAACGCUGGUCAAGUA 3'	J-017404-05-0002	Dharmacon	Liu *et al.* 2020
FBH1 #6	5' AGGGAAGGGUGGAUUCAUA 3'	J-017404-06-0002		Liu *et al.*2020
FBH1 #7	5' GUGCCUAUUUGGUGUAAGA 3'	J-017404-07-0002		Liu *et al.* 2020
FBH1 #8	5' AAACAAAACCUCGUCAUUA 3'	J-017404-08-0002		Liu *et al.* 2020
shCAPG2	piSMART-PGK/TurboRFP	V3SH11252-225244908	Dharmacon	

Supplementary Table 5: Oligonucleotides used in this study and previously in Delamarre *et al*.2020.

	Sequence	
Name	Forward	Reverse
305	AGCCTTCTTTGGAGCTCAAGTG	TTTGAGGAATTTCTTTTGAAGAGTTG
305+1	TCCAATTATATGGCACCGAGATG	CATTTGGAGGGAGGAGAAGGA
305+3	CCAAGGCGACCACATAATGTG	CGGTTCATTTGCTCTTCTTTGA
305+4	GGTACAATCATAGAAAAAGGGTACAAAGA	GGATTTGCTGAGTTCGCGTATT
306	CCCCAATCCCAATAGTTCGA	TGCGCCGCTCATACGA
306-1	GTCGTCGGTGAAGAAGATTACAGA	GCCCTGCCGGTCAAAAG
306-4	CGTCTTTGGCTTCATCTTTCATG	ATGCATAAAAGAGCTTGCCATAGA
306-6	AGCGAATCGTTATGCATTTTCA	CGCAACATAGAAGACCAATTTGAG
607	CGTGCGGCAGTATAAGTTCA	GCAGGATCGACCTGACTCTT
607-1	GGAGAGAATCTTACCTCAGAGTGC	GGGATCTTGAAAGTAAAACAGGTG
607-3	CTTTGTTATGGACCCGGAGA	CATCAAGATGGAATACTGTGACAA
607-6	GTTTCACCTCGTAGTCCCTCA	AACCAAATGCATTGCTTTATCA
809	CCGCGGAGTACGATTAAGAT	CGTGCTCTTGTTGTAGCTCGT
911	CGATAGGATTAGGGCAAATGA	CTTCACGTCGAAACTTTTTCTG
1212	GCTTCCAAGACGACAGTAATATG	ATAGATGTGCCTTCTGCAGCGAAAC
NTE1	TGACATTTCCGTAGCTTCATCA	GGATTCTTAACATTGTCGGTTGA
NegV	GCACTTAATTGGCGTAAGCTG	TCGCAGGAGCATATTTCGTA
ChrV	GGCTGTCAGAATATGGGGCCGTAGTA	CACCCCGAAGCTTTCACAATAC
GLT1	TTTGACCCCAGCACATGTTA	GGGTGTGGAGTTTGTGGTCT
305+11	CGCCCGACAGGGTAACA	GCGACAAACCTTTCGAAACAC

Supplementary Table 6: Antibodies used in this study

Antibody	Species	Dilution	References	Identifier
anti-CAPG2	Rabbit	1/2000	NB100-1813	Novus/Biotechne
anti-TOP2A	Rabbit	1/5000	ab52934	Abcam
anti-TOP2B	Mouse	1/7500	sc-25330	Santa Cruz Biotechnology
anti-TOP1	Rabbit	1/10000	ab109374	Abcam
anti-SMARCAL1	Mouse	1/500	sc-376377	Santa Cruz Biotechnology
anti-pCHK1 (Ser345)	Rabbit	1/1000	2348S	Cell Signaling/ozyme
anti-CHK1	Mouse	1/1000	2360	Cell Signaling/ozyme
anti-ACTIN	Mouse	1/5000	MAB1501	Millipore
anti-TUBULIN	Rat	1/3000	ab6161	Abcam
anti-GAPDH	Mouse	1/5000	MAB374	Merck/Millipore
anti-MYC	Mouse	1/2000	sc-40	Santa Cruz Biotechnology
anti-PK	Mouse	1/1000	MCA1360	Serotec
anti-AID	Mouse	1/1000	CAC-APC004AM	CosmoBio
anti-RPA70	Rabbit	1/500	ab79398	Abcam
anti-rat 488	Goat	1/100	A-11006	Invitrogen
anti-mouse 546	Goat	1/100	A-21123	Molecular Probes
anti-mouse 647	Goat	1/100	A-21241	Molecular Probes
anti rabbit 488	Goat	1/100	A11008	Life technologies
anti-BrdU clone B44	Mouse	1/200	347580	BD Biosciences
anti-BrdU clone BU1/75	Rat	1/300	ab6326	Abcam
