# Supplementary material for: Condensin and topoisomerases cooperate to relieve topological stress at stalled replication forks
Source: Nat Commun. 2026 May 8;17:6211. doi: 10.1038/s41467-026-72936-1 (PMC13369196; doi:10.1038/s41467-026-72936-1)
Supplement: Supplementary file 2 — Reporting Summary [file 41467_2026_72936_MOESM2_ESM.pdf]

nature portfolio

Corresponding author(s): armelle.lengronne@igh.cnrs.fr

Last updated by author(s): Apr 13, 2026

Reporting Summary

Nature Portfolio wishes to improve the reproducibility of the work that we publish. This form provides structure for consistency and transparency in reporting. For further information on Nature Portfolio policies, see our Editorial Policies and the Editorial Policy Checklist.

Statistics

For all statistical analyses, confirm that the following items are present in the figure legend, table legend, main text, or Methods section.

n/a	Confirmed	
☐	☒	The exact sample size (*n*) for each experimental group/condition, given as a discrete number and unit of measurement
☒	☐	A statement on whether measurements were taken from distinct samples or whether the same sample was measured repeatedly
☐	☒	The statistical test(s) used AND whether they are one- or two-sided<br>*Only common tests should be described solely by name; describe more complex techniques in the Methods section.*
☒	☐	A description of all covariates tested
☒	☐	A description of any assumptions or corrections, such as tests of normality and adjustment for multiple comparisons
☐	☒	A full description of the statistical parameters including central tendency (e.g. means) or other basic estimates (e.g. regression coefficient) AND variation (e.g. standard deviation) or associated estimates of uncertainty (e.g. confidence intervals)
☐	☒	For null hypothesis testing, the test statistic (e.g. *F*, *t*, *r*) with confidence intervals, effect sizes, degrees of freedom and *P* value noted<br>*Give P values as exact values whenever suitable.*
☒	☐	For Bayesian analysis, information on the choice of priors and Markov chain Monte Carlo settings
☒	☐	For hierarchical and complex designs, identification of the appropriate level for tests and full reporting of outcomes
☒	☐	Estimates of effect sizes (e.g. Cohen's *d*, Pearson's *r*), indicating how they were calculated

Our web collection on statistics for biologists contains articles on many of the points above.

Software and code

Policy information about availability of computer code

Data collection	ChIP-Seq and Copy Number variation data<br>Replication fork progression data
Data analysis	The genomic sequencing data were analyzed using the tools available on Galaxy (https://usegalaxy.org/).<br>The DNA fibers were measured by MetaMorph Microscopy Automation and Image Analysis Software (Molecular Devices) and statistical analysis was performed with Graph Pad Prism 8 (GraphPad Software).

For manuscripts utilizing custom algorithms or software that are central to the research but not yet described in published literature, software must be made available to editors and reviewers. We strongly encourage code deposition in a community repository (e.g. GitHub). See the Nature Portfolio guidelines for submitting code & software for further information.

Data

Policy information about availability of data

All manuscripts must include a data availability statement. This statement should provide the following information, where applicable:

- Accession codes, unique identifiers, or web links for publicly available datasets
- A description of any restrictions on data availability
- For clinical datasets or third party data, please ensure that the statement adheres to our policy

Source data are provided with this paper. The datasets generated during and/or analyzed during the current study are available from the corresponding authors.

accession code and web link for sequencing data: https://www.ncbi.nlm.nih.gov/geo/query/acc.cgi?acc=GSE280436
Token "inmrgukkhdabxsr" during the review process

Research involving human participants, their data, or biological material

Policy information about studies with human participants or human data. See also policy information about sex, gender (identity/presentation), and sexual orientation and race, ethnicity and racism.

Reporting on sex and gender	Non applicable
Reporting on race, ethnicity, or other socially relevant groupings	Non applicable
Population characteristics	Non applicable
Recruitment	Non applicable
Ethics oversight	Non applicable

Note that full information on the approval of the study protocol must also be provided in the manuscript.

Field-specific reporting

Please select the one below that is the best fit for your research. If you are not sure, read the appropriate sections before making your selection.

☒ Life sciences ☐ Behavioural & social sciences ☐ Ecological, evolutionary & environmental sciences

For a reference copy of the document with all sections, see nature.com/documents/nr-reporting-summary-flat.pdf

Life sciences study design

All studies must disclose on these points even when the disclosure is negative.

Sample size	Sample size was determined according to standards in the field. For DNA fiber spreading, at least 150 fibers were measured. Quantification of Mean Fluorescence Intensity (MFI) was performed on more than 400 cells.
Data exclusions	No data were excluded.
Replication	The experiments are performed at least in triplicate, and for DNA spreading analyses, most often three, four, or five times. Only the experiments involving electron microscopy were performed twice due to the complexity of this approach. We also used different human cell lines to ensure the validity of the results. Whenever possible, we also analyze the localization or the effect of the depletion of different factors within the same complex.
Randomization	Not relevant as no sample groups were used.
Blinding	Blinding was used for DNA fiber analysis.

Reporting for specific materials, systems and methods

We require information from authors about some types of materials, experimental systems and methods used in many studies. Here, indicate whether each material, system or method listed is relevant to your study. If you are not sure if a list item applies to your research, read the appropriate section before selecting a response.

Materials & experimental systems

n/a	Involved in the study	
☐	☒	Antibodies
☐	☒	Eukaryotic cell lines
☐	☐	Palaeontology and archaeology
☒	☐	Animals and other organisms
☒	☐	Clinical data
☒	☐	Dual use research of concern
☒	☐	Plants

Methods

n/a	Involved in the study	
☐	☒	ChIP-seq
☒	☐	Flow cytometry
☒	☐	MRI-based neuroimaging

Antibodies

Antibodies used

Rabbit anti-CAPG2 (Novus/Biotechne ; Cat #NB100-1813); Rabbit anti-TOP2A (Abcam; Cat #ab52934); Mouse anti-TOP2B (Santa Cruz Biotechnology; Cat # sc-25330); Rabbit anti-TOP1 (Abcam, Cat # ab109374); Mouse anti-SMARCAL1 (Santa Cruz Biotechnology; Cat # sc-376377); Rabbit anti-pCHK1 (S345) (Cell Signaling ; Cat #2348); Mouse anti-CHK1 (Cell Signaling ; Cat #2360); Rat anti-alpha-tubulin (Abcam; Cat #ab6161); Mouse anti-ACTIN (Millipore; Cat # MAB1501);Mouse anti-GAPDH (Merck/Millipore ; Cat # MAB374); Mouse anti-MYC (Santa Cruz Biotechnology ; Cat # sc-40); Mouse anti-PK (Serotec; Cat # MCA1360); Mouse anti-AID (CosmoBio; Cat # CAC-APC004AM); Rabbit anti-RPA70 (Abcam; Cat #ab79398); Mouse anti-BrdU clone B44 (BD Biosciences; Cat #347580); Rat anti-BrdU clone BU1/75 (Abcam; Cat #ab6326); Mouse autoanti-ssDNA supernatant (DSHB); Goat anti-rat 488 ; (Invitrogen; Cat # A-11006); Goat anti-mouse 546 ; (Molecular Probes; Cat # A-21123); Goat anti-mouse 647 ; (Molecular Probes ; Cat # A-21241); Goat anti rabbit 488; (Life technologies ; Cat # A11008)

Validation

All antibodies used in this manuscript have been previously validated in published studies.
Rabbit anti-CAPG2 (1/2000);Rabbit anti-TOP2A (1/5000);Mouse anti-TOP2B (1/7500); Rabbit anti-TOP1 (1/10000);Mouse anti-SMARCAL1 (1/500);Rabbit anti-pCHK1 (S345) (1/1000); Mouse anti-CHK1 (1/1000);Rat anti-alpha-tubulin (1/3000);Mouse anti-ACTIN (1/5000);Mouse anti-GAPDH (1/5000);Mouse anti-MYC (1/2000);Mouse anti-PK (1/1000);Mouse anti-AID (1/1000);Rabbit anti-RPA70 (1/500);Mouse anti-BrdU clone B44 (1/200); Rat anti-BrdU clone BU1/75 (1/300); Mouse autoanti-ssDNA supernatant (1/100); Goat anti-rat 488 ; (1/100);Goat anti-mouse 546 ; (1/100);Goat anti-mouse 647 ; (1/100);Goat anti rabbit 488; (1/100)

Anti-CAPG2 = https://www.novusbio.com/products/ncapg2-antibody_nb100-1813?srsltid=AfmBOoqH6BNuxyRzzDgDp2Kh4mYi-bNyKS28gDMwNWoYgXwGtuYjNPKo
Anti-TOP2A = https://www.abcam.com/en-us/products/primary-antibodies/topoisomerase-ii-alpha-antibody-ep1102y-ab52934
Anti-TOP2B = https://www.scbt.com/fr/p/topo-iibeta-antibody-h-8?srsltid=AfmBOoqGZId3c9a0O4qjpbXeTvnrnE01jLbCVwThpCYj7nUiHTnY0GcP
Anti-TOP1 = https://www.abcam.com/en-us/products/primary-antibodies/topoisomerase-i-antibody-epr5375-ab109374
Anti-SMARCAL1 = https://www.scbt.com/fr/p/smarcal1-antibody-a-2?srsltid=AfmBOopxgBfY4FUGFRX-xLO7rECJGwvHn5jq1kqJldjsW_cflOL2OnHk
anti-pCHK1 (Ser345) = https://www.cellsignal.com/products/primary-antibodies/phospho-chk1-ser345-133d3-rabbit-monoclonal-antibody/2348?srsltid=AfmBOopajMMa1zjUObaD0vb-h8JlpguG0irBbHDzJWHWyYl5nyppv9GV
Anti-CHK1 = https://www.cellsignal.com/products/primary-antibodies/chk1-2g1d5-mouse-monoclonal-antibody/2360?srsltid=AfmBOoqziucCHf3fx_go2fw1odM1WGgpyp70-1RYEiTpiR9DeM2AV7Ya
Anti-ACTIN = https://www.merckmillipore.com/SH/en/product/mm/mab1501
Anti-TUBULIN = https://www.abcam.com/en-us/products/primary-antibodies/tubulin-antibody-yol1-34-loading-control-ab6161
Anti-GAPDH = https://www.sigmaaldrich.com/ES/es/product/mm/mab374
Anti-MYC = https://www.scbt.com/fr/p/c-myc-antibody-9e10
Anti-PK = https://www.bio-rad-antibodies.com/monoclonal/viral-v5-tag-antibody-sv5-pk1-mca1360.html?f=purified
Anti-AID = https://www.cosmobiousa.com/products/anti-aid-tag-iaa17-protein-mab?srsltid=AfmBOorry8OfKehw2x1e4SapYZ3NFdLbVPYD-Nrrawc1_96JevoptV_O
Anti-RPA70 = https://www.abcam.com/en-us/products/primary-antibodies/rpa70-antibody-epr3472-ab79398
anti-rat 488 = https://www.thermofisher.com/antibody/product/Goat-anti-Rat-IgG-H-L-Cross-Adsorbed-Secondary-Antibody-Polyclonal/A-11006
anti-mouse 546 = https://www.thermofisher.com/antibody/product/Goat-anti-Mouse-IgG2a-Cross-Adsorbed-Secondary-Antibody-Polyclonal/A-21241
anti rabbit 488 = https://www.thermofisher.com/antibody/product/Goat-anti-Rabbit-IgG-H-L-Cross-Adsorbed-Secondary-Antibody-Polyclonal/A-11008
anti-BrdU clone B44 = https://www.bdbiosciences.com/en-us/products/reagents/flow-cytometry-reagents/clinical-discovery-research/single-color-antibodies-ruo-gmp/purified-mouse-anti-brdu.347580?tab=antibody_details
anti-BrdU clone BU1/75 = https://www.abcam.com/en-us/products/primary-antibodies/brdu-antibody-bu1-75-icr1-proliferation-marker-ab6326

Eukaryotic cell lines

Policy information about cell lines and Sex and Gender in Research

Cell line source(s)

Human cervical adenocarcinoma HeLa S3 (ATCC, CCL2-2) and osteosarcoma U2OS (ATCC HTB-86)

Authentication

None of the cell lines used were authenticated.

Mycoplasma contamination	Mycoplasma testing by qPCR was performed periodically to ensure that the cell lines are mycoplasma free.
Commonly misidentified lines (See ICLAC register)	No commonly misdefined cell lines were used in the study.

Palaeontology and Archaeology

Specimen provenance	Non applicable
Specimen deposition	Non applicable
Dating methods	Non applicable

☐ Tick this box to confirm that the raw and calibrated dates are available in the paper or in Supplementary Information.

Ethics oversight	Non applicable

Note that full information on the approval of the study protocol must also be provided in the manuscript.

Plants

Seed stocks	Non applicable
Novel plant genotypes	Non applicable
Authentication	Non applicable

ChIP-seq

Data deposition

☒ Confirm that both raw and final processed data have been deposited in a public database such as GEO.

☒ Confirm that you have deposited or provided access to graph files (e.g. BED files) for the called peaks.

Data access links *May remain private before publication.*	https://www.ncbi.nlm.nih.gov/geo/query/acc.cgi?acc=GSE280436
Files in database submission	GSM8597871 Input; GSM8597872 ChiP Brn1-PK in G1 in WT cells; GSM8597873 ChiP Brn1-PK in HU60 in WT cells;GSM8597874 ChiP Brn1-PK in HU60 in rad50delta cells;GSM8597875 IP Brn1-PK in HU60 in WT cells for time course;GSM8597876 Input Brn1-PK in HU60 in WT cells for time course;GSM8597877 IP Brn1-PK in HU90 in WT cells for time course;GSM8597878 Input Brn1-PK in HU90 in WT cells for time course;GSM8597879 IP Brn1-PK in HU120 in WT cells for time course;GSM8597880 Input Brn1-PK in HU120 in WT cells for time course;GSM8597881 genomic DNA in G1 in WT cells for time course;GSM8597882 genomic DNA in HU60 in WT cells for time course;GSM8597883 genomic DNA in HU90 in WT cells for time course;GSM8597884 genomic DNA in HU120 in WT cells for time course;
Genome browser session (e.g. UCSC)	We use Integrated Genome Browser (available at: https://bioviz.org/earlyAccessDownload.html).<br>BigWig (.bw) files deposited in the GEO database can be directly opened in IGB.

Methodology

Replicates	The replicates were processed by ChIP-qPCR. The time course performed during the S phase constitutes a replicate in itself. The ChIP-Seq of different condensin subunits (Brn1, Smc4) also indirectly serves as a replicate.

Sequencing depth

File name	Total reads	Reads mapped	Average Read length	
GSM8597871	34114689,00	33011808,00	51,00	Single-end
GSM8597872	46122117,00	43107449,00	51,00	Single-end
GSM8597873	37690229,00	33778353,00	51,00	Single-end
GSM8597874	35333807,00	32461870,00	51,00	Single-end
GSM8597875	22834488,00		140,00	Paired-end
GSM8597876	21886622,00	19259590,00	141,00	Paired-end
GSM8597877	14696208,00	11968432,00	139,00	Paired-end
GSM8597878	22008398,00	19100028,00	141,00	Paired-end

GSM8597879	18230068,00	16462360,00	139,00	Paired-end
GSM8597880	24165614,00	22353070,00	140,00	Paired-end
GSM8597881	20225012,00	19473424,00	141,00	Paired-end
GSM8597882	22334914,00	21541562,00	142,00	Paired-end
GSM8597883	25812294,00	24850846,00	142,00	Paired-end
GSM8597884	23402122,00	22490632,00	142,00	Paired-end

Antibodies

Mouse anti-PK (Serotec; Cat # MCA1360)

Peak calling parameters

N/A

Data quality

The analyses were performed on Galaxy (https://usegalaxy.eu/).
The reads were aligned to the Saccharomyces cerevisiae reference genome (sacCER3) using Bowtie2. The reads were then trimmed to remove adapters (Trim Galore tool), duplicates were removed (Markduplicates tool), and the Bamcompare tool was used to generate BigWig files. The FastQC and Samtools tools provide data quality control and coverage, respectively.

Software

The analyses were performed on Galaxy (https://usegalaxy.eu/).
