# Supplementary material for: Condensin and topoisomerases cooperate to relieve topological stress at stalled replication forks
Source: Nat Commun. 2026 May 8;17:6211. doi: 10.1038/s41467-026-72936-1 (PMC13369196; doi:10.1038/s41467-026-72936-1)
Supplement: Supplementary file 3 — Transparent Peer Review file [file 41467_2026_72936_MOESM3_ESM.pdf]

nature portfolio

Peer Review File

Condensin and topoisomerases cooperate to relieve topological stress at stalled replication forks

Corresponding Author: Dr Armelle Lengronne

This file contains all reviewer reports in order by version, followed by all author rebuttals in order by version.

Version 0:

Reviewer comments:

Reviewer #1

(Remarks to the Author)
Da Mota et al, investigated the replicative functions of condensin during replication stress and the interplay with topoisomerases. These functions have been studied in parallel in yeast and human cells. Using well-established genomic approaches and genetics, the authors established that, in budding yeast, condensin is recruited at stalled forks in an MRX and chromatin remodelling-manner. Moreover, using DNA fiber assays, condensin was found to promote replication restart in a complex interplay with topoisomerases. In human cells, using DNA fibers and electronic microscopy, the authors established that condensin II, but not I, is required to promote fork reversal and resection, acting together with TOP2A. When performing “epistasis analysis”, complex relationships between condensin II, TOP1 and TOP2A, and fork remodelers are identified. Finally, the authors found that condensin prevents single stranded DNA upon replication stress. The authors proposed, in a model, that condensin II and topoisomerases act together at different stages to handle topological constraints at stalled forks to allow fork reversal and restart. Overall, the results in this paper are properly displayed and represented. Despite rooms for alternative interpretations, the model proposed is convincing. These discoveries push the boundaries of our understanding of the complex topological transactions occurring at stalled forks. These insights will be of major interest for a broad scientific community working on genome stability. Nonetheless, there are few points that the authors must address to strengthen and clarify their conclusion.

Major comments

1. Figure 1: the authors nicely showed that yeast condensin is recruited at HU-stalled forks using a combination of genomic assays. Can the authors strengthen their data by showing that condensin is also recruited at replication fork during normal S-phase, as reported for human condensin ?
2. Figure 2a: the authors showed that condensin is required to sustain fork progression upon HU treatment. Did the author check that smc2-8 cells enter in S-phase with the same kinetics as WT cells ? A delayed G1/S transition may explain the apparent decrease in fork progression.
3. Figure 2d and S2h, page 8. The authors concluded that “simultaneous depletion of Smc4 and Top2 had no additional effect on fork recovery when compared with depletion of these proteins individually”. Fig2d seems to indicate an additive effect (but no statistics were provided) whereas FigS2h seems to indicate no additive effect. Can the authors clarify this point ?
4. Figure 2c, page 8. The authors showed that top1 depletion restores fork restart in the absence of condensin. Does top1 depletion rescue the sensitivity to MMS and HU of smc2-8 or SMC4-AID cells. This would support that sensitivity to replication stress caused by defect in condensin is caused by defective fork restart.
5. Figure 4e-g and page 12: The authors concluded that “in the absence of condensin II, SMARCAL1 is dispensable for fork reversal”. Same conclusion was made with Fbh1 (FigS4g). It is unclear, in that context, which pathway drives fork reversal that no longer requires condensin II to handle topological constraints and that allows resection and fork restart ? Would it be possible, that in the absence of fork remodelers such as Fbh1 and SMARCAL1, condensin II inhibits alternative fork reversal pathways ? Moreover, this pathway may be prevented by Top1 activity as well (Fig 5c-e). If relevant, can the authors discuss this point in the discussion part ?
6. Figure 6: The authors monitored the amount of RPA-coated ssDNA at HU-arrested forks in budding yeast by ChIP-qPCR (Fig6a) and in human cells using IF (Fig6b). They observed an increase in RPA-coated ssDNA in the absence of condensin and TOP2A, despite defective fork resection. An increase in RPA-coated ssDNA was also observed in the absence of condensin and TOP1, a situation in which fork reversal and resection occur (Fig5c-d). Therefore, it is difficult to appreciate

what this RPA-coated ssDNA assay reflects. If the authors want to claim that condensin and topoisomerases act in the re-annealing of parental DNA (page 14, lines 379-380, page 18 lines 503-507), they must strengthen their data by addressing this directly, using native Brdu labeling assays or PLA between native Brdu and RPA if amplification of the signal is needed. This point appears as the sole weakness of the paper.

Minor comments

7. Page 5: what are the mutations corresponding to smc2-8 ?
8. Figure S1a and 1a: smc2-8 and SMC4-AID exhibit very mild sensitivity to MMS. To what extent SMC4 is properly depleted upon auxin addition. Was sensitivity to CPT assessed ?
9. Fig 1c, middle panel. The Brn1 peaks at 90 and 120 ‘ are of lower amplitude than the one at 60’, and thus, not really convincing. Can the authors comment on this ?
10. page 6, Fig1e and f: “Brn1 was located behind HU-arrested forks”. As reported by the authors themselves, forks are not arrested upon HU treatment, but are stalled/slowed down.
11. Fig S1 legend: “rad50D” needs correction.
12. page 8: FigS2i-j are introduced before FigS2g-h.
13. Fig S2 d-e. The authors concluded that condensin (Ycg1) is dispensable for normal fork progression. However, the experimental setup of panel e is different from experimental setup of panel d (defect upon HU treatment): Edu was incorporated 20 min post auxin addition, a time point at which Ycg1 is not depleted, according to the western-blot provided ? Can the authors confirm that Ycg1 was indeed depleted at the time Edu was added ?
14. Fig3a legend. What is LFQ intensity ? what does it reflect/quantify ?
15. Figure 3c: Are the authors sure that the western-blots are in the right orientation ?
16. FigS3 legend: *: p<0.1 . I guess it should be 0.01 ? Same mistakes in other figure legends.
17. FigS3a: CldU track length is significantly different between siCAPG and siCAPG2. Therefore, one could expect that CldU track length is significantly different between siCtrl and siCAPG2. Is this the case ? Does it mean that fork speed is increased upon CAPG2 depletion ?.
18. Throughout the paper, it is not easy to understand in which cell lines the experiments have been done. It would help if the authors could mention these experimental details directly on the figure when appropriated.
19. Page 13, lines 367-368: the authors concluded that “SMARCAL1 was required to promote fork resection in the absence of TOP2A” (Fig5f and S5m). Was fork slowing affected in the absence of both TOP2A and SMARCAL1 ? In addition, since fork resection requires TOP2A (Fig5b), why do the authors not conclude that TOP2A and SMARCAL1 act in the same pathway to promote fork reversal and resection ?
20. Statistics are missing on numerous figures (Fig1e-f, S1c, Fig 2, and many others).

Reviewer #2

(Remarks to the Author)
I co-reviewed this manuscript with one of the reviewers who provided the listed reports. This is part of the Nature Communications initiative to facilitate training in peer review and to provide appropriate recognition for Early Career Researchers who co-review manuscripts.

Reviewer #3

(Remarks to the Author)
The manuscript by Lengronne and colleagues reports the essential role of condensin in relieving topological stress accumulated at stalled replication forks. The authors provide evidence that the (sole) condensin complex in budding yeast and the condensin II complex in human cells do this job, and speculate how two different types of topoisomerases, topoisomerase I and topoisomerase II, differentially cooperate with the condensins. They also show that condensin II and SMARCAL1 promote fork restart through different mechanisms.

Despite the accumulating evidence, the question of exactly how condensins contributes to genome stability outside of mitosis remains elusive. The current manuscript attempts to address this important question by using budding yeast and human cells. The experiments reported here are well executed and will be an excellent addition to the literature. The following issues need to be addressed before it can be considered for publication.

Specific comments:
(1) It is not clear why the authors used the YCG1-AID strain in the experiment described in lines 160-174. This is not trivial, because a previous study in fission yeast used a kleisin mutant (cnd2-1) and reached a different conclusion from that reported here.
(2) The description that “condensin-mediated DNA overwinding may be crucial for chromosome decatenation by topoisomerase II” (lines 206-207) may need to be rephrased. For example, Ref 34 reports negative supercoiling, not overwinding, of DNA induced by budding yeast condensin. This reviewer recognizes that the issue discussed here is not easily resolved, but the current description is misleading and needs to be corrected.
(3) Figure 3 provides evidence that Smc2 and Smc4 travel with replication forks. It would be nice to show that condensin II-specific subunits, but not condensin I-specific subunits, behave accordingly.
(4) Similarly, it is very important to demostrate that the essential role in fork restart is ascribed to condensin II, but not condensin I. For example, siCAPG is missing from Figure 3d, and the text is generally inadequate to explain this important point.
(5) The depletion experiments in human cells reported in the current manuscript use RNA interference, which inevitably

requires a long depletion time (48 hr). The authors should discuss the potential drawbackd of this technique, which is becoming less popular in the field.
(6) The authors conclude that “condensin acts with topoisomerase I at reversed forks to convert positively supercoiled DNA into structures that are subsequently relaxed by topoisomerase II” (line 45-; line 410-). It is not very clear what is meant by “structures that are subsequently relaxed by topoisomerase II” means. Do they correspond to positive plectomenes as shown in Figure 7? The authors need to be more explicit on this point.
(7) “The activity would differ from its mitotic role….” (line 477). This reviewer understands the logic here, but are the mitotic and interphase activities discussed here indeed mechanistically different? Is there an explanation that possibly integrates the mitotic and interphase activities discussed here? The authors may want to revisit this point and possibly rephrase it.

Reviewer #4

(Remarks to the Author)
The study by Da Mota et al focuses on the functions of condensin and DNA topoisomerases during replication stress in budding yeast and human cells. Their initial results show that condensin is enriched at early replication origins in budding yeast cells cultured in hydroxyurea (HU). The enrichment of condensin around stalled replication forks requires Rad50, Gcn5 and Chd1.

The authors then allow cells to incorporate BrdU under HU conditions and perform DNA combing in the absence and presence of condensin subunits. The results show that in the absence of condensin function, shorter BrdU-DNA tracks are found following prolonged incubation in HU-media. The authors speculate that this is due to defects in fork restart in the absence of condensin function, and design an experiment to monitor fork restart following a pulse of MMS. Also, during the recovery from MMS, the absence of condensin function leads to shorter labelled DNA tracks.

Interestingly, the simultaneous depletion of Top1 rescues the short labelled DNA tracks in the absence of condensin function, whereas simultaneous depletion of Top2 leads to even shorter labelled DNA tracks than after either condensin or Top2 depletion alone (Fig. 2d).

The authors then analyze if condensin acts at replication forks in human cells. They find that condensin II promotes replication fork restart at low concentration of HU (Fig. 3d).

Unlike in yeast, at high concentration HU in human cells, knockdown of condensin II resulted in faster replication speed than in control cells (Fig. 3e). The authors speculate that condensin II might be required for slowing the DNA replication in human cells, in response to high HU concentrations.

The authors then aim to analyze for resectioning by first subjecting cells to consecutive pulses of IdU and CldU, and then adding low concentration of HU, which may induce fork resectioning and thereby shortening of the later CldU track. This appears to occur in control cells following HU treatment as the CldU/IdU ratio decreases. However, in condensin II (but not condensin I) depletion condition the CldU/IdU ratio is not altered by HU addition (Fig. 3f). The author states that this indicates that condensin II is essential for fork resectioning in HU conditions.

Condensin II is then shown to promote fork reversal by direct visualization of replication intermediates by electron microscopy (Fig. 4b). The interplay between condensin II and DNA topoisomerases or SMARCAL1 is then investigated.

The authors then switch back to yeast, and unlike previous experiments, decide to use Top1 deleted cells to monitor RPA enrichment around early firing origins. They find that in Top1 deleted cells high enrichment of RPA is detected, which is not altered when condensin is depleted.

In human cells, however, double depletion of condensin II and Top1 leads to considerably higher chromatin-bound RPA.

The manuscript ends with an attempt to present a model that encompasses condensin’s and DNA topoisomerases’ functions at stalled replication forks.

The amount of data presented in the manuscript is extensive, however, several important controls are missing. The amount of data presented, together with the differences in action between yeast condensin and condensin II in human cells, makes it difficult to present a clear and logical model in the end. In addition, throughout the manuscript, the authors speculate about the supercoiling state behind and ahead of stalled replication forks in a manner that does not seem reasonable.

The main points that need to be are addressed are:

1)
How are the authors convinced that the effects of condensin and topoisomerase depletion stems from their function in S-phase?
In yeast, it could be that the tagging of condensin subunits or DNA topoisomerases makes them partially non-functional and therefore initiates S-phase with some linguiring problems.
The authors should perform experiments in the absence of auxin to show that this is not the case.
The authors instead express WT SMC4 in the SMC4-AID strain. What is this a control for?

In human cells,
Are the authors certain that the 48 hour depletion of condensin II does not cause additional effects which affects the cells ability to respond to HU?
The authors should perform experiments using AID-tagged condensin II (+/- auxin) following release from a G1/S-block to ensure that the detected findings stems from condensin II's function in S-phase.

2)
Throughout the manuscript, the authors speculate about the DNA supercoiling state surrounding stalled replication forks, although no direct evidence is presented. The authors should consider to substantially reduce the amount of speculation, and address the questions below.
For example,
"Our findings suggest that condensin acts with topoisomerase I at reversed forks to convert positively supercoiled DNA into structures that are subsequently relaxed by topoisomerase 2, allowing the fork to resume replication."

How would Top1 convert positively supercoiled DNA into structures that are relaxed by Top2?
Top1 relaxes positively supercoiled DNA.

"The negative superhelical tension generated at reversed forks by reannealing of parental DNA strands is also a good substrate for TOP1."

How and where is the negative superhelical tension generated by reannealing the parental strands? Negative superhelical tension is created by unwinding double-stranded DNA.

"Since the positive torsional stress that accumulates ahead of the fork during DNA replication and drives fork reversal is resolved by TOP2A6,7, we investigated whether condensin II collaborates with TOP2A in fork reversal by assaying fork slowdown and fork resection as indicators of fork reversal in cells depleted of CAPG2, TOP2A or both proteins."

The suggestion that positive torsional stress that accumulates ahead of the fork during DNA replication drives fork reversal is a speculation, and should be referred to as such.

In the legend of Fig. 7, the authors speculate that:
"a Elongation of DNA replication generates positive supercoils (Sc+) in front of the forks that are normally relaxed by TOP1 and precatenanes behind the fork that are resolved by TOP2. b Upon fork arrest, Sc+ can be transferred behind the fork via a process called fork reversal, in which nascent DNA strands reanneal to form a regressed arm."

If positive supercoils are transferred behind the replication fork, the structure that is created behind the forks would be pre-catenanes, not positive supercoiling.
Also, since fork reversal (might) occur during HU treatment, when replication forks move very slowly, is it reasonable to consider that any substantial positive supercoiling occurs ahead of the forks under these conditions?
The author could test this by depleting Top1 and Top2 in HU-conditions to see if replication forks are stopped.

Additional points,

1)
Why is Top1 deletion used in Fig. 6, when Top1-AID was used in Fig 2. It makes it difficult to compare the results. The authors should perform the RPA enrichment analysis in TOP1-AID cells with or without auxin.

2)
The authors reach the conclusion that the simultaneous depletion of Smc4 and Top2 had no additional effect on EdU track length following MMS exposure.
"By contrast, simultaneous depletion of Smc4 and Top2 had no additional effect on fork recovery when compared with depletion of these proteins individually, indicating that condensin and Top2 may function in a common pathway required for efficient fork restart following replication stress"

However, in Fig. 2d there is an additional effect. Can the authors explain their reasoning?

3)
Non-calibrated, non-quantitative ChIP-seq is presented in the manuscript. To validate the findings, quantitative ChIP-qPCR experiments are presented alongside.
However, normalizing the data to an unreplicated region makes it impossible for the reader to appreciate the quality of the ChIP-experiment and understand the signal-to-noise ratio. This refers to the data in Fig. 1e, 1f and S1f. This is especially important since condensin is enriched in certain unreplicated regions.

Fig. 6a and S6a also show ChIP-qPCR data normalized to unreplicated regions. Also, these data should be presented without normalization so that the readers can assess the quality and reproducibility of the ChIP-qPCR experiment.

4)
In the absence of HU, no effect on replication is detected when condensin is inactivated. However, it is not shown that condensin is absent when the EdU is added (S2e). Can the lack of replication slowdown be due to the short depletion of condensin under these conditions?
Perhaps smc2-8 be analyzed to avoid this risk?

5)
What is the benefit of including Mirin? It does result in any difference.

Version 1:

Reviewer comments:

Reviewer #1

(Remarks to the Author)
The authors have carefully and successfully addressed the comments raised. Despite the origin of RPA-coated ssDNA remaining unclear, the authors have done their best to clarify this point in the revised version by showing that it contributes to cell sensitivity to replication stress in the absence of functional condensin.

Attention could be given to few mistakes:

Line 121: 'HU-arrested forks' could be replaced by "HU-stalled forks".
Lines 218-218: "rfa1(G77A)" and figure 2, the allele nomenclature may be changed change. rfa1-G77E or rfa1G77E as originally published in ref 31.
Line 324: "failed to undergo nascent DNA under these conditions", the word resection is likely missing.
Line 413: "SMARCAL1 was dispensable for fork slowing and resection TOP1-deficient cells" In TOP1…
Line 529 "with a role for these factors in of parental DNA strand reannealing" remove of ?

Reviewer #2

(Remarks to the Author)
I co-reviewed this manuscript with one of the reviewers who provided the listed reports. This is part of the Nature Communications initiative to facilitate training in peer review and to provide appropriate recognition for Early Career Researchers who co-review manuscripts.

Reviewer #3

(Remarks to the Author)
The revised manuscript has successfully addressed the concerns I raised in the first round of reviews. While I am still wondering why fission yeast behaves differently from budding yeast and humans, this issue may be beyond the scope of the present work. I would simply suggest rephrasing the sentence in the summary from "we show in yeast and human cells" to we show in budding yeast and human cells" (line 43).

Reviewer #4

(Remarks to the Author)
I thank the authors for the detailed rebuttal. The new model presented in Figure 7 focuses on the results from human cells. Would it be possible for the authors to also incorporate the findings from budding yeast?

For example, the rfa1(G77E) mutation fully suppresses the HU sensitivity of smc2-8 cells. This new finding is striking and interesting; however, it is not currently considered in the final model. Could the authors clarify their interpretation of this result?

In relation to the above, RPA levels at stalled forks do not appear to be directly linked to fork recovery, since the top1Δ SMC4-AID double mutant clearly shows higher RPA levels than WT but displays no defect in fork restart.

This further strengthens the need to clarify why the rfa1(G77E) mutation fully suppresses the condensin mutant's HU sensitivity.

The authors should test whether the rfa1(G77E) allele rescues the smc2-8 defect in fork restart. This would help clarify the role of RPA-coated ssDNA in condensin activity at stalled replication forks.

Minor point:
I would recommend softening the statements in the Introduction suggesting that fork reversal is initiated by the accumulation

of positive supercoiling, and that extension of the reversed arm generates positive supercoiling along sister chromatids, as these interpretations are speculative.

Open Access This Peer Review File is licensed under a Creative Commons Attribution 4.0 International License, which permits use, sharing, adaptation, distribution and reproduction in any medium or format, as long as you give appropriate credit to the original author(s) and the source, provide a link to the Creative Commons license, and indicate if changes were made.
In cases where reviewers are anonymous, credit should be given to 'Anonymous Referee' and the source.
The images or other third party material in this Peer Review File are included in the article's Creative Commons license, unless indicated otherwise in a credit line to the material. If material is not included in the article's Creative Commons license and your intended use is not permitted by statutory regulation or exceeds the permitted use, you will need to obtain permission directly from the copyright holder.
To view a copy of this license, visit https://creativecommons.org/licenses/by/4.0/

REVIEWER COMMENTS

Reviewer #1 (Remarks to the Author):

Da Mota et al, investigated the replicative functions of condensin during replication stress and the interplay with topoisomerases. These functions have been studied in parallel in yeast and human cells. Using well-established genomic approaches and genetics, the authors established that, in budding yeast, condensin is recruited at stalled forks in an MRX and chromatin remodeling-manner. Moreover, using DNA fiber assays, condensin was found to promote replication restart in a complex interplay with topoisomerases. In human cells, using DNA fibers and electronic microscopy, the authors established that condensin II, but not I, is required to promote fork reversal and resection, acting together with TOP2A. When performing "epistasis analysis", complex relationships between condensin II, TOP1 and TOP2A, and fork remodelers are identified. Finally, the authors found that condensin prevents single stranded DNA upon replication stress. The authors proposed, in a model, that condensin II and topoisomerases act together at different stages to handle topological constraints at stalled forks to allow fork reversal and restart. Overall, the results in this paper are properly displayed and represented. Despite rooms for alternative interpretations, the model proposed is convincing. These discoveries push the boundaries of our understanding of the complex

topological transactions occurring at stalled forks. These insights will be of major interest for a broad scientific community working on genome stability. Nonetheless, there are few points that the authors must address to strengthen and clarify their conclusion.

Major comments

1. Figure 1: the authors nicely showed that yeast condensin is recruited at HU-stalled forks using a combination of genomic assays. Can the authors strengthen their data by showing that condensin is also recruited at replication fork during normal S-phase, as reported for human condensin?

Thank you for the comment. To address whether condensin is also recruited to replication forks under normal growth conditions, we arrested cells in G_1 with α-factor, then released them into S phase for the indicated times, with or without 200 mM HU. Enrichment of the condensin subunit Brn1-PK_9 at the early origins *ARS305* and *ARS306* was analyzed by ChIP-qPCR. Although we detected again a significant Brn1 enrichment at HU-arrested forks, the signal was barely above background levels in the absence of HU (see **new Supplementary Fig. 1d**), and did not correlate with fork progression as measured by DNA copy number changes (see **new Supplementary Fig. 1e**). These data indicate that, unlike in human cells, condensin is not markedly enriched at replication forks in the absence of replication stress, or too dynamic to be detected in a cell population.

2. Figure 2a: the authors showed that condensin is required to sustain fork progression upon HU treatment. Did the author check that smc2-8 cells enter in S-phase with the same kinetics as WT cells? A delayed G1/S transition may explain the apparent decrease in fork progression.

This is indeed an important control that was missing in the original version of the manuscript. To address the possibility that the kinetics of origin activation could be altered in *smc2-8* cells due to the temperature shift to 37°C, we monitored origin activation upon entry into S phase by qPCR to measure changes in DNA copy number at a variety of early and late replication origins. As illustrated in the **new Supplementary Fig. 2d**, we detected no difference in the activation of the early origins *ARS305*, *ARS306* and *ARS607* relative to WT cells. Moreover, the late origins *ARS809*, *ARS911* and *ARS1212* were properly repressed, which is usually not the case when the kinetics of early origin firing is altered. A similar pattern of origin activation is seen in pMET-YCG1-AID cells after AID-induced condensin depletion (**Supplementary Fig. 2b**).

3. Figure 2d and S2h, page 8. The authors concluded that “simultaneous depletion of Smc4 and Top2 had no additional effect on fork recovery when compared with depletion of these proteins individually”. Fig2d seems to indicate an additive effect (but no statistics were provided) whereas FigS2h seems to indicate no additive effect. Can the authors clarify this point?

Thank you for pointing this out. We have now conducted an extensive analysis comparing differences both within individual experiments and between the means of biological replicates (n=3). While small differences between samples can appear statistically significant due to the large number of data points (n>100), the differences between the means of biological replicates are not statistically significant (**new Fig. 3b**). We have revised the text in the Methods section to clarify this apparent discrepancy. A table with all median, mean and p-values is also provided (**new Table 1**).

4. Figure 2c, page 8. The authors showed that top1 depletion restores fork restart in the absence of condensin. Does top1 depletion rescue the sensitivity to MMS and HU of smc2-8 or SMC4-AID cells.

This would support that sensitivity to replication stress caused by defect in condensin is caused by defective fork restart.

Thank you for this insightful comment. We now show that *top1Δ* partially rescues the growth of *smc2-8* mutants in the presence of 100 mM HU or 0.033% MMS (**new Fig. 3a**), which is consistent with the fact that Top1 loss restores fork restart in the absence of condensin.

5. Figure 4e-g and page 12: The authors concluded that "in the absence of condensin II, SMARCAL1 is dispensable for fork reversal". Same conclusion was made with Fbh1 (FigS4g). It is unclear, in that context, which pathway drives fork reversal that no longer requires condensin II to handle topological constraints and that allows resection and fork restart? Would it be possible, that in the absence of fork remodelers such as Fbh1 and SMARCAL1, condensin II inhibits alternative fork reversal pathways? Moreover, this pathway may be prevented by Top1 activity as well (Fig 5c-e). If relevant, can the authors discuss this point in the discussion part?

We favor a model in which condensin II and TOP1 together generate a positively supercoiled plectoneme that serves as a topological barrier to fork reversal unless resolved by TOPIIA. We have revised the text to better explain that when this barrier does not form, the requirement for redundant translocase activities appears reduced, allowing fork reversal even in the absence of SMARCAL1 or FBH1. To our knowledge, this represents the first instance of fork reversal occurring without SMARCAL1.

6. Figure 6: The authors monitored the amount of RPA-coated ssDNA at HU-arrested forks in budding yeast by ChIP-qPCR (Fig6a) and in human cells using IF (Fig6b). They observed an increase in RPA-coated ssDNA in the absence of condensin and TOP2A, despite defective fork resection. An increase in RPA-coated ssDNA was also observed in the absence of condensin and TOP1, a situation in which fork reversal and resection occur (Fig5c-d). Therefore, it is difficult to appreciate what this RPA-coated ssDNA assay reflects. If the authors want to claim that condensin and topoisomerases act in the re-annealing of parental DNA (page 14, lines 379-380, page 18 lines 503-507), they must strengthen their data by addressing this directly, using native BrdU labeling assays or PLA between native BrdU and RPA if amplification of the signal is needed. This point appears as the sole weakness of the paper.

We observed increased RPA-coated ssDNA in yeast and chromatin-bound RPA in human cells lacking condensin, which is counterintuitive given that these cells are defective in fork resection. We initially proposed that ssDNA accumulation resulted from impaired reannealing of parental DNA strands. However, as the reviewer correctly noted, immunofluorescence cannot distinguish whether RPA is bound to parental or nascent DNA. To address this limitation, we labeled parental DNA with BrdU and performed proximity ligation assay (PLA) between BrdU and RPA without DNA denaturation, as requested by the reviewer. Unfortunately, these experiments did not yield reproducible results despite multiple attempts, likely because the antibodies used were not compatible with PLA. Furthermore, even if technically successful, this approach would not definitely demonstrate that ssDNA on parental strands localizes near arrested forks rather than elsewhere in the genome.

We therefore performed a complementary experiment to assess the physiological impact of excessive RPA-bound ssDNA in condensin mutants exposed to replication stress. Specifically, we tested whether reducing RPA's affinity for ssDNA could enhance their tolerance to replication stress. To this end, we used the *rfa1-G77E* allele, which binds ssDNA less efficiently (Murayama et al., Cell 2018). Remarkably, this allele improved the growth of the *smc2-8* mutant under normal conditions and restored its growth

on hydroxyurea at the semi-permissive temperature (**new Fig. 2d**). Together with ChIP-qPCR data (**Fig. 2c**), these findings indicate that condensin prevents the accumulation of excess ssDNA during replication stress. As the precise genomic localization of this ssDNA remains to be established, we have toned down this aspect in our model.

Minor comments

7. Page 5: what are the mutations corresponding to smc2-8?

The *smc2-8* mutation corresponds to the insertion of four perfect repeats and two imperfect repeats of the sequence MEQKLISEEDLNE, which corresponds to the canonical Myc epitope sequence EQKLISEEDL (10 amino acids) with additional flanking sequences. This sequence is inserted close to the hinge region of Smc2, interfering with the function of the condensin complex (Manuel Mendoza, IGBMC, personal communication). This information is now indicated in the Methods section.

8. Figure S1a and 1a: smc2-8 and SMC4-AID exhibit very mild sensitivity to MMS. To what extent SMC4 is properly depleted upon auxin addition. Was sensitivity to CPT assessed?

In this experiment, we intentionally use a low dose of auxin on MMS plates to achieve partial condensin depletion as higher doses cause cell death and prevent any growth on the plates. Sensitivity to CPT was also examined for the *smc2-8* allele at a semi-permissive temperature (30°C), alongside sensitivity to HU and zeocin, the latter inducing DNA double-strand breaks. While the *smc2-8* mutant shows a clear sensitivity to HU and zeocin, no evident sensitivity to CPT was observed (see below).

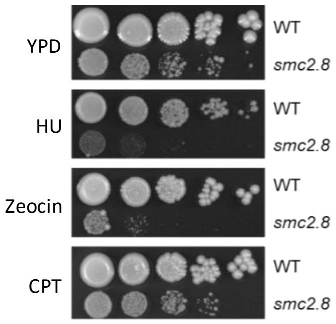

Figure I for reviewers: Wild-type (WT) and *smc2-8* cells were grown for 4 days on YPD plates containing either no drug (YPD), 100 mM hydroxyurea (HU), 10 µM Zeocin or 5 µM camptothecin (CPT) at the semi-permissive temperature of 30°C.

9. Fig 1c, middle panel. The Brn1 peaks at 90 and 120' are of lower amplitude than the one at 60', and thus, not really convincing. Can the authors comment on this?

The flattening of the Brn1 peaks observed at 90- and 120-min results from ongoing fork progression in the presence of HU, causing replication forks to spread over a longer distance. This, in turn, leads to a broader distribution of the Brn1 signal. This is now addressed in the manuscript.

10. page 6, Fig1e and f: "Brn1 was located behind HU-arrested forks". As reported by the authors themselves, forks are not arrested upon HU treatment, but are stalled/slowed down.

Thanks for pointing this out. We have now systematically replaced HU-arrested forks with HU-stalled or HU-treated forks in the manuscript.

11. Fig S1 legend: “rad50D” needs correction.

Done

12. page 8: FigS2i-j are introduced before FigS2g-h.

Exchange done in the figures

13. Fig S2 d-e. The authors concluded that condensin (Ycg1) is dispensable for normal fork progression. However, the experimental setup of panel e is different from experimental setup of panel d (defect upon HU treatment): Edu was incorporated 20 min post auxin addition, a time point at which Ycg1 is not depleted, according to the western-blot provided? Can the authors confirm that Ycg1 was indeed depleted at the time Edu was added?

Ycg1 was indeed depleted at the time EdU addition. This now displayed in a **new Supplementary Figure 2f**.

14. Fig3a legend. What is LFQ intensity? what does it reflect/quantify?

LFQ stands for « Label-Free Quantification ». This is now indicated in the figure legend.

15. Figure 3c: Are the authors sure that the western-blots are in the right orientation?

Yes, we have double-checked that the Western blot signals are correctly oriented in this panel (**now 4c**).

16. FigS3 legend: *: p<0.1 . I guess it should be 0.01 ? Same mistakes in other figure legends.

Thank you for pointing this out. The correct notation is *: p<0.05. This correction has been applied to the other figure legends as well.

17. FigS3a: CldU track length is significantly different between siCAPG and siCAPG2. Therefore, one could expect that CldU track length is significantly different between siCtrl and siCAPG2. Is this the case? Does it mean that fork speed is increased upon CAPG2 depletion?

We have performed an extensive analysis comparing differences within individual experiments and between the means of biological replicates (n=4) across superplots. This analysis shows that the small differences (p<0.05) within individual experiments (**Supplementary Fig. 4a**) observed between cells lacking CAPG or CAPG2 and Ctrl cells are not statistically significant when comparing the means of biological replicates (**new Fig. 4b**).

18. Throughout the paper, it is not easy to understand in which cell lines the experiments have been done. It would help if the authors could mention these experimental details directly on the figure when appropriated.

All cell lines are now identified in the figure legends.

19. Page 13, lines 367-368: the authors concluded that “SMARCAL1 was required to promote fork resection in the absence of TOP2A” (Fig5f and S5m). Was fork slowing affected in the absence of both TOP2A and SMARCAL1? In addition, since fork resection requires TOP2A (Fig. 5b), why do the authors not conclude that TOP2A and SMARCAL1 act in the same pathway to promote fork reversal and resection?

The fact that TOP2A and SMARCAL1 act in the same pathway to promote fork reversal and resection was extensively studied by Tian *et al.* 2021. This is now mentioned in this section.

20. Statistics are missing on numerous figures (Fig1e-f, S1c, Fig 2, and many others).

We now provide statistics for all ChIP-qPCR experiments.

Reviewer #2 (Remarks to the Author):

I co-reviewed this manuscript with one of the reviewers who provided the listed reports. This is part of the Nature Communications initiative to facilitate training in peer review and to provide appropriate recognition for Early Career Researchers who co-review manuscripts.

Reviewer #3 (Remarks to the Author):

The manuscript by Lengronne and colleagues reports the essential role of condensin in relieving topological stress accumulated at stalled replication forks. The authors provide evidence that the (sole) condensin complex in budding yeast and the condensin II complex in human cells do this job, and speculate how two different types of topoisomerases, topoisomerase I and topoisomerase II, differentially cooperate with the condensins. They also show that condensin II and SMARCAL1 promote fork restart through different mechanisms.

Despite the accumulating evidence, the question of exactly how condensins contributes to genome stability outside of mitosis remains elusive. The current manuscript attempts to address this important question by using budding yeast and human cells. The experiments reported here are well executed and will be an excellent addition to the literature. The following issues need to be addressed before it can be considered for publication.

Specific comments:
(1) It is not clear why the authors used the YCG1-AID strain in the experiment described in lines 160-174. This is not trivial, because a previous study in fission yeast used a kleisin mutant (cnd2-1) and reached a different conclusion from that reported here.

While Cnd2 from fission yeast is functionally homologous to Brn1 from budding yeast, our results show that late replication origins were efficiently repressed in the presence of hydroxyurea (HU) regardless of whether we used the pMET-YCG1-AID strain (**Supplementary Figure 2b**) or the *smc2-8* mutant (**new Supplementary Figure 2d**). These findings indicate that condensin is not required for activation of the DNA replication checkpoint in budding yeast, a phenotype that contrasts with the requirement

observed in fission yeast. Incidentally, we also show that condensin II is dispensable for CHK1 activation in human cells (**Fig. 4c**).

(2) The description that "condensin-mediated DNA overwinding may be crucial for chromosome decatenation by topoisomerase II" (lines 206-207) may need to be rephrased. For example, Ref 34 reports negative supercoiling, not overwinding, of DNA induced by budding yeast condensin. This reviewer recognizes that the issue discussed here is not easily resolved, but the current description is misleading and needs to be corrected.

We agree that this is a complex issue. We have now rephrased this sentence to read "condensin-mediated DNA supercoiling has been proposed to facilitate chromosome decatenation by topoisomerase II" without specifying whether the supercoiling is positive or negative.

(3) Figure 3 provides evidence that Smc2 and Smc4 travel with replication forks. It would be nice to show that condensin II-specific subunits, but not condensin I-specific subunits, behave accordingly.

We now provide evidence that CAPD3 (condensin II) is slightly enriched at replication forks whereas CAPG (condensin I) is not (**new Fig. 4a**).

(4) Similarly, it is very important to demonstrate that the essential role in fork restart is ascribed to condensin II, but not condensin I. For example, siCAPG is missing from Figure 3d, and the text is generally inadequate to explain this important point.

Thank you for suggesting this important control. We now provide evidence that unlike CAPG2 (**Fig. 4d**), depletion of CAPG does not affect fork restart (**new Supplementary Fig. 4b**).

(5) The depletion experiments in human cells reported in the current manuscript use RNA interference, which inevitably requires a long depletion time (48 hr). The authors should discuss the potential drawback of this technique, which is becoming less popular in the field.

We agree that RNA interference has certain limitations; however, it remains the most practical approach when performing multiple depletions, as in many of the experiments described in this study. To validate the role of condensin in fork resection using an alternative depletion method, we employed Smc4-mAID-Halo cells (Schneider *et al*., Nature, 2022) and induced SMC4 degradation by treating cells with 5-PhIAA for 2.5 hours. As shown in the **new Supplementary Fig. 4f**, this analysis confirmed that fork resection is impaired in the absence of the SMC4 subunit common to both condensin complexes.

(6) The authors conclude that "condensin acts with topoisomerase I at reversed forks to convert positively supercoiled DNA into structures that are subsequently relaxed by topoisomerase II" (line 45-; line 410-). It is not very clear what is meant by "structures that are subsequently relaxed by topoisomerase II" means. Do they correspond to positive plectomenes as shown in **Figure 7**? The authors need to be more explicit on this point.

Yes, these structures correspond to positive plectonemes, as shown in **Fig. 7**. This is now better explained in the text.

(7) "The activity would differ from its mitotic role…." (line 477). This reviewer understands the logic here, but are the mitotic and interphase activities discussed here indeed mechanistically different? Is

there an explanation that possibly integrates the mitotic and interphase activities discussed here? The authors may want to revisit this point and possibly rephrase it.

We thank the reviewer for this insightful comment. We have revised the text to clarify that we do not propose a mechanistic difference between the mitotic and interphase activities of condensin. Rather, we assume that condensin acts through the same molecular activity in both contexts, but is deployed for different functional purposes. In both interphase and mitosis, condensin likely contributes to the relief of torsional stress by promoting TOP2 activity. The distinction we now emphasize is therefore contextual rather than mechanistic, highlighting a role for condensin outside of mitosis rather than a fundamentally different mode of action.

Reviewer #4 (Remarks to the Author):

The study by Da Mota et al focuses on the functions of condensin and DNA topoisomerases during replication stress in budding yeast and human cells. Their initial results show that condensin is enriched at early replication origins in budding yeast cells cultured in hydroxyurea (HU). The enrichment of condensin around stalled replication forks requires Rad50, Gcn5 and Chd1.

The authors then allow cells to incorporate BrdU under HU conditions and perform DNA combing in the absence and presence of condensin subunits. The results show that in the absence of condensin function, shorter BrdU-DNA tracks are found following prolonged incubation in HU-media. The authors speculate that this is due to defects in fork restart in the absence of condensin function, and design an experiment to monitor fork restart following a pulse of MMS. Also, during the recovery from MMS, the absence of condensin function leads to shorter labelled DNA tracks.

Interestingly, the simultaneous depletion of Top1 rescues the short-labelled DNA tracks in the absence of condensin function, whereas simultaneous depletion of Top2 leads to even shorter labelled DNA tracks than after either condensin or Top2 depletion alone (Fig. 2d). The authors then analyze if condensin acts at replication forks in human cells. They find that condensin II promotes replication fork restart at low concentration of HU (Fig. 3d). Unlike in yeast, at high concentration HU in human cells, knockdown of condensin II resulted in faster replication speed than in control cells (Fig. 3e). The authors speculate that condensin II might be required for slowing the DNA replication in human cells, in response to high HU concentrations. The authors then aim to analyze for resectioning by first subjecting cells to consecutive pulses of IdU and CldU, and then adding low concentration of HU, which may induce fork resectioning and thereby shortening of the later CldU track. This appears to occur in control cells following HU treatment as the CldU/IdU ratio decreases. However, in condensin II (but not condensin I) depletion condition the CldU/IdU ratio is not altered by HU addition (Fig. 3f). The author states that this indicates that condensin II is essential for fork resectioning in HU conditions.

Condensin II is then shown to promote fork reversal by direct visualization of replication intermediates by electron microscopy (Fig. 4b). The interplay between condensin II and DNA topoisomerases or SMARCAL1 is then investigated. The authors then switch back to yeast, and unlike previous experiments, decide to use Top1 deleted cells to monitor RPA enrichment around early firing origins. They find that in Top1 deleted cells high enrichment of RPA is detected, which is not altered when condensin is depleted. In human cells, however, double depletion of condensin II and

Top1 leads to considerably higher chromatin-bound RPA. The manuscript ends with an attempt to present a model that encompasses condensin's and DNA topoisomerases' functions at stalled replication forks.

The amount of data presented in the manuscript is extensive, however, several important controls are missing. The amount of data presented, together with the differences in action between yeast condensin and condensin II in human cells, makes it difficult to present a clear and logical model in the end. In addition, throughout the manuscript, the authors speculate about the supercoiling state behind and ahead of stalled replication forks in a manner that does not seem reasonable.

The main points that need to be are addressed are:

1) How are the authors convinced that the effects of condensin and topoisomerase depletion stems from their function in S-phase?
In yeast, it could be that the tagging of condensin subunits or DNA topoisomerases makes them partially non-functional and therefore initiates S-phase with some linguiring problems. The authors should perform experiments in the absence of auxin to show that this is not the case.

Thank you for giving us the opportunity to clarify this important point in the revised manuscript. We now include flow cytometry analyses of S-phase progression with or without auxin (**new Supplementary Fig. 3b**), along with corresponding western blots (**new Supplementary Fig. 3c**), showing no detectable differences between WT, Smc4-AID, Top1-AID, and Top2-AID strains. In addition, DNA combing analyses demonstrate that the AID tag does not affect fork progression in the absence of auxin (**new Supplementary Fig. 3d-e**).

The authors instead express WT SMC4 in the SMC4-AID strain. What is this a control for?

We supplemented SMC4-AID cells with wild-type SMC4 (**Fig. 2b**) to confirm that the auxin-induced fork slowdown results specifically from SMC4 depletion. We also attempted to complement the depleted cells with an inactive SMC4 variant (SMC head engagement mutant: *smc4*^{S1324R} mutant (Thadani R. *et al*, cell Reports 2018); see **Fig. II for reviewers**). However, the mutation noticeably reduced protein stability, making the interpretation of this rescue experiment unreliable. For this reason, we chose not to include these additional data in the revised manuscript.

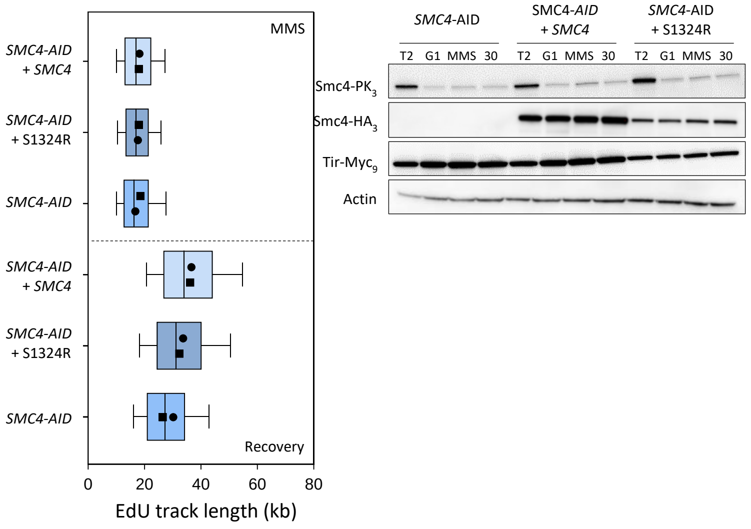

Fig. II for reviewers: Condensin is required for timely fork restart after MMS exposure. Wild type, *SMC4*-PK_3-AID, *SMC4*-PK_3-AID + *SMC4*-HA_3 and *SMC4*-PK_3-AID + *smc4*^{S1324R}-HA_3 cells were synchronized in G_1 with α-factor and released into S phase in the presence of 0.05% MMS. Auxin was added 60 minutes before release from the G_1 arrest. Cells were released for 45 minutes into S phase in the presence of MMS and EdU, then MMS was removed to let cells recover for 30 minutes. (Left) The length of EdU tracks was determined after 45 minutes in MMS and after recovery. The distribution of EdU tracks length is shown for three independent experiments. Box and whiskers indicate 25th–75th and 10th–90th percentiles, respectively. Mean length is indicated. **: p<0.01; *: p<0.05; ns: non-significant (unpaired t-test on means). (Right) Western blot analysis of *SMC4*-PK_3 degradation and Smc4-HA3 or *smc4*^{S1324R} expression.

In human cells, are the authors certain that the 48 hour depletion of condensin II does not cause additional effects which affects the cells ability to respond to HU?
The authors should perform experiments using AID-tagged condensin II (+/- auxin) following release from a G1/S-block to ensure that the detected findings stems from condensin II's function in S-phase.

As indicated above in our response to Reviewer #2, we now show that a short depletion (2.5 hours) of SMC4 in Smc4-mAID-Halo cells (Schneider *et al.*, Nature, 2022), is sufficient to deplete SMC4 and confirm that condensin is required for efficient fork resection (**new Supplementary Fig. 4f**).

2) Throughout the manuscript, the authors speculate about the DNA supercoiling state surrounding stalled replication forks, although no direct evidence is presented. The authors should consider to substantially reduce the amount of speculation, and address the questions below.
For example,
"Our findings suggest that condensin acts with topoisomerase I at reversed forks to convert positively supercoiled DNA into structures that are subsequently relaxed by topoisomerase 2, allowing the fork to resume replication."

How would Top1 convert positively supercoiled DNA into structures that are relaxed by Top2?
Top1 relaxes positively supercoiled DNA.

We thank the reviewer for highlighting this point and agree that the original wording could be misleading. We have revised the text to clarify the respective roles of TOP1, condensin, and TOP2, and to reduce speculative statements. As indicated in our response to Reviewer #1, we have simplified our model to focus on positive DNA supercoiling occurring during fork reversal (see **new Fig. 7**).

TOP1 directly relaxes positively supercoiled DNA and does not by itself convert positive supercoils into a substrate for TOP2. Rather, based on published work, TOP1 is required for productive condensin activity through its participation in the "pinch-and-merge" mechanism of condensin-mediated loop extrusion. During this process, condensin generates torsional stress and local supercoiling as it pinches off and merges DNA loops. TOP1 relieves the negative supercoiling generated during these steps via transient single-strand cleavage and controlled DNA rotation, thereby allowing condensin to continue its activity. Through this cooperation, condensin, whose function depends on TOP1, produces higher-order DNA structures and torsional configurations that subsequently require TOP2 for their resolution. Thus, it is not TOP1 acting alone, but the coordinated action of condensin and TOP1 that generates DNA substrates ultimately resolved by TOP2. We have rephrased the relevant section to better reflect this mechanistic framework and to avoid implying a direct conversion of positive supercoils by TOP1 into TOP2 substrates.

"The negative superhelical tension generated at reversed forks by reannealing of parental DNA strands is also a good substrate for TOP1."
How and where is the negative superhelical tension generated by reannealing the parental strands?
Negative superhelical tension is created by unwinding double-stranded DNA.

As mentioned above, we have simplified our model to remove this element, which was indeed too speculative and insufficiently supported by our data. Nonetheless, in a topologically constrained context, reannealing of single-stranded parental DNA during fork reversal would be expected to generate negative supercoils and additional ssDNA. This aspect of the model will be investigated in future work.

“Since the positive torsional stress that accumulates ahead of the fork during DNA replication and drives fork reversal is resolved by TOP2A6,7, we investigated whether condensin II collaborates with TOP2A in fork reversal by assaying fork slowdown and fork resection as indicators of fork reversal in cells depleted of CAPG2, TOP2A or both proteins.“

The suggestion that positive torsional stress that accumulates ahead of the fork during DNA replication drives fork reversal is a speculation, and should be referred to as such.

Thank you very much for pointing this out. The original sentence was indeed misleading as it mixed up two distinct concepts. (i) Upon TOP1 inhibition, positive torsional stress accumulates ahead of the fork and acts as a mechanical driver of fork reversal (Ray Chaudhury et al. NSMB 2012). (ii) Fork reversal itself generates positive torsional stress that creates a topological barrier, which must be relieved to allow further extension of the reversed arm. This phenomenon was first observed in bacteria (Fierro-Fernandez, 2007) and later confirmed in human cells (Tian, 2021). The sentence highlighted by the Reviewer essentially refers to the second concept and has now been revised as follows: “Since TOP2A resolves positive torsional stress during fork reversal, we examined whether condensin II …”

In the legend of Fig. 7, the authors speculate that:
“a Elongation of DNA replication generates positive supercoils (Sc+) in front of the forks that are normally relaxed by TOP1 and precatenanes behind the fork that are resolved by TOP2. b Upon fork arrest, Sc+ can be transferred behind the fork via a process called fork reversal, in which nascent DNA strands reanneal to form a regressed arm.”

If positive supercoils are transferred behind the replication fork, the structure that is created behind the forks would be pre-catenanes, not positive supercoiling.

It is important to distinguish two separate processes. First, during replication fork progression, positive supercoils accumulate ahead of the fork and can be relaxed either by TOP1 or by fork rotation, which generates precatenanes behind the fork. Second, when a replication fork stalls and reverses, positive DNA supercoiling arises from the separation of parental and newly synthesized strands, independently of precatenane formation. This distinction is now explained more clearly in the legend of **Fig. 7**.

Also, since fork reversal (might) occur during HU treatment, when replication forks move very slowly, is it reasonable to consider that any substantial positive supercoiling occurs ahead of the forks under these conditions? The author could test this by depleting Top1 and Top2 in HU-conditions to see if replication forks are stopped.

We thank the reviewer for this thoughtful comment. Fork reversal has been directly visualized by electron microscopy and shown to be a widespread cellular response to replication stress induced by multiple genotoxic agents, including HU, even under conditions in which replication fork progression is strongly reduced (Zellweger, 2015). It is therefore likely that accumulation of positive supercoiling ahead of the fork, such as that induced by TOP1 depletion, contributes to but is not strictly required for fork reversal under HU treatment.

With respect to the experiment suggested by the reviewer, although depletion of either TOP1 or TOP2 alone is partially tolerated, combined depletion of both enzymes is not viable in either yeast or human cells, precluding a direct test of fork arrest under these conditions. Nevertheless, we assessed

replication fork restart following transient HU treatment (4 mM HU for 3 hours) in cells depleted for either TOP1 or TOP2A. In both cases, we observed a marked defect in fork restart, supporting the idea that topoisomerase activity is required for efficient recovery of stalled or reversed forks under HU-induced replication stress (**new Fig. 6a**).

Additional points,

1) Why is Top1 deletion used in Fig. 6, when Top1-AID was used in Fig 2. It makes it difficult to compare the results. The authors should perform the RPA enrichment analysis in TOP1-AID cells with or without auxin.

As requested by this referee, we have performed the RPA enrichment analysis in TOP1-AID cells with or without auxin. As shown in the figure below, we also observed increased RPA recruitment to *ARS306*, although to a lesser extent as in *top1Δ* cells (**Fig. III for reviewers**).

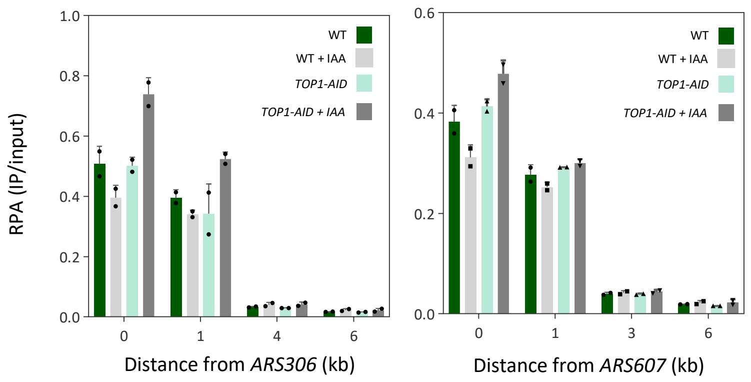

Fig. III for reviewers: Acute depletion of Top1 leads to RPA accumulation at HU-arrested forks in yeast cells. WT and Top1-AID cells were synchronized in G_1. Sixty minutes before release into S phase, cultures were split, and half of the cells were treated with auxin to induce Top1 depletion. Cells were then released into S phase in the presence of 200 mM hydroxyurea (HU). RPA enrichment at increasing distances from the early origins ARS306 and ARS607 was determined by ChIP-qPCR and is shown as the signal ratio (IP/Input). Data represent the mean ± SD of two independent experiments.

2) The authors reach the conclusion that the simultaneous depletion of Smc4 and Top2 had no additional effect on EdU track length following MMS exposure.
"By contrast, simultaneous depletion of Smc4 and Top2 had no additional effect on fork recovery when compared with depletion of these proteins individually, indicating that condensin and Top2 may function in a common pathway required for efficient fork restart following replication stress"

However, in Fig. 2d there is an additional effect. Can the authors explain their reasoning?

As discussed in our responses to **Reviewer #1**, we have performed an extensive analysis comparing differences within individual experiments and between the means of biological replicates (n=3) across all superplots. This analysis shows that the small differences observed between cells lacking Smc4, Top2, or both proteins are not statistically significant when comparing the means of biological replicates (**new Fig. 3b**). A supplementary table (**new Table 1**) with all statistical tests is also provided.

3) non-calibrated, non-quantitative ChIP-seq is presented in the manuscript. To validate the findings, quantitative ChIP-qPCR experiments are presented alongside.
However, normalizing the data to an unreplicated region makes it impossible for the reader to

appreciate the quality of the ChIP-experiment and understand the signal-to-noise ratio. This refers to the data in Fig. 1e, 1f and S1f. This is especially important since condensin is enriched in certain unreplicated regions.

Fig. 6a and S6a also show ChIP-qPCR data normalized to unreplicated regions. Also, these data should be presented without normalization so that the readers can assess the quality and reproducibility of the ChIP-qPCR experiment.

Thank you very much for pointing this out. We now display the percentage of IP/input without normalization on a negative zone in **Supplementary Figs. 1d, 1f-h and 2h-I and 3j.**

4) In the absence of HU, no effect on replication is detected when condensin is inactivated. However, it is not shown that condensin is absent when the EdU is added (S2e). Can the lack of replication slowdown be due to the short depletion of condensin under these conditions?
Perhaps smc2-8 be analyzed to avoid this risk?

We now provide evidence that the condensin subunit Ycg1 is fully degraded at the time of EdU addition (**new Supplementary Fig. 2f**).

5) What is the benefit of including Mirin? It does result in any difference.

In EM experiments, mirin is typically used to prevent the degradation of the reversed arm by MRE11. This is now indicated in the figure legend.

REVIEWERS' COMMENTS

Reviewer #1 (Remarks to the Author):

The authors have carefully and successfully addressed the comments raised. Despite the origin of RPA-coated ssDNA remaining unclear, the authors have done their best to clarify this point in the revised version by showing that it contributes to cell sensitivity to replication stress in the absence of functional condensin.

Attention could be given to few mistakes:

Line 121: ‘HU-arrested forks’ could be replaced by “HU-stalled forks”.
Lines 218-218: “rfa1(G77A)” and figure 2, the allele nomenclature may be changed change. rfa1-G77E or rfa1G77E as originally published in ref 31.
Line 324: “failed to undergo nascent DNA under these conditions”, the word resection is likely missing.
Line 413: “SMARCAL1 was dispensable for fork slowing and resection TOP1-deficient cells” In TOP1…
Line 529 “with a role for these factors in of parental DNA strand reannealing” remove of ?

We thank the reviewer for their positive comments and for highlighting these additional errors, which we have now corrected.

Reviewer #2 (Remarks to the Author):

I co-reviewed this manuscript with one of the reviewers who provided the listed reports. This is part of the Nature Communications initiative to facilitate training in peer review and to provide appropriate recognition for Early Career Researchers who co-review manuscripts.

Reviewer #3 (Remarks to the Author):

The revised manuscript has successfully addressed the concerns I raised in the first round of reviews. While I am still wondering why fission yeast behaves differently from budding yeast and humans, this issue may be beyond the scope of the present work. I would simply suggest rephrasing the sentence in the summary from "we show in yeast and human cells" to we show in budding yeast and human cells" (line 43).

We thank Reviewers #2 and #3 for their constructive feedback, which has improved our manuscript. We have rephrased the summary as requested and added multiple references to "budding yeast" throughout the Discussion section.

Reviewer #4 (Remarks to the Author):

I thank the authors for the detailed rebuttal. The new model presented in Figure 7 focuses on the results from human cells. Would it be possible for the authors to also incorporate the findings from budding yeast?

Thank you very much for these constructive comments. We now indicate that the model shown in Fig. 7 specifically refers to human cells. We have also added the following sentences in the figure legend to stress the differences between yeast and human cells: “Whether a similar mechanism operates in budding yeast remains unclear. Indeed, reversed arm extension is more limited than in human cells, and alternative pathways for parental DNA reannealing may be equally important. Further work is needed to elucidate how condensin and topoisomerase cooperate to promote fork restart in this unicellular organism”.

For example, the rfa1(G77E) mutation fully suppresses the HU sensitivity of smc2-8 cells. This new finding is striking and interesting; however, it is not currently considered in the final model. Could the authors clarify their interpretation of this result?

We now speculate in the discussion section that RPA prevents the reannealing of parental DNA strands: “Interestingly, weakening RPA-ssDNA binding with the *rfa1-G77E* allele[31] suppressed the HU sensitivity of the *smc2-8* mutant, supporting the view that excess RPA-coated ssDNA is toxic, presumably by preventing the reannealing of parental DNA strands during fork restart.”

In relation to the above, RPA levels at stalled forks do not appear to be directly linked to fork recovery, since the top1Δ SMC4-AID double mutant clearly shows higher RPA levels than WT but displays no defect in fork restart.

Indeed, we believe that ssDNA resulting from the resection of nascent DNA is functionally distinct and less problematic that ssDNA resulting from the impaired reannealing of parental DNA, but further work is required to formally establish this difference.

This further strengthens the need to clarify why the rfa1(G77E) mutation fully suppresses the condensin mutant’s HU sensitivity. The authors should test whether the rfa1(G77E) allele rescues the smc2-8 defect in fork restart. This would help clarify the role of RPA-coated ssDNA in condensin activity at stalled replication forks.

We thank the reviewer for this excellent suggestion, but performing this experiment would take at least a month and may not be entirely conclusive, so we will save it for our next study.

Minor point:
I would recommend softening the statements in the Introduction suggesting that fork reversal is initiated by the accumulation of positive supercoiling, and that extension of the reversed arm generates positive supercoiling along sister chromatids, as these interpretations are speculative.

Indeed, this statement was overly speculative. We have rephrased it as: “Fork reversal is influenced by local topological context. In principle, positive supercoiling ahead of the fork promotes reversal[5], while positive supercoiling along sister chromatids during reversed arm extension restrains it[6]. However, this aspect remains largely unexplored *in vivo*, despite recent evidence that DNA topoisomerase II (TOP2) can relieve this constraint[7].”
